# Leveraging heterogeneity for neural computation with fading memory in layer 2/3 cortical microcircuits

**DOI:** 10.1371/journal.pcbi.1006781

**Published:** 2019-04-25

**Authors:** Renato Duarte, Abigail Morrison

**Affiliations:** 1 Institute of Neuroscience and Medicine (INM-6), Institute for Advanced Simulation (IAS-6) and JARA Institute Brain Structure-Function Relationships (JBI-1 / INM-10), Jülich Research Centre, Jülich, Germany; 2 Bernstein Center Freiburg, Albert-Ludwig University of Freiburg, Freiburg im Breisgau, Germany; 3 Faculty of Biology, Albert-Ludwig University of Freiburg, Freiburg im Breisgau, Germany; 4 Institute of Adaptive and Neural Computation, School of Informatics, University of Edinburgh, Edinburgh, United Kingdom; 5 Institute of Cognitive Neuroscience, Faculty of Psychology, Ruhr-University Bochum, Bochum, Germany; Northeastern University, UNITED STATES

## Abstract

Complexity and heterogeneity are intrinsic to neurobiological systems, manifest in every process, at every scale, and are inextricably linked to the systems’ emergent collective behaviours and function. However, the majority of studies addressing the dynamics and computational properties of biologically inspired cortical microcircuits tend to assume (often for the sake of analytical tractability) a great degree of homogeneity in both neuronal and synaptic/connectivity parameters. While simplification and reductionism are necessary to understand the brain’s functional principles, disregarding the existence of the multiple heterogeneities in the cortical composition, which may be at the core of its computational proficiency, will inevitably fail to account for important phenomena and limit the scope and generalizability of cortical models. We address these issues by studying the individual and composite functional roles of heterogeneities in neuronal, synaptic and structural properties in a biophysically plausible layer 2/3 microcircuit model, built and constrained by multiple sources of empirical data. This approach was made possible by the emergence of large-scale, well curated databases, as well as the substantial improvements in experimental methodologies achieved over the last few years. Our results show that variability in single neuron parameters is the dominant source of functional specialization, leading to highly proficient microcircuits with much higher computational power than their homogeneous counterparts. We further show that fully heterogeneous circuits, which are closest to the biophysical reality, owe their response properties to the differential contribution of different sources of heterogeneity.

## Author summary

Cortical microcircuits are highly inhomogeneous dynamical systems whose information processing capacity is determined by the characteristics of its heterogeneous components and their complex interactions. The high degree of variability that characterizes macroscopic population dynamics, both during ongoing, spontaneous activity and active processing states reflects the underlying complexity and heterogeneity which has the potential to dramatically constrain the space of functions that any given circuit can compute, leading to richer and more expressive information processing systems. In this study, we identify different tentative sources of heterogeneity and assess their differential and cumulative contribution to the microcircuit’s dynamics and information processing capacity. We study these properties in a generic Layer 2/3 cortical microcircuit model, built and constrained by multiple sources of experimental data, and demonstrate that heterogeneity in neuronal properties and microconnectivity structure are important sources of functional specialization, greatly improving the circuit’s processing capacity, while capturing various important features of cortical physiology.

## Introduction

Heterogeneity and diversity are ubiquitous design principles in neurobiology (or in any biological system, for that matter), covering components and mechanisms at every descriptive scale [1]. While many of these specializations, as well as their inherent complexity and diversity, are functionally meaningful, intrinsically linked and responsible for the brain’s computational capacity and efficiency (see e.g. [2–4]), others are bound to reflect epiphenomena, by-products of evolution, bearing little or no functional significance [5], or to subserve metabolic/maintenance tasks [6] that, while crucial for healthy function, are not directly involved in the computational process.

In order to study the functional role of heterogeneity in cortical processing, we need to modularize complexity [7]: exploit the degenerate nature of the system [8, 9] and heuristically identify groups of components that may behave as singular modules (depending on the scale and processes of interest). Once these tentative ‘building blocks’ are identified, we need to specify adequate levels of descriptive complexity that may shed light onto the underlying functional principles. These pursuits, however, pose severe epistemological problems as we currently have no clear intuition as to what ‘adequacy’ means in this context (see, e.g. [10–12]).

Despite substantial progress, our ability to clearly identify the system’s core component ‘building blocks’ [3, 13] and to systematically characterize their relative contributions and potential functional roles is still a daunting task given the multiple spatial and temporal scales at which they operate, their complex, nested interactions and the, often incomplete or inconsistent, empirical evidence. Nevertheless, when studying neuronal computation, one needs to keep in mind that, despite its tremendous complexity or because of it, the brain is a machine ‘fit-for-purpose’ and optimized to process information and operate in complex, dynamic and uncertain environments, whose spatiotemporal structure it must extract in order to reliably compute [1, 4]. As such, it is important to disentangle and quantify which and how the different components of the system may modulate functional neurodynamics in meaningful ways that can be paralleled with related experimental observations, and to which degree these specializations affect the system’s operations.

In the next section, we attempt to identify an appropriate partition of potential building blocks of complexity and heterogeneity in neocortical microcircuits. We then proceed to building a biologically inspired and strongly data-driven microcircuit model that explicitly employs this partition in an attempt to understand and quantify the differential functional roles and consequences of these different sources of heterogeneity applying systematic and generic benchmarks. We will focus on generic properties in both the microcircuit architecture and its information processing capacity. Rather than modelling specific microcircuits in specific cortical regions and corresponding functional specializations (as in e.g. [14, 15]), we generalize our approach and collate data pertaining to layer 2/3 microcircuit features (anatomical and physiological) from different cortical regions, while evaluating the differential contributions of different forms of heterogeneity on the system’s dynamics and generic computational properties, focusing particularly on their suitability for continuous, online processing with fading memory [16–18]. The approach also aims at providing an intermediate level of descriptive complexity for studying computation in cortical microcircuit models, as discussed below.

### Heterogeneous building blocks in the neocortical circuitry

On a macroscopic level, hierarchical modularity is easily identifiable as a parsimonious design principle underlying various structural and functional aspects of cortical organization [19–23]. Different anatomophysiological [24, 25], genetic / biochemical [26–30] or functional [31–33] criteria give rise to slightly different modular parcellations but, in combination, these criteria reveal the relevant ‘building blocks’, the most important features whose variations and recombinations give rise to the complexity and diversity of the cortical tissue [34].

For convenience, these features can be coarsely (and tentatively) grouped into neuronal, synaptic and structural components (see also [13]). Neuronal features refer to the different cell classes and their laminar and regional distributions [35] along with their characteristic electrophysiological and biochemical diversity [36, 37]. Synaptic components refer to a molecular default organization characterized by variations in the differential expression and transcription of genes involved in synaptic transmission [26, 28], which is reflected, for example, in regional receptor architectonics [38–40]. Structural aspects include variations in cortical thickness and laminar depth [41] along with neuronal and synaptic density [42, 43] and input-output (both local and long-range) connectivity patterns [44, 45]. In combination, these features highlight default organizational principles whose variations across the cortical sheet are likely to contribute to the corresponding functional specializations.

Based either on morphological, electrophysiological or biochemical features (or, preferably, a combination thereof), several different classes of neurons can be identified throughout the neocortex (see e.g. [35–37, 46–48]). Apart from pronounced regional and laminar differences in the types of neurons that make up the cortex and their relative spatial distributions, every microcircuit in every cortical column is composed of diverse neuron types, with heterogeneous properties and heterogeneous behaviour.

Electrochemical communication between these diverse neuronal classes is an intricate, dynamic and very complex process involving a multitude of nested inter- and intracellular signalling networks [49–51]. Their functional range spans multiple spatial and temporal scales [52–54] and has, arguably, the most critical role in modulating microcircuit dynamics and information processing within and across neuronal populations [2, 3, 55]. The specificities of receptor composition and kinetics underlie the substantial diversity observed in the elicited post-synaptic potentials [56, 57] across different synapse and neuronal types (see, e.g. [58–62]). This occurs because the receptors mediating these events have distinct biochemical and physiological properties depending on the type of neuron they are expressed in and, naturally, the type of neurotransmitter they are responsive to. These varying properties have known and non-negligible implications in the characteristic kinetics of synaptic transmission events occurring between different neurons [63] and strongly constrain the circuit’s operations.

Additionally, cortical microcircuits are not randomly coupled, but over-express specific connectivity motifs [64–70], which bias and skew the network’s degree distributions [71] and/or introduce correlations among specific connections [72], thus selectively modifying the impact of specific pre-synaptic neurons on their post-synaptic targets. Analogously to the heterogeneities in neuronal and synaptic properties, such structural features are known to significantly impact the circuit’s properties [73–77].

### Descriptive adequacy

As discussed above, the variations in the anatomy and physiology of a cortical microcircuit are experimentally well established and have been shown to influence computational properties. However, the absence of complete descriptions of biophysical heterogeneity and well-substantiated empirical evidence to support them (primarily due to technical limitations), has forced computational studies to take a simplified approach. This has multiple additional advantages, such as greater likelihood of analytical tractability and lower overhead for the researcher in specifying the network parameters. Simplified, homogeneous models of spiking networks have proven to be a valuable tool for a theoretically grounded exploration of microcircuit dynamics, emerging from the interaction of excitatory and inhibitory populations [78–81].

The generic principles established by studying simple balanced random networks have subsequently been applied to model specific cortical microcircuits with integrated connectivity maps and realistic numbers of neurons and synapses [82]. This approach revealed that some prominent features of spontaneous and stimulus-evoked activity and its dynamic flows through a cortical column can be accounted for by the macroscopic connectivity structure, mediated by local and long-range interactions [83]. However, by focusing on emergent dynamics, these studies neglect the functional aspects and the fact that cortical interactions serve computational purposes (but, see [84] for a study on the computational properties of the [82] microcircuit model). In addition, although there are good reasons for taking a minimalist approach, assuming uniformity and homogeneity on every component of the system tends to lack cogency with respect to established anatomical and physiological facts and to disregard biophysical and biochemical plausibility.

Some of these limitations were circumvented by [85], who not only accounted for detailed and empirically-informed connectivity maps, but also employed more biologically motivated models of neuronal and synaptic dynamics and placed them in an explicit functional/computational context. In line with the results obtained with the simpler microcircuit models [82, 84], this study demonstrated that considering realistic structural constraints is beneficial and significantly improves the computational capabilities of the circuit. Several other studies provide important steps to move away from homogeneous systems by incorporating variability (e.g. [75, 86, 87]), but tend to do so in a relatively arbitrary manner and/or focusing on specific forms of heterogeneity while retaining homogeneity in other components (depending on the scientific objectives of the study).

A completely different set of priorities for modelling cortical microcircuits are espoused by [88] in the framework of the continuous efforts of the Blue Brain project [89]. The Blue Brain approach lies on the other extreme of the descriptive scale, in that it attempts to model a cortical column in full detail, explicitly accounting for the complexities of cellular composition (based on neuronal morphology and electrophysiology), synaptic anatomy and physiology, as well as thalamic innervation, essentially constituting an *in silico* reconstruction of a cortical column (see also [90]). This approach is, naturally, extremely computationally expensive and its explanatory power is limited. The model complexity at this end of the spectrum is so close to the biophysical reality that it might not lend itself to a comprehensive understanding of dissociable and important functional principles any more readily than studying the real thing does. Nonetheless, it provides valuable insights in that it carefully replicates a lot of *in vivo* and *in vitro* responses of a real cortical column, while generating a wealth of complete and comprehensive data [88, 91].

Thus, we conclude that while simpler models are preferable, as they are generic enough to be broadly insightful and allow us to uncover general principles, we should ask the question: what is the cost of simplification? If a model simplifies away the core computational elements of the system, our ability to account for its operations is lost. The findings discussed above indicate that heterogeneity may be critical for the mechanisms of computation; therefore models aiming at uncovering computational principles in specific biophysical systems, such as a cortical column or microcircuit, should account for these features.

In this study, we attempt to bridge this descriptive gap by building microcircuit models, inspired and constrained by the composition of Layer 2/3, that account for key heterogeneities in neuronal, synaptic and structural properties. We implement all types of heterogeneity such that they can be switched on or off, thus enabling us to systematically disentangle and evaluate the roles played by the different types of heterogeneity in the different tentative building blocks, and how they collectively interact to shape the circuit’s dynamics and information processing capacity.

The choices and characteristics of the models and parameter sets used throughout this study, as well as the general microcircuit composition are constrained and inspired by multiple sources of experimental data (see section *Data-driven microcircuit model* and S1 Appendix) and account for the prevalence of different neuronal sub-types and their heterogeneous physiological and biochemical properties, the specificities of instantaneous synaptic kinetics and its inherent diversity as well as specific structural biases in cortical micro-connectivity. All models and model parameters were, as far as possible, chosen to directly match relevant experimental reports and minimize the introduction of arbitrary model parameters, in order to ensure that the effects observed are caused by realistic forms of complexity and heterogeneity and avoid imposing excessive assumptions or preconceptions on the systems studied, i.e. to “allow biology to speak for itself”.

In section *Data-driven microcircuit model* (complemented by the Methods section and the Supplementary Materials), we explain all the details of the models and model parameters used to build and constrain the microcircuit, as well as the underlying empirical observations that motivate the choices. After specifying and fixing all the relevant parameters to, as closely as possible, match multiple sources of empirical data, we study the effects of heterogeneity on population dynamics in a *quiet* state, where the circuit is passively excited by background noise (section *Emergent population dynamics*) and in an *active* state, where the circuit is directly engaged in information processing (section *Active processing and computation*). We evaluate the circuit’s sensitivity and responsiveness, as well as its memory and processing capacity, demonstrating a clear and unambiguous role of heterogeneity in shaping the proficiency of the system by greatly increasing the space of computable functions.

## Results

### Data-driven microcircuit model

In this section, we describe the process of building a complex data-driven cortical microcircuit model capturing some of the fundamental features of layer 2/3. We specify the detailed architecture, composition and dynamics of the microcircuits explored throughout this study as well as the motivation behind all model and parameter choices. In each relevant section, we highlight the differences between the respective homogeneous and heterogeneous conditions. A summarized, tabular description of the main models is provided in S1 Table, along with a list of the primary sources of experimental data used to constrain the model parameters, provided in S1 Appendix.

All the circuits analysed throughout this study are composed of N = 2500 point neurons (roughly corresponding to the size of a layer 2/3 microcircuit in an anatomically defined cortical column; [92]), divided into N_E_ = 0.8 N excitatory, glutamatergic neurons and N_I_ = 0.2 N inhibitory, GABAergic neurons. In addition, we further subdivide the inhibitory population in two sub-classes, I_1_ and I_2_ (with $\;N_{I_{1}}=0.35\;N_{I}$ and $\;N_{I_{2}}=0.65\;N_{I}$), corresponding to fast-spiking and non-fast-spiking interneurons, respectively (see *Neuronal properties*). Accordingly, there are nine different synapse types (all possible connections between neuronal populations), with distinct, specific response properties (see *Synaptic properties*). Similarly, there are nine connection probabilities from which random connections are drawn (see *Structural properties*).

For each of the key features of neuronal, synaptic and structural properties, we differentiate between the homogeneous case, where all properties are identical, and the heterogeneous case, where properties are drawn from appropriately chosen distributions. In this way, we can tease apart the differential effects of the three sources of heterogeneity considered here: neuronal, synaptic and structural.

For consistency, all the circuits’ structural (and synaptic) features are constrained primarily by the composition of layer 2/3 in the C2 barrel column in the mouse primary somatosensory cortex (S1), given the availability of direct, complete and significantly explored experimental datasets (e.g. [92–94]).

#### Neuronal properties

We divide the neurons into one excitatory (E: glutamatergic, pyramidal neurons) and two inhibitory (I_1_: GABAergic, fast spiking interneurons, I_2_: GABAergic, non-fast spiking) classes, consistent with the reports in [92, 93] and [94]. The three classes differ in relative excitability and firing properties, providing substantially more electrophysiological diversity than commonly exhibited in cortical models on the abstraction level of point neurons (e.g. [81, 82, 95–97]), while still being of a manageable degree of complexity. All neurons are modelled using a simplified adaptive leaky integrate-and-fire scheme (see Methods and [98, 99]).

The parameters used for the different neuron types (summarized in Table 1) were chosen to match the respective ranges reported in the literature, considering both the data collected in the NeuroElectro database [36, 100] encompassing tens of unique data points from different experimental sources (see S1 Appendix) and those reported in [92, 93, 101, 102], and [94] given the completeness of these reports and the direct similarities with our case study.

**Table 1:** Single neuron parameter sets. In the heterogeneous condition, three neuronal types have the values of the described parameters randomly drawn from a normal (N) or lognormal (logN) distribution. The parameter values for these distributions were determined taking into account multiple sources of experimental data (see S1 Appendix). Note that, to make comparisons simpler, the values displayed for the lognormal distributions correspond to the mean and standard deviation of the distribution, not the actual *μ* and *σ* parameters (see Materials and methods).

Parameter	Homogeneous			Heterogeneous			Description
	E	I_1_	I_2_	E	I_1_	I_2_	
E_leak_[mV]	−76.43	−64.33	−61	N(-73,4)	N(-67.5,2)	N(-62.6,2)	resting membrane potential
V_thresh_[mV]	−44.45	−38.97	−34.44	N(-42,4)	N(-40,4)	N(-36,2)	spike threshold
V_reset_[mV]	−54.18	−57.47	−47.11	N(-52,5)	N(-58,6.4)	N(-54,5.4)	reset potential
G_leak_[nS]	4.64	9.75	4.61	N(4.73,0.38)	N(9.09,0.75)	N(4.5,0.2)	leak conductance
C_m_[pF]	116.52	104.52	102.87	N(114,8.7)	logN(68.9,35.6)	logN(82.24,17.7)	membrane capacitance
t_ref_[ms]	2.05	0.52	1.34	logN(1.8,0.25)	logN(0.5,0.01)	logN(1.3,0.05)	absolute refractory time
*τ*_m_[ms]	25.11	10.72	22.33	logN(22,2)	logN(9.5,2)	logN(20,2)	membrane time constant
R_m_[MΩ]	215.54	102.53	217.10	logN(160,50)	logN(100,10)	logN(210,10)	input resistance

Due to the nature of the chosen neuronal formalism (see *Neuronal dynamics*), some model parameters have no direct proxy with experimental measures and were instead determined considering their relations to other variables for which direct experimental data exists. For example, V_reset_ is not, strictly speaking, a biophysically meaningful variable; its value was chosen considering the data for afterhyperpolarization potentials E_AHP_ relative to the resting membrane potentials E_L_. Whenever such discrepancies occurred or when parameters had to be derived relative to others, we selected those values that better matched the experimental data. Overall, we observed a remarkable consistency in the ranges of parameter values considered across the different data sources.

#### Neuronal heterogeneity

**Fig 1:**
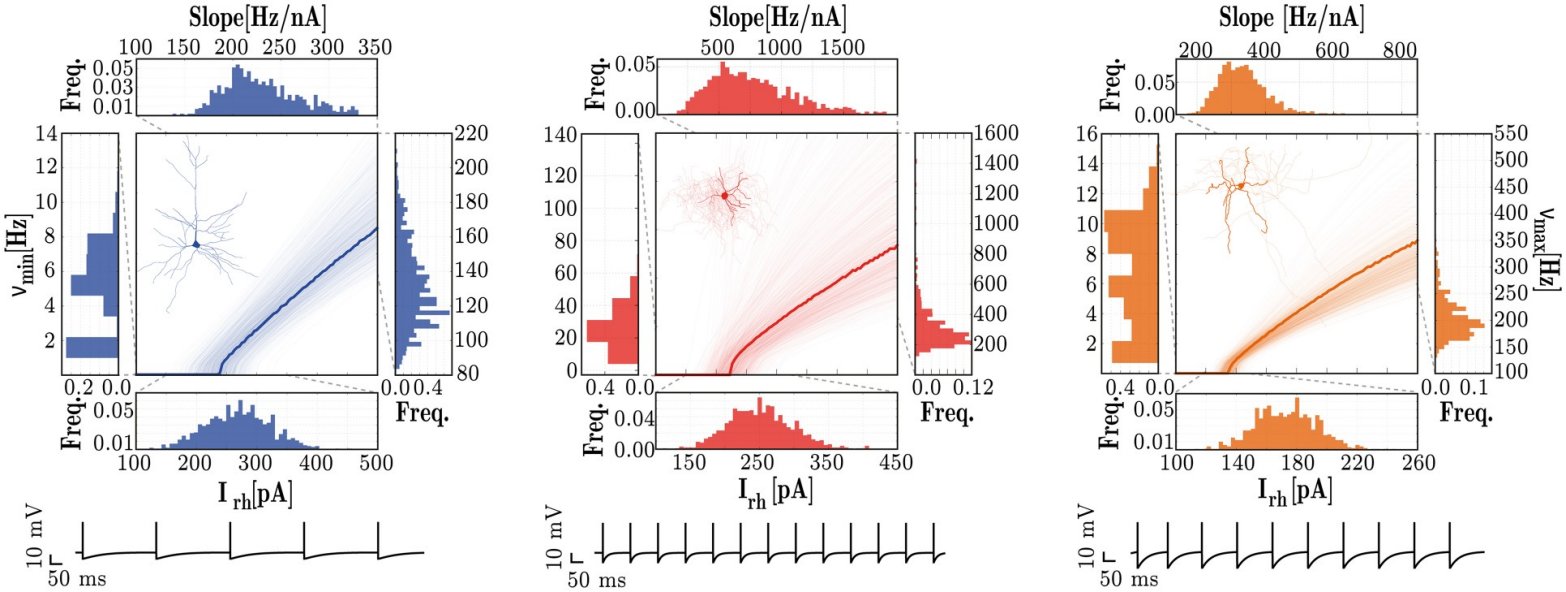
Response properties of the three different neuronal types, E (left), I_1_ (middle) and I_2_ (right). For each neuronal class, the central panels depict single neuron fI curves and the marginal panels give the corresponding distributions of the neuron’s rheobase currents (I_rh_[pA], bottom), minimum firing rates (*ν*_min_[*Hz*], left); maximum firing rates (*ν*_max_[*Hz*], right) and the slope of the fI curve (Slope[Hz/nA], top). The data was obtained from 1000 neurons of each class. The membrane potential traces depicted in the bottom correspond to the response of the homogeneous neurons (bold traces in the fI curves) to a stimulus step of amplitude I_rh_ + 10 pA, for a duration of 1 second.

In the homogeneous condition, the parameters for all neurons of a given class are fixed and chosen as a representative example of that class (see left side of Table 1 and bold fI curves in Fig 1). To incorporate neuronal heterogeneity, the specific values of each parameter are independently drawn from a probability distribution, specific to each neuron class (see right side of Table 1 and individual fI curves in Fig 1).

To ensure comparability among conditions, we tuned the intrinsic adaptation parameters (*a*, *b*, see *Neuronal dynamics*) independently for each neuron type, in order to retain the following relations among neuronal classes:

- Rheobase current (excitability)—*I*_rh_(E) ≈ *I*_rh_(I_1_) > *I*_rh_(*I*_2_)
- Slope of f-I curve (gain)—*g*(I_1_) > *g*(I_2_) > *g*(E)
- Minimum firing rate—*ν*_min_(I_1_)> *ν*_min_(I_2_) > *ν*_min_(E)
- Maximum firing rate—*ν*_max_(I_1_) > *ν*_max_(I_2_) > *ν*_max_(E)

This led to the following values (*a*, *b*) for sub-threshold and spike-triggered adaptation, respectively: E = (4, 30), I_1_ = (0, 0), I_2_ = (2, 10).

One of the advantages of using the modelling approach presented in this study is the possibility to directly interpret the biophysical meaning of the different parameters. In this case, these results highlight the fact that fast spiking interneurons (I_1_) do not exhibit any form of intrinsic adaptation, which is a reasonable result for a neuron whose primary role is to respond quickly and provide dense and fast, feed-forward inhibition [103]. It should be noted that, after fixing all the neuronal parameters as described above, the absolute values of these four properties did not exactly match the corresponding experimental reports. For example, the values obtained for the rheobase currents were, on average, larger than the ranges reported experimentally, for all 3 neuron classes (see S2 Table). These discrepancies in absolute values and their potential impact were ameliorated by ensuring the above relations between the key response properties of the different neuronal classes were retained.

#### Synaptic properties

**Fig 2:**
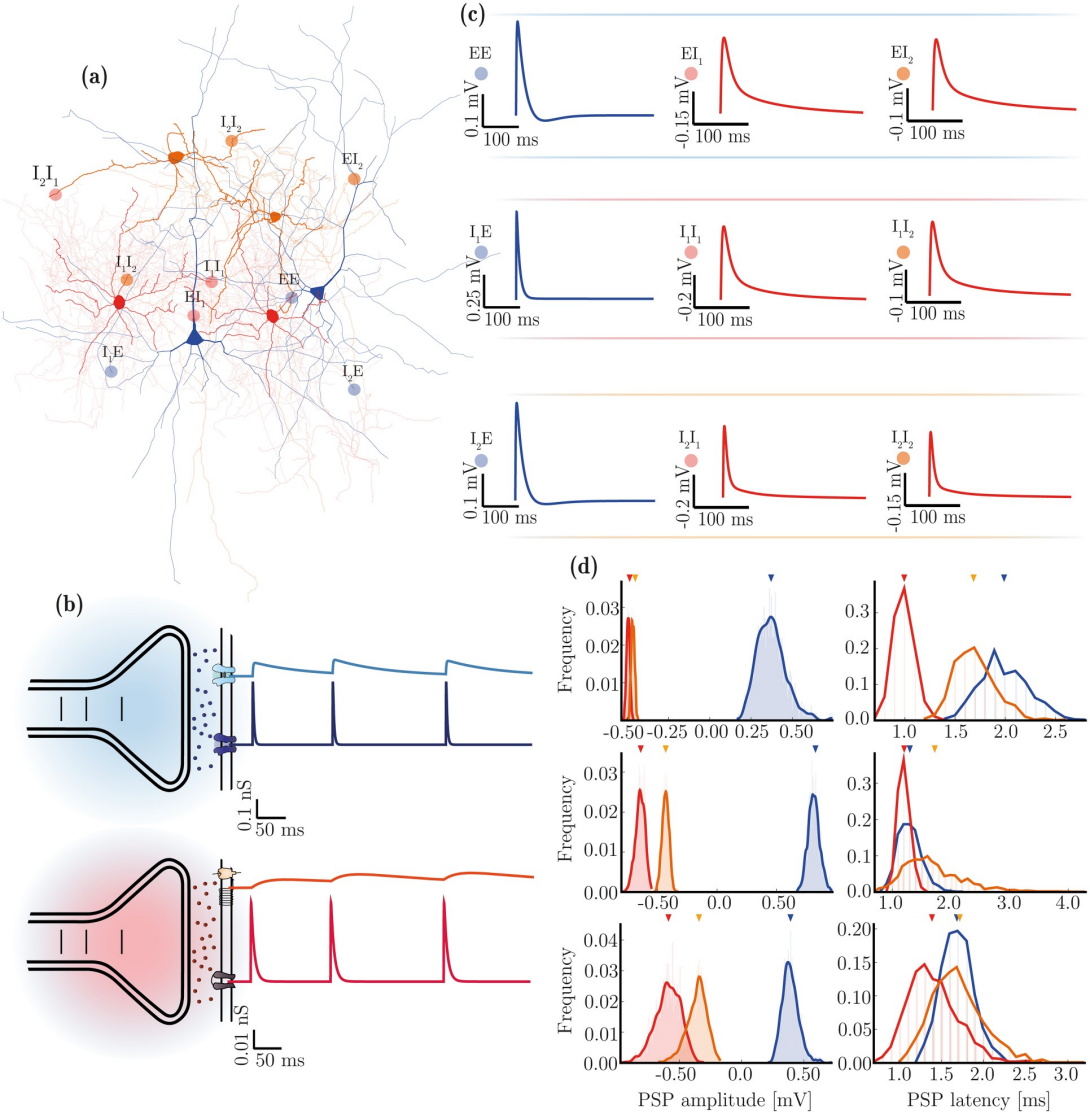
Diversity of synaptic transmission in the microcircuit. (**a**) Illustration of the three neuron and nine synapse types considered in this study. (**b**) Characteristics of synaptic transmission in excitatory (blue) and inhibitory (red) synapses, comprising fast and slow components. (**c**) Kinetics of spike-triggered PSPs dependent on pre- and post-synaptic neuron type and determined by the specific receptor kinetics and composition in the postsynaptic neuron. The depicted traces are the PSPs at rest in E neurons (top row) and at a fixed holding potential of −55 mV for inhibitory neurons (I_1_: middle row, I_2_: bottom row), as a function of receptor composition and correspond to the values reported in Table 2 (last column). (**d**) Distributions of PSP amplitudes and latencies after rescaling (by *w*^syn^ and *d*^syn^, respectively, see Table 3) in the homogeneous (top arrows) and heterogeneous (distributions) conditions, for synapses onto E (top), I_1_ (middle) and I_2_ (bottom) neurons. Note that the latency distributions are discrete in that for technical reasons, they can only assume values that are a multiple of the simulation resolution.

With three neuronal populations, as described above, there are nine possible types of synaptic connection, i.e. syn ∈ {EE, EI_1_, EI_2_, I_1_E, I_2_E, I_1_I_1_, I_1_I_2_, I_2_I_1_, I_2_I_2_}. Synapse types can be grouped by transmitter composition and/or post-synaptic effect, as excitatory syn_E_ = {EE, I_1_E, I_2_E} and inhibitory syn_I_ = {EI_1_, EI_2_, I_1_ I_1_, I_1_ I_2_, I_2_ I_1_, I_2_ I_2_}, as illustrated in Fig 2a. For simplicity, we consider all synaptic transmission as being mediated by either glutamate (excitatory synapses) or gamma-aminobutyric acid (GABA, inhibitory synapses), as illustrated in Fig 2b. This is a reasonable simplification given that these are, by far, the primary neurotransmitters used in the neocortex, as demonstrated by immunohistochemistry [104] and receptor autoradiography studies [39, 105, 106]. Additionally, this is a common assumption underlying the great majority of theoretical and computational studies.

To accommodate the wealth of data available regarding the phenomenology of synaptic transmission and to provide a significant step forward from the traditional approaches, we chose a relatively complex biophysical model [99, 107–109]; primarily due to its plausibility but also due to the availability of direct parameters in the experimental literature (e.g. [109]). The model, described fully in the Materials and Methods, captures the detailed kinetics of single receptor conductances (Fig 2b).

The use of this model allows us to specify different receptor parameters depending on the neuron type they are expressed in (see Table 2), in order to directly match empirical data on receptor kinetics and relative conductance ratios in different neuronal classes. In the absence of such direct data for specific synapses (particularly those involving I_2_ neurons), we tuned these parameters to match the resulting PSP/PSC kinetics, as well as the relative ratios of total charge elicited by the receptors that compose such synapses. For a detailed list of the data sources used to constrain these parameters, consult S1 Appendix.

**Table 2:** Differential receptor expression in the different neuron types. The kinetics and relative conductance of the different receptors that make up an inhibitory or excitatory synapse onto each neuron results in post-synaptic potentials with equally discernible kinetics. The parameters were chosen based on the corresponding receptor conductance data, if directly available and/or on the characteristics of the resulting PSPs, resulting in a substantial diversity of postsynaptic responses (see Fig 2c). (*) The PSP values reported in this table were obtained by fitting a double exponential function to single, spike-triggered PSPs, recorded at rest for E neurons and at a fixed holding potential of −55 mV for I neurons.

Neuron Type	Receptors	Conductance parameters						Resulting PSPs (*)		
		$\bar{g}\left[\;\text{nS}\right]$	*E*[mV]	*τ*_rise_[ms]	*r*	$\tau_{{\text{decay}}_{f}}\left[\;\text{ms}\right]$	$\tau_{{\text{decay}}_{s}}\left[\;\text{ms}\right]$	J_syn_[mV]	*τ*_rise_[ms]	*τ*_decay_[ms]
E	AMPA	0.9	0	0.3	1	2	-	0.82	3.16	17.8
	NMDA	0.14	0	1	0	-	100			
	GABA_A_	0.15	−75	0.25	1	6	-	−0.1	3.2	64.3
	GABA_B_	0.009	−90	30	0.8	200	600			
I_1_	AMPA	1.6	0	0.1	1	0.7	-	0.5	0.8	10.7
	NMDA	0.003	0	1	0	-	100			
	GABA_A_	1	−75	0.1	1	2.5	-	−0.25	1.65	17.5
	GABA_B_	0.022	−90	25	0.8	50	400			
I_2_	AMPA	0.8	0	0.2	1	1.8	-	0.6	2.4	17.3
	NMDA	0.012	0	1	0	-	100			
	GABA_A_	0.7	−75	0.2	1	5	-	−0.6	3.2	42
	GABA_B_	0.025	−90	25	0.8	150	500			

Having fixed the kinetics of post-synaptic responses according to neuron class (Table 2), we finally rescale the PSP amplitudes (*w*^syn^) and latencies (*d*^syn^) independently for each synapse type (see below), in order to account for the effects of different presynaptic neuron classes and to explicitly match the data reported in [93]. As a result of this parameter fitting process, the responses generated by the synaptic model are good matches to the responses experimentally observed in the nine types of biological synapses represented in this study.

#### Synaptic heterogeneity

As all the receptor parameters are fixed and neuron-specific (Table 2), we introduce synaptic heterogeneity by simply distributing the individual values of weights and delays (Fig 2d and Table 3). Whereas in the homogeneous condition, synaptic efficacies (*w*^syn^) and conduction delays (*d*^syn^) are fixed and equal for all connections of a given type, in the heterogeneous condition, these values are randomly drawn from lognormal distributions, left-truncated at 0 for weight distributions and 0.1 for delay distributions, and parameterized such that the distributions’ means are equal to the homogeneous value. For consistency among the various data sources, we fix the connectivity parameters, including not only structural aspects, but also synaptic weights and delays, to match the data reported in [93]. It is worth noting that variability in delay distributions could also be considered structural since they are primarily determined by morphological features (axonal length). However, the synaptic delays were chosen to match the observed latencies in postsynaptic responses. So, for convenience and simplicity, we treat variations in synaptic weights and delays as synaptic properties.

**Table 3:** Synaptic and structural parameters in the microcircuit. Each of the nine connection types (which can be grouped as indicated) is characterized by a specific connection density, weight and delay. In the homogeneous condition, weights and delays are fixed and equal to the mean values ($\mu_{w}^{\;\text{syn}}$, $\mu_{d}^{\;\text{syn}}$) for all synapses of a given type, whereas in the heterogeneous condition they are independently drawn from lognormal distributions with the corresponding mean and standard deviation. The last two rows in the table are the connection-specific structural bias parameters, used to skew the network’s degree and weight distributions. The indicated values were taken directly from [110] and [71]. The cases marked with—or x, correspond to connections that were either tested, revealing no significant effect (-) or untested due to missing data (x). In both cases, we set the corresponding values to 0.

Parameter	Connection Types									Description
	syn_*E*_			syn_*I*_						
	EE	IE		EI		II				
	E → E	E → I_1_	E → I_2_	I_1_ → E	I_2_ → E	I_1_ → I_1_	I_1_ → I_2_	I_2_ → I_1_	I_2_ → I_2_	
*p*^syn^[%]	16.8	57.5	24.4	60	46.5	55	24.1	37.9	38.1	Connection density
$\mu_{w}^{\;\text{syn}}$	0.45	1.65	0.638	5.148	4.85	2.22	1.4	1.47	0.83	Synaptic weights
$\sigma_{w}^{\;\text{syn}}$	0.10	0.10	0.11	0.11	0.11	0.14	0.25	0.10	0.2	
$\mu_{d}^{\;\text{syn}}$	1.8	1.2	1.5	0.8	1.5	1	1.2	1.5	1.5	Synaptic delays
$\sigma_{d}^{\;\text{syn}}$	0.25	0.2	0.2	0.1	0.2	0.1	0.3	0.5	0.3	
$k_{\text{in}}^{\;\text{syn}}$	5	5	0	-	0	-	-	x	x	Degree distribution bias
$k_{\text{out}}^{\;\text{syn}}$	5	0	0	-	-	0	-	x	x	
$c_{\text{in}}^{\;\text{syn}}$	1	1	1	-	x	-	-	x	x	Weight correlation bias
$c_{\text{out}}^{\;\text{syn}}$	0	1	0	-	x	0	-	x	x	

The mean weight values were chosen to rescale the PSP amplitude of each synapse type to the target value. Additionally, as described below, if both structural and synaptic heterogeneity conditions are considered simultaneously, the weight distributions are skewed in order to introduce structural weight correlations (see *Generating structural heterogeneity* in Materials and methods).

#### Structural properties

The structure of the network is defined by the density parameter *p*^syn^, which is specific for each of the nine connection types. For consistency with the results and methods presented in [110] and [71], we set the connection densities (*p*^syn^) to the values reported in [93] (see Table 3). However, it is worth noting that the values reported in the literature can vary substantially, possibly reflecting methodological/technical limitations and/or the fact that connection density is a highly region- / species-specific feature. In fact, among all the complex parameter sets used throughout this study, the single parameter that was most difficult to reconcile across multiple sources was *p*^syn^. In the homogeneous case, random connections are created between neurons in a source population *pre* and target population *post* (with *pre*, *post* ∈ {E, I_1_, I_2_}) with a probability given by *p*^syn^.

#### Structural heterogeneity

In order to account for structural heterogeneity, we bias the network’s degree distributions by modifying the structure of the connectivity matrix *A*^syn^, following the methods introduced in [71, 110] and validated against the same primary sources of experimental data used in this study.

**Fig 3:**
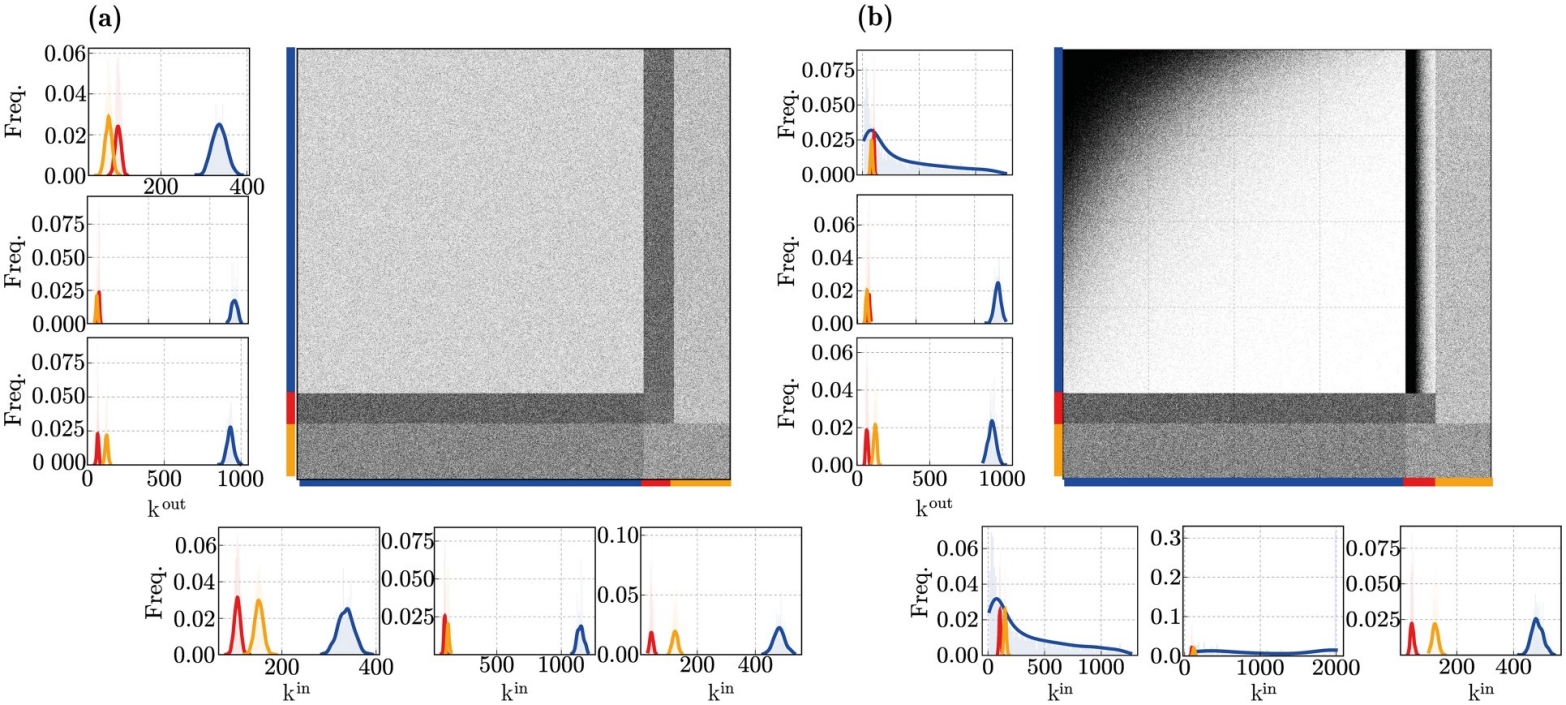
Microcircuit connectivity for (a) homogeneous and (b) heterogeneous network structures. The large panels show the circuit’s complete connectivity matrix *A*^syn^, comprising all connections among all neuron classes. The panels on the left sides show the corresponding out-degree (k^out^) distributions for the neuronal populations (E, I_1_ and I_2_ from top to bottom); the panels at the bottom show the corresponding in-degree (k^in^) distributions (E, I_1_ and I_2_ from left to right).

By controlling the skewness of the out-/in-degree distributions (k^out/in^ parameters, see *Generating structural heterogeneity* in Materials and methods), we can generate completely random, uniform connectivity (k^out/in^ = 0, Fig 3a) or highly structured in-/out-degree distributions, with a larger variance in the number of connections per neuron (k^out/in^ > 0, Fig 3b). For the structural heterogeneity condition, these parameters were fixed to the values that were shown to provide a better fit for the experimentally determined connectivity data (see [71, 110] and Table 3).

Additionally, in conditions where synaptic heterogeneity is also present (fully heterogeneous circuit), structural heterogeneity is further expressed as a bias in the synaptic efficacies for all incoming and outgoing connections to a given neuron. Following [71, 110] and [72], this bias is implemented by rescaling individual synaptic efficacies in order to introduce correlations between them, see Table 3 and Materials and methods. It should be noted that structural heterogeneity only modified connections from E neurons as most of the other connections were shown to have negligible effects (marked with—in Table 3) or were not successfully tested (marked with x), due to technical constraints. Even though this is likely to be incomplete, it appears to be sufficient to capture the most significant structural effects and their impact in population dynamics, while explicitly accounting for the experimental data in [93]. So, for consistency, we implemented and parameterized structural heterogeneity in the same manner as that reported in [71] and [110].

### Emergent population dynamics

**Fig 4:**
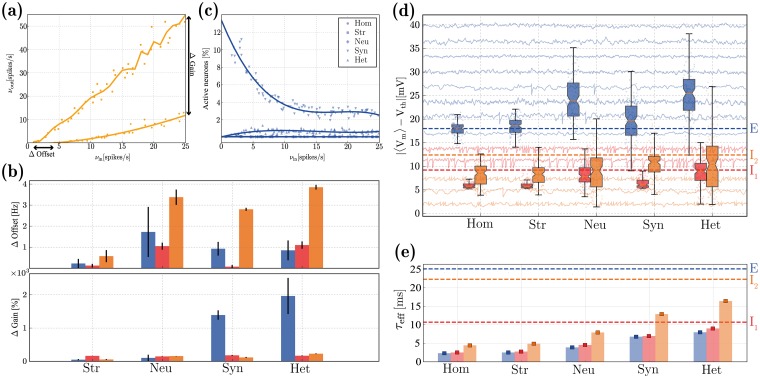
Characteristics of population activity in the *quiet* state. (**a**): Rate transfer function of the I_2_ neurons in the homogeneous (dots) and structurally heterogeneous (squares) conditions. (**b**): Change in absolute gain (ΔGain, expressed as a percentage of rate gain relative to the baseline homogeneous condition) and offset (ΔOffset), see annotations in (a), for the E (blue), I_1_ (red) and I_2_ (orange) populations, for each heterogeneous condition (structural, neuronal, synaptic, and all forms combined), relative to the homogeneous condition. Error bars indicate the standard deviation across ten simulations per condition. (**c**): Fraction of active E neurons in the different conditions as a function of input rate *ν*_*in*_. (**d**): Distributions of absolute distances between neurons’ mean membrane potentials 〈V_m_〉 and their firing threshold V_th_, for the three neuronal populations considered, under the different heterogeneity conditions. The dashed lines indicate the corresponding values reported in [94]. For illustrative purposes, we depict *V*_*m*_ traces for a small set of randomly chosen neurons in the background. (**e**): Effective membrane time constants (*τ*_eff_ = C_m_/〈G_total_〉) in the noise-driven regime, for the different neuronal classes and conditions. Error bars correspond to the means and standard deviations across each population. The dashed lines indicate the baseline values (*τ*_0_ = C_m_/g_leak_). All results depicted were calculated over an observation period of 10 *s*. The results depicted in (**d, e**) were acquired with a fixed input rate of *ν*_*in*_ = 10 spikes/s and correspond to the distributions across the population, for a single simulation per condition.

Throughout this study, and in order to isolate the effects of different sources of heterogeneity, we consider five different microcircuits: fully homogeneous (Hom), structural (Str), neuronal (Neu) or synaptic (Syn) heterogeneity in isolation and a fully heterogeneous circuit (Het), accounting for the combined effects. In this section, we set out to quantify and evaluate the specific impact of the different forms of heterogeneity on the characteristics of population activity. To do so, we consider the circuit’s responses to an unspecific and stochastic external input, modelling cortical background / ongoing activity (see *Input specifications* in Materials and methods). We determine and compare the circuit’s responsiveness by looking at the population rate transfer functions, as exemplified in Fig 4a for I_2_ neurons (complete results are provided in S1 Fig), and summarize the results by the change in absolute gain (ΔGain) and offset (ΔOffset) introduced by each source of heterogeneity, relative to the homogeneous condition (Fig 4b).

All heterogeneous conditions, particularly neuronal and synaptic, cause a slight offset for all neuron types (more significant for I_2_ neurons), making them more responsive (firing at lower input rates) but the effect is not substantial (Fig 4b, top). In most of the conditions analysed, the E population is rather unresponsive, with less than 1% of the neurons active (Fig 4c) and firing at rates inferior to 1 spikes/s, regardless of the input rate. While structural and neuronal heterogeneity are incapable of circumventing this effect, synaptic heterogeneity appears to be important for the network to fire at more reasonable rates (albeit, still very sparsely), resulting in a substantial modulation of the gain of the rate transfer function (Fig 4b, bottom).

It should be noted that the impact of structural heterogeneity alone is mitigated by the low E rates, since the structural bias exists only within excitatory synapses or between excitatory neurons and fast-spiking interneurons (i.e. E E, I_1_ E, see Table 3). So, if the E population rarely fires, it is difficult to ascertain the effects of structural heterogeneity, suggesting either that its relevance pertains mostly to active states, when population activity is slightly higher, or that it is negligible at this scale.

The extremely sparse firing of E neurons that we observe is consistent with physiological measurements in layer 2/3 (e.g. [92, 94, 111–114]), but it significantly limits the degree to which we can quantify the effects of heterogeneity on population activity. So, in order to obtain a greater insight, we look at the sub-threshold responses and characteristics of membrane potential dynamics (Fig 4d and 4e). Excitatory neurons are always significantly hyperpolarized, with their mean membrane potentials kept far from threshold (Fig 4d, blue) and thus require much stronger depolarizing inputs to fire, compared with both inhibitory types. The inhibitory populations are, on average, much more depolarized and their membrane potentials fluctuate closer to their firing thresholds, particularly I_1_ (Fig 4d, red). Qualitatively, the ratio of average degree of depolarization among the different populations is retained across all conditions, with I_1_ neurons being strongly depolarized, followed by I_2_ and E and is consistent with experimental reports for circuits in a state of *quiet wakefulness* (Fig 4d, dashed lines). This feature stems directly from the electrophysiological properties of the different neuronal classes and the interactions among the 3 populations (given that it is already observed in the homogeneous circuit). Both synaptic and neuronal heterogeneity greatly increase the variability in the distribution of mean membrane potentials across all the neurons and cause a slight overlap between E and I_2_ populations, an effect that is also consistent with experimental evidence [94].

Active synapses contribute to the total membrane conductance and cause a deviation from the resting membrane time constant [115, 116]. This *shunting* effect may be mild in sparsely active circuits [117], but it provides a form of activity-dependent modulation of single neurons’ integrative properties [118], which constrain the circuit’s responsiveness. In the absence of synaptic input, I_1_ neurons have faster responses, characterized by a short baseline membrane time constant (*τ*_0_ = C_m_/g_leak_ ≈ 10.7 ms), whereas I_2_ and E neurons are slower (*τ*_0_ ≈ 22.3 and 25.1 ms, respectively) and can thus integrate their synaptic inputs over a larger time scale (dashed lines in Fig 4e). This relationship between the neuronal classes (*τ*_eff_(I_1_) < *τ*_eff_(I_2_) < *τ*_eff_(E)) is a consequence of the neurons’ physiological properties and is consistent with empirical evidence [59, 118, 119]. However, when driven by external input, the ratio is modified and I_2_ neurons respond slowest, i.e. *τ*_eff_(I_2_) > *τ*_eff_(I_1_) > *τ*_eff_ (E). The presence of heterogeneous synapses is important to ameliorate the magnitude of this *shunting* effect (Fig 4e), which is very substantial in all conditions. It should be noted that, while the sparsity of recurrent activity (particularly that of E neurons), would prompt us to expect a very minor reduction in *τ*_eff_, the observed results are caused by the large synaptic input provided as background.

#### Excitation/inhibition balance

The balance of excitation and inhibition is one of the most important and widely observed features in the neocortex. It plays a pervasive role in modulating and stabilizing circuit dynamics [120], shifting the population state [121–123], selectively gating and routing signals [124–126] and maintaining sparse, distributed dynamics [114, 117], critical for adequate processing and computation [127–129].

**Fig 5:**
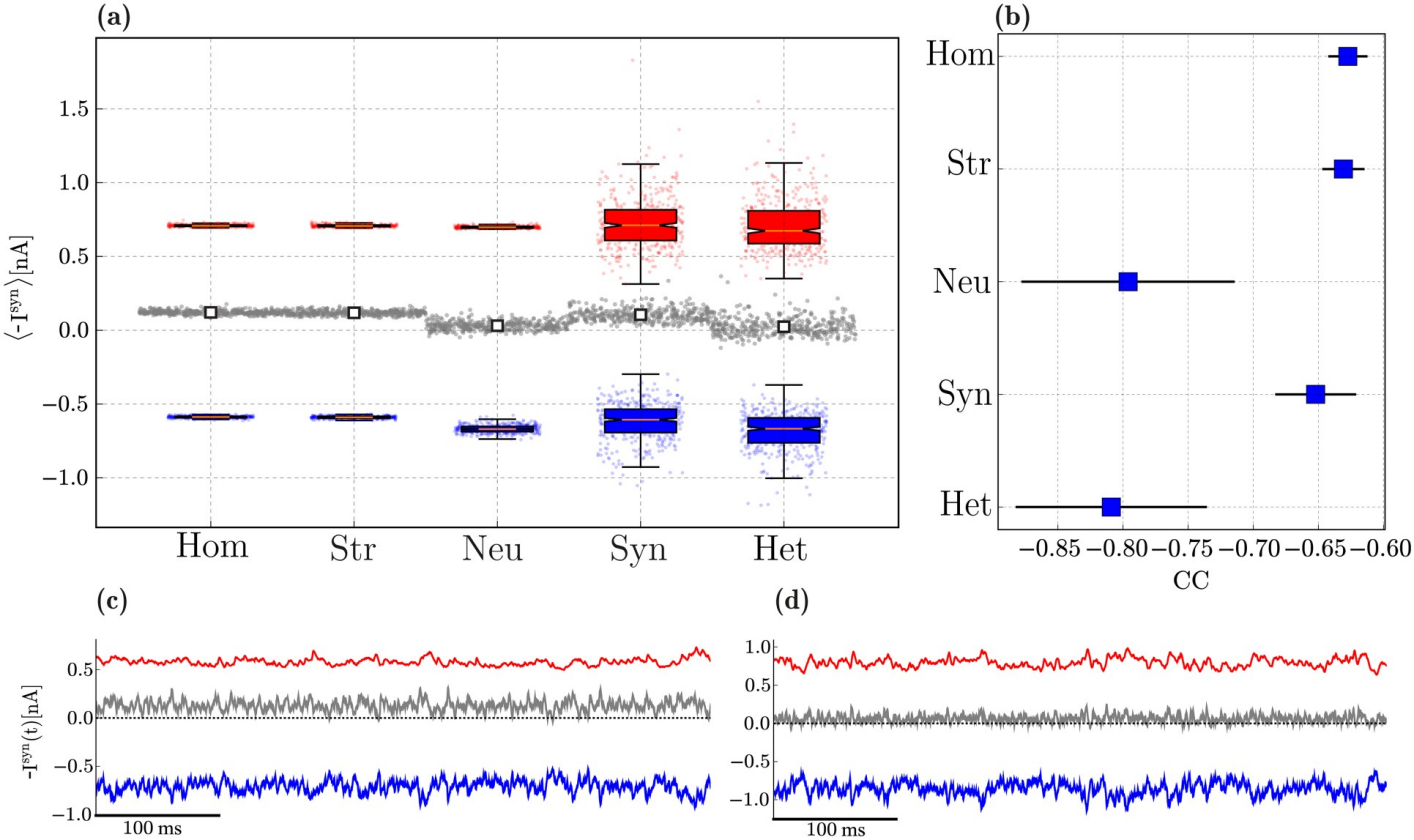
Balance of excitation and inhibition in excitatory neurons driven by background, Poissonian input at *ν*_*in*_ = 10 spikes/s. (**a**): Distribution of mean amplitudes of excitatory (blue) and inhibitory (red) membrane currents onto E neurons in the different conditions, as well as the absolute difference between them (grey). For each condition, we show the single data points, where the currents onto a given neuron are summarized as a set of 3 points (〈I^exc^〉 in blue, 〈I^inh^〉 in red and |〈I^inh^〉 − 〈I^exc^〉| in grey). Overlaid on top of these data points are the distributions across all the neurons, summarized as box-plots: the box represents the first and third quartiles (IQR); the median is marked in red; the whiskers are placed at 1.5 IQR and the outliers can be seen in the underlying data points. The white markers in the middle display the mean difference of synaptic amplitudes across all neurons, for each condition. (**b**): zero-lag correlation coefficient between excitatory and inhibitory synaptic currents (mean and standard deviation across the E population). (**c, d**): Examples of the total excitatory and inhibitory synaptic currents received by a randomly chosen E neuron in the homogeneous and fully heterogeneous conditions, respectively. Results in (**a**) and (**b**) were gathered from 10 simulations per condition.

As demonstrated in the previous section (Fig 4), the different sources of heterogeneity significantly influence the circuit’s responsiveness, partially by modifying how the different neurons and neuronal populations receive and integrate their synaptic inputs. These differences can also be observed in the amplitude and time course of the total excitatory and inhibitory drive onto each neuron, as can be seen in Fig 5. The results shown are a compound effect of the kinetics of the specific receptors involved and the post-synaptic currents they elicit, the physiological properties of the different neuronal classes as well as the density of specific connections.

In a globally balanced state, the amplitudes of excitatory and inhibitory synaptic currents cancel each other on average. This occurs in our microcircuit model only in the presence of neuronal heterogeneity (Fig 5a). Variability in connectivity structure is indistinguishable from the homogeneous condition, whereas variability in synaptic weights and delays significantly increases the variance in the distribution of post-synaptic current amplitudes, but does not shift the mean. This results in an inhibition-dominated synaptic input, resembling that of the homogeneous condition (see also Fig 5c), despite the substantially different distributions.

Apart from being balanced on average, a condition of “detailed” balance [126, 130] is characterized by E and I currents that closely track each other and are strongly anti-correlated (Fig 5b). In the homogeneous circuit, excitatory and inhibitory currents are most weakly anti-correlated (*CC* ≈ −0.63, see also Fig 5c). Synaptic heterogeneity causes a slight improvement, but the most important contribution to this effect comes from neuronal heterogeneity. In this condition, the mean correlation coefficient reaches *CC* ≈ −0.8, although it also introduces a greater variance than synaptic or structural heterogeneity (see also Fig 5d).

Both global and detailed balance thus appear to be emergent properties of heterogeneous microcircuits, primarily due to neuronal diversity, but encompassing also a clear influence of synaptic diversity. As is the case with all results presented so far, the fully heterogeneous circuit retains several key properties of interest, but appears to inherit them from different sources. As before, the effects of structural heterogeneity are mitigated by the very sparse firing of the E population, which render its effects moot and no significant deviation from the homogeneous condition is observed.

### Active processing and computation

In order to induce a functional state, engaging the circuit in active processing, we introduce an additional input signal, directly encoded as a piece-wise constant somatic current (see *Input specifications* in Materials and methods). We began by tuning the input amplitudes (of both background input firing rate *ν*_in_ and external input current *ρ*_*u*_) independently, for each condition, in order to approximate the relative ratio of mean firing rates among the different populations (see S2 Fig), i.e. we attempt to find a combination of input parameters that allows the mean firing rates to remain within realistic bounds (*ν*_E_ ∈ [0.5, 5], $\nu_{\;I_{1}}\in \left[10,25\right]$, $\nu_{\;I_{2}}\in \left[3,15\right]$, considering the values reported in [93, 94, 111, 114]).

We consider the circuit’s responses to this input signal as an *active* state, as opposed to the condition explored in the previous sections, where the circuit was driven solely by background, stochastic input (noise). It is worth noting, however, that the similarities between what we call *quiet* and *active* states and their biological counterparts are limited (see Discussion). In the following, we show that despite these limitations, the actively engaged circuit operates in similar dynamic regimes to its biological counterpart and maintains the key statistical features that are most likely to play a significant role in modulating the circuit’s processing capacity.

In this section, we assess the microcircuit’s capacity to compute complex functions of the input signal, as described in the Materials and Methods. Note that we purposefully removed any predetermined structure in the input signal, such that the measurements reflect the properties of the system and not the acquisition of structural information in the input. If we were to consider naturalistic sensory input as the driving signal, this would not be the case. Furthermore, we intentionally focus on generic information processing as the ability the perform arbitrary transformations on an input signal and not on specific functions which might be performed by specific microcircuits.

#### Spiking activity in the *active* state

**Fig 6:**
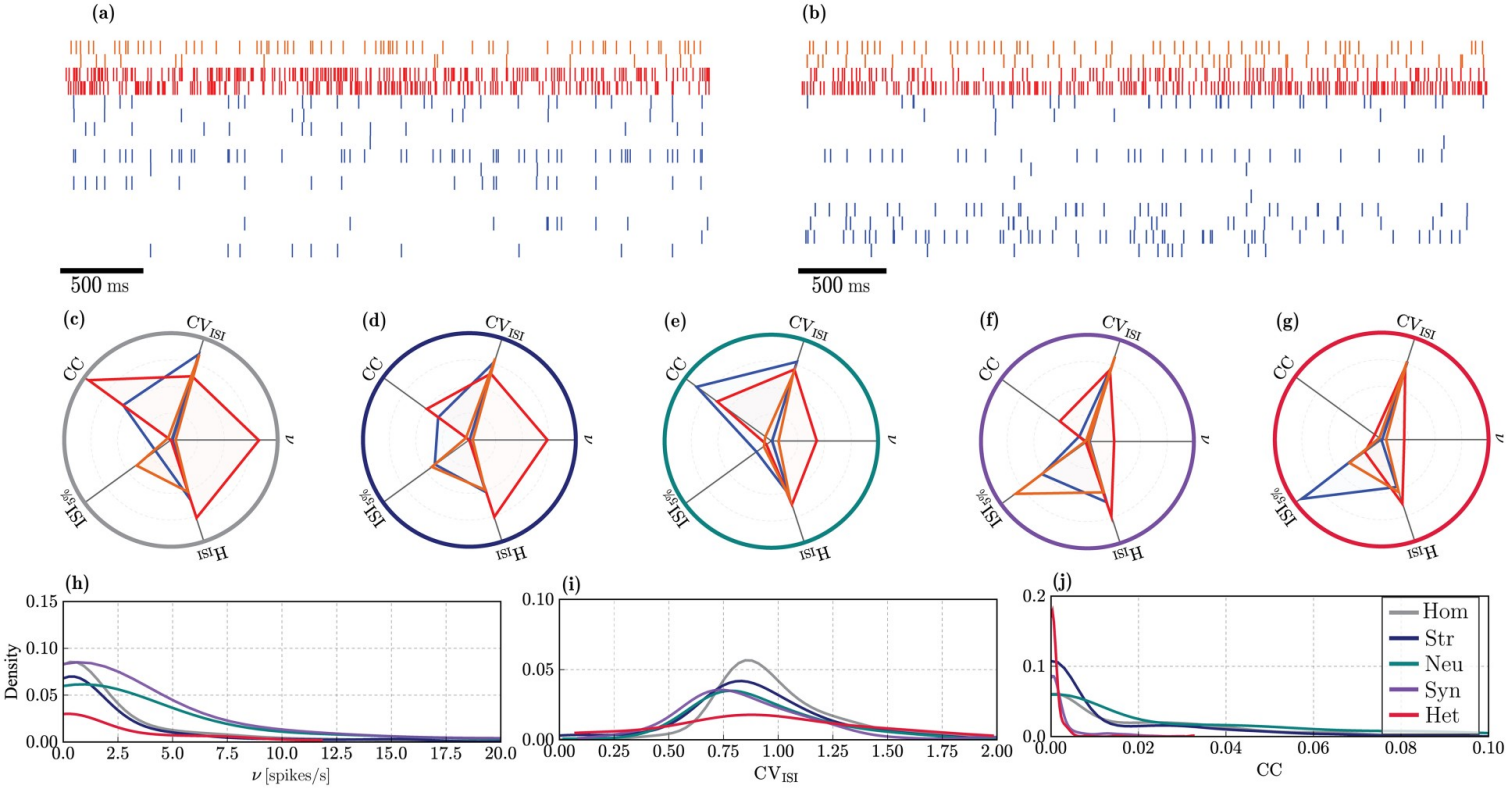
Statistical properties of spiking activity in the active state for the E (blue), I_1_ (red) and I_2_ (orange) populations. (**a, b**): Example raster plots depicting the activity of a small, randomly chosen subset of neurons, over a recording period of 2.5s in a homogeneous and fully heterogeneous circuit, respectively. (**c**)-(**g**): Activity statistics profiles for the different populations (overlaid) in the different conditions. The radial axes represent: mean pairwise correlation coefficient (CC), coefficient of variation of the inter-spike intervals (CV_ISI_), mean firing rate (*ν*[spikes/s]), entropy (H_ISI_[bits]) and burstiness (ISI_5%_[s]) of the firing patterns. The values along the radial axes are normalized to the largest value across all neuron classes in each condition. The units have been removed for better legibility (see S3 Fig). (**h**)-(**j**): Distribution of firing rates *ν*[spikes/s], CV_ISI_ and CC for the E population in the different heterogeneity conditions (see legend in (**j**)). The results depicted are population averages, obtained from one simulation per condition.

Due to the extremely sparse firing observed in the quiet state, an adequate comparison of spiking statistics is only sensible in the active condition. While no explicit effort was taken to constrain the circuit’s operating point when tuning the input parameters (the focus was purely on average firing rates), all conditions operate on an asynchronous irregular regime (exemplified by the raster plots in Fig 6a). This regime is characterized by low pairwise correlations (CC < 0.03) and high coefficients of variation (CV_ISI_ ≈ 0.9), for all neuron classes, in all conditions. Each condition generates different spiking responses with slight variations in activity statistics. The profiles of the activity statistics for the three neuronal classes are summarized in Fig 6c–6g for the five different conditions. The complete results, displaying the specific profiles for the different classes and conditions separately can be consulted in S3 Fig.

In the homogeneous condition (Fig 6a and 6c), I_1_ neurons exhibit the most distinctive profile, with the highest amount of synchrony and the largest firing rates as well as a very bursty and regular firing relative to any other neuronal class and any other condition. Consistent with empirical observations [94, 114, 131, 132], E neurons retain an extremely low firing rate (*ν*_E_ ≤ 1 spikes/s), even when stimulated by the extra input current that characterizes the active state. The main difference is that a larger fraction of the population is engaged and actively firing. This result demonstrates that sparse firing of E neurons is a stable characteristic of layer 2/3 microcircuits, emerging from the strong and dense inhibition provided primarily by I_1_ neurons that, firing at very high rates, strongly inhibit the E population, regardless of the variations introduced by different sources of heterogeneity and the addition of the extra excitatory input.

The impact of the various heterogeneity conditions particularly affects the degree of synchronization and burstiness across the different populations. Only the firing rates of I_1_ neurons are significantly modified by heterogeneity, whereas for all other neuron classes, they remain consistently low. Irregularity and randomness in the firing patterns are mostly unaffected as is clear by observing the similarity in the respective axes (H_ISI_ and CV_ISI_ in Fig 6c–6g).

The effects of structural heterogeneity (Fig 6d) are only noticeable on the neuronal classes that are directly affected (E and I_1_, see Table 3); no changes in activity statistics are observable for the I_2_ population (orange profiles in Fig 6c and 6d). Excitatory neurons fire less synchronously and exhibit a much lower tendency to fire in short spike bursts, compared with the homogeneous condition. On the other hand, I_1_ neurons show a slight decrease in synchrony and firing rate.

Diversity in neuronal parameters (Fig 6e) strongly affects the response properties of I_2_ neurons, slightly increasing their firing rates and correlation coefficient. The most noticeable effect, however is a greatly increased tendency to fire in short bursts (ISI_5%_ ≈ 1*s*) which is the most significant deviation of the standard profile exhibited by this neuronal class in all other conditions (for a more complete comparison, consult S3 Fig). Heterogeneous E neurons have a higher tendency to fire synchronously (albeit still with very low CC), compared to any other condition. As for the I_1_ population, the most significant effect of neuronal heterogeneity is a reduction of the mean firing rate.

Synaptic heterogeneity (Fig 6f) causes a clear alteration of the firing profile of all neuron classes, particularly E and I_1_, resulting in a noticeable decrease in synchronization in all populations, thus having a marked decorrelating effect. It also produces a substantial reduction in the tendency for burst spiking in the E and I_2_ populations. The firing rates of both inhibitory populations are reduced (due to the chosen input parameters, see S2 Fig) which, consequently, leads to a slight increase in the E neurons’ firing rates that also fire slightly more regularly.

Interestingly, the firing profile observed in the fully heterogeneous circuit (Fig 6b and 6g) exhibits some unique features, different from any of those created by the individual sources of heterogeneity in isolation. Particularly prominent is the complete abolishment of any degree of synchronization in any of the neuronal populations, which show the smallest correlation coefficients of all the cases considered. This effect is likely primarily acquired from synaptic heterogeneity, but goes further than the effect observed there. The firing profile of I_2_ neurons in the fully heterogeneous circuit retains all the features observed in the homogeneous circuit, indicating that the variations introduced by neuronal and synaptic heterogeneity are counteracted by the complex interactions between the different sources of heterogeneity.

Overall, the statistics of population activity clearly demonstrate that the fully heterogeneous circuit is more than the sum of its parts, i.e. the variations introduced by the combination of multiple sources of heterogeneity cannot be fully accounted for by their individual effects and lead to more complex interactions that strongly modulate the circuit’s operating point. In addition, all heterogeneity conditions give rise to similar distributions (Fig 6h–6j), i.e. lognormal distributions of firing rates (argued to be a beneficial feature [72, 133]) and correlation coefficients as well as a Gaussian distribution of CV_ISI_, with mean close to 1. The different conditions simply modulate the parameters of the distributions: synaptic and neuronal heterogeneity broaden the firing rate distributions; synaptic heterogeneity alone is responsible for skewing the CCs to smaller values, an effect that is stronger and more pronounced in the fully heterogeneous circuit. It should be noted that the tails of the rate distributions in networks with heterogeneous neurons and/or synapses fall beyond the range typically observed in layer 2/3 microcircuits. The extra somatic current with which we emulate the active state drives the targeted sub-population to fire excessively in these conditions, which highlights a limitation of our approach (see *Limitations and future work*).

#### Temporal tuning and memory capacity

Online processing requires the continuous acquisition and integration of temporally extended information arriving through multiple time-varying input streams. As such, cortical microcircuits need to retain information over time (fading memory) and combine it in meaningful ways. This generic operating principle thus constitutes an important feature underlying cortical information processing (see e.g. [4, 134–136]), and is primarily determined by architectural constraints that potentially modulate the circuit’s operating timescales. In this section, we investigate the effect of heterogeneity on the ability of our microcircuit model to retain information over long timescales, enabling it to operate over a broad dynamic range.

**Fig 7:**
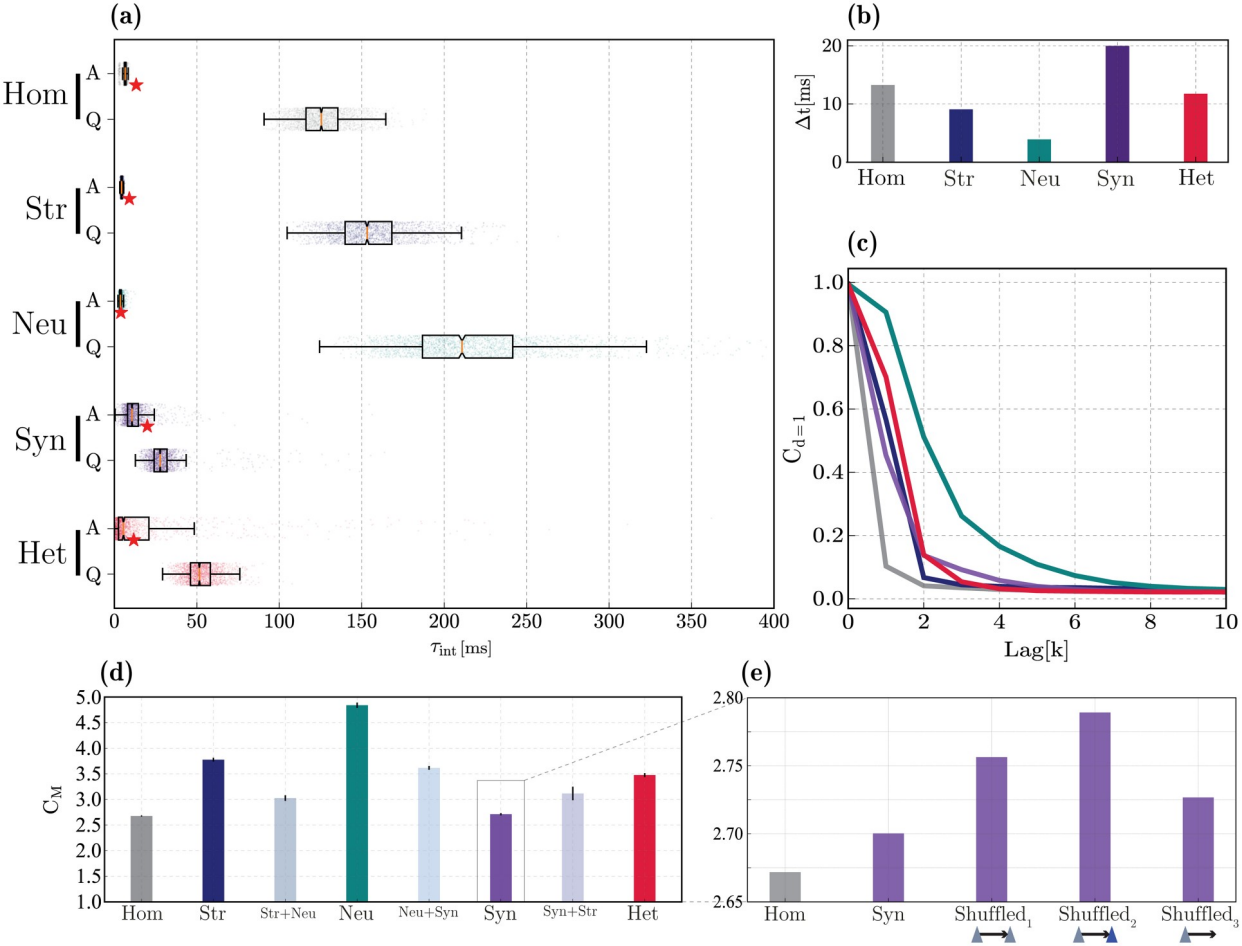
Temporal tuning and linear memory capacity. (**a**): distributions of intrinsic timescales (*τ*_int_) in the quiet (Q, bottom) and active (A, top) states, for each of the different conditions. (**b**) Optimal stimulus resolution (Δ*t*) that allows each circuit to perfectly track its input signal (see also red star markers in (**a**) and S4 Fig). (**c**): Fading memory functions for the different heterogeneity conditions, determined as the ability to reconstruct the input signal at different delays (k). Colours as in panel (**d**) below. (**d**): Total memory capacity, corresponding to the area under the curves in (**c**). Apart from the main conditions depicted in (**c**), pair-wise combinations among conditions are depicted in between. (**e**) Effects of synaptic heterogeneity on memory capacity, in conditions where the weight distributions are fixed, but re-shuffled such that the strongest weights are assigned to the connections among the input population (Shuffled_1_), from the input population to all excitatory neurons (Shuffled_2_), or from the input population to all neurons (Shuffled_3_). Results depicted in (**b**), (**d**) and (**e**) were gathered from 10 simulations per condition.

The baseline, homogeneous circuit already exhibits these properties (Fig 7a), with an average intrinsic time constant (measured as the decay of the membrane potential autocorrelation functions, see Materials and methods) of ≈ 126.75 ms in the quiet state and a relatively broad dynamic range. Thus, even when all the system’s components are homogeneous, neurons appear to operate at relatively long time scales. This can be partially attributed to the fact that each input elicits very small, sub-millivolt responses [137, 138] and these neurons are strongly hyperpolarized. Therefore, the microcircuit complexity, in combination with the nature of the sub-threshold dynamics, is inherently sufficient to allow it to operate over a large and long temporal range and to rapidly switch to the timescales of its primary driving input (Fig 7a, top).

In order to reliably compute, it is beneficial if the system’s high-dimensional dynamics become transiently ‘enslaved’ by the input [16]. This would correspond to a switch in the system’s intrinsic timescale to the dominant input time scale, allowing the intrinsic fluctuations to help the circuit track the input dynamics. The observed features of having a broad dynamic range in the quiet state, along with the ability to rapidly switch to the shorter timescale of the driving input during active processing appear to stem primarily from the microcircuit’s composition and dynamics (homogeneous condition). However, they are present to greater extents in the presence of structural and neuronal heterogeneity (see Fig 7a). The same pattern is true for memory capacity, whereby neuronal heterogeneity causes a large extension of the circuit’s memory range, leading to a slowly fading and relatively long memory store (Fig 7c and 7d).

We express the memory functions as the ability to use the current state of the circuit *x*[*n*] to reconstruct the input sequence at different time lags *u*[*n* − *k*] for *k* ∈ [0, 1, 2, …, *T*] (as depicted in Fig 7c). The total memory capacity C_M_ (Fig 7d) is then the sum of the individual capacities at different lags *k* and thus characterizes the maximum extent to which information about past inputs is retained in the current state. It is worth noting, however, that these results should be interpreted cautiously as the different circuits exhibit different responsiveness to the input signal. For comparability, the time constant at which the input varies (Δ*t*) was chosen independently for each condition as the value that maximizes the circuit’s ability to reconstruct its input, i.e. the value that allowed optimal performance at 0 time lag (see Fig 7b and S4 Fig).

The memory range of structurally heterogeneous networks is similar to that of the fully heterogeneous circuit (C_M_ ≈ 3.65 and 3.49, respectively) and both are markedly larger than in the corresponding homogeneous case (C_M_ ≈ 2.67, Fig 7c and 7d). Thus, despite having a barely noticeable effect on microcircuit dynamics and state transitions, structural heterogeneity appears to have non-negligible functional effects. This is somewhat surprising, in light of all the results discussed so far, but consistent with e.g. [139], who proposed that a heterogeneous network structure can give rise to broad and diverse temporal tuning.

The fact that physiological diversity in single neuron properties (neuronal heterogeneity) extends the dynamic range and memory capacity, is to be expected since it directly decreases redundancy and adds variability to the population responses. However, the magnitude of the effect is very significant, nearly doubling the memory capacity, thus making neuronal heterogeneity stand-out as the most functionally relevant condition. In the presence of neuronal heterogeneity alone, the circuit becomes much more responsive, with broader temporal tuning and memory capacity (Fig 7) and capable of achieving optimal performance in reconstructing the input signal, even when it varies at short timescales (peak reconstruction performance is achieved with Δ*t* ≈ 3.9 ms versus Δ*t* ≈ 13.3 ms in the homogeneous circuit, see Fig 7b and S4 Fig).

Counter-intuitively, it appears that synaptic heterogeneity makes the circuit ‘sluggish’, in the sense that it appears to be less responsive and incapable of tracking fast fluctuations in the input signal (Δ*t* ≈ 20 ms, Fig 7b and S4 Fig). These circuits are also endowed with a very short memory capacity (C_M_ ≈ 2.7) that is similar to that of the homogeneous circuit. Accordingly, diversity in synaptic components enforces a very narrow temporal tuning, skewed towards short timescales in the quiet state (mean *τ*_int_ ≈ 30 ms) and reduces the circuit’s ability to acquire the input timescale (Fig 7a and 7b).

These, apparently deleterious, effects of synaptic heterogeneity hint at an important limitation of our implementation (further discussed in *Limitations and future work*): heterogeneous connection strengths in biological circuits are not randomly assigned, but result from learning and adaptation processes and sub-serve the development of a functional architecture see, e.g. [185], tailored to the circuit’s processing demands. For simplicity, however, we did not introduce any form of synaptic adaptation in our microcircuit model and we have randomly distributed the connection strengths across the network, which may have precluded us from capturing the functional relevance of synaptic heterogeneity.

In order to investigate whether non-random features of the distribution of synaptic weights play a role in the memory capacity, we perform an additional test whereby the weight distributions were retained as in the original implementation, but the individual values were re-shuffled according to three different assumptions about which connections are most likely to have the strongest weights (see Fig 7e): connections within the sub-population of E neurons that were directly stimulated (Shuffled_1_); connections between these input-driven neurons and other E neurons (Shuffled_2_); and connections from the input-driven neurons to every other neuron in the microcircuit (Shuffled_3_, i.e. also involving synapses onto I_1_ and I_2_ populations). The results depicted in Fig 7e demonstrate that any of these modifications improves the circuit’s memory capacity, relative to the random synaptic heterogeneity condition. These improvements are only marginal (reaching a maximum value of C_M_ ≈ 2.78), but substantiate the claim that the non-random nature of synaptic heterogeneity is functionally meaningful and ought to be carefully scrutinized. Among the conditions tested, the strengthening of excitatory synapses connecting the input-driven neurons to the remaining E neurons (Shuffled_2_) appears to be the most beneficial.

Overall, these results demonstrate a clear relation between the system’s responsiveness to temporal fluctuations in the input signal, the intrinsic timescales that characterize the neurons’ activity and the circuit’s memory capacity. There appears to be a ‘push-and-pull’ phenomenon caused by the interactions of neuronal and synaptic heterogeneity, whereby the first significantly boosts the circuit’s dynamic range and memory capacity, whereas the second pulls it back to values similar to the homogeneous condition. Additionally, the independent sources of heterogeneity co-modulate each other’s effects in unexpected ways (Fig 7d). For example, structural and neuronal heterogeneity, which individually cause the most noticeable positive impact on the circuit’s memory capacity, fail to do so when combined. On the other hand, the negative impact of synaptic heterogeneity alone is ameliorated when it is combined with either neuronal or structural heterogeneity.

#### Processing capacity

**Fig 8:**
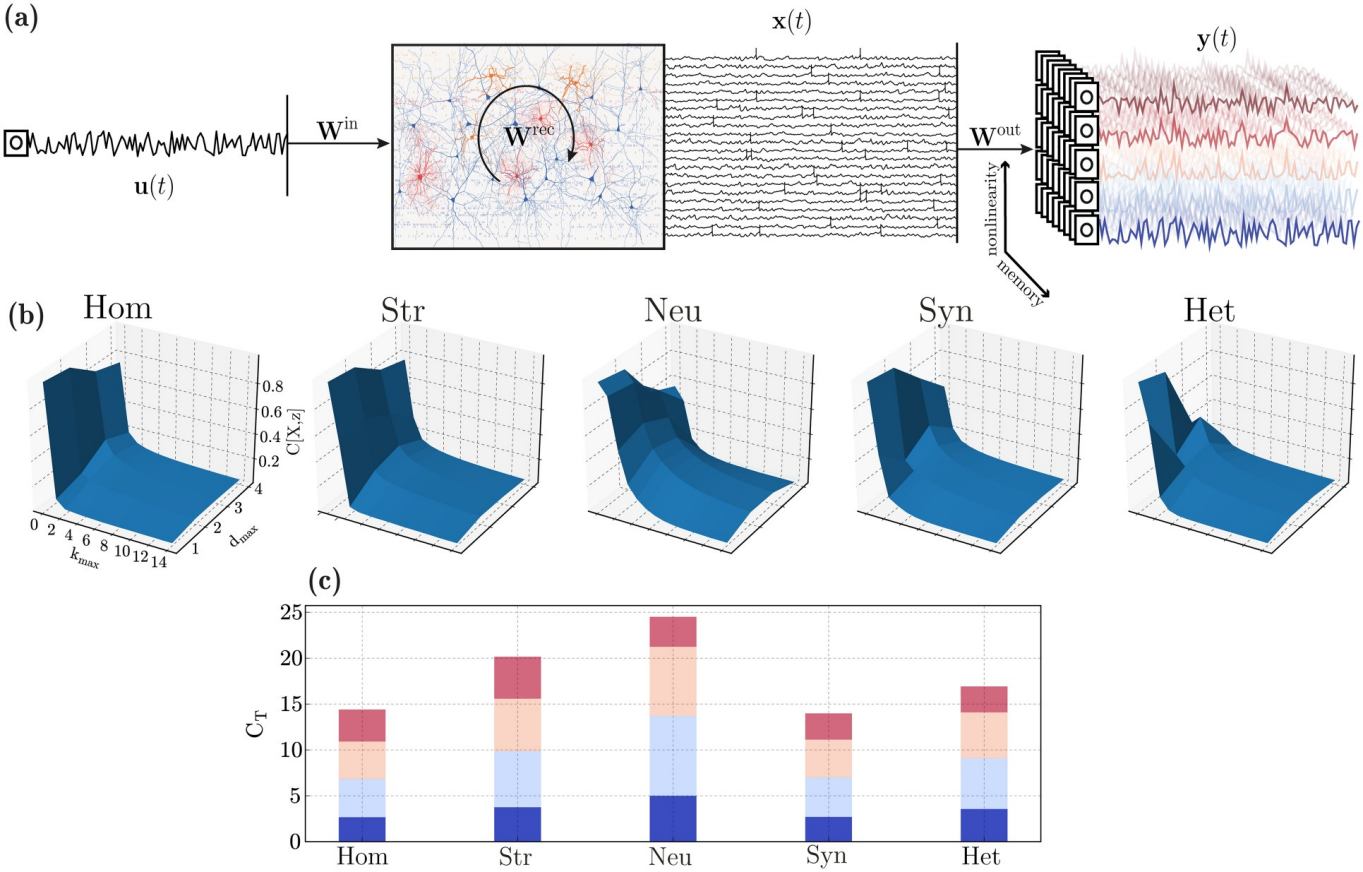
Computational capacity in the different heterogeneity conditions. (**a**) Illustration of the setup used to assess computational capacity. An input signal, *u*, is used to drive a sub-set of E neurons and the population responses, *x*, are recorded and gathered in state matrix *X*. These states are then used to reconstruct a set of time-dependent functions *z* = *f*(*u*^−*k*^). These target functions vary in complexity (degree of nonlinearity, color-coded) and memory requirements. (**b**) Normalized capacity space, i.e. ability to reconstruct functions of *u* at different maximum delays (k_max_, memory) and degrees (d_max_, complexity/nonlinearity). For any given function, the capacity is normalized such that C[X, z] = 1 corresponds to perfect reconstruction of *z*. (**c**) Total processing capacity, expressed as the sum of all capacities for a given degree (the incremental color code in each bar corresponds to the maximum degree for each segment, varying from 1 to 4 and is also illustrated in (**a**)).

To complement the results of the previous sections and determine the microcircuit’s suitability for online processing with fading memory, we adopt the notion of information processing capacity introduced in [140], which allows us to quantify the system’s ability to employ different modes of information processing and, by combining them, determine the total computational capacity of the circuit (for a formal description, see *Processing capacity* in Materials and methods). By this definition, the memory capacity discussed in the previous section corresponds to the capacity to reconstruct the set of *k* different linear functions (degree 1 Legendre polynomials) of the input *u*, each corresponding to a specific time lag, see also [141]. As such, it corresponds to the fraction of the total capacity associated with linear functions (since no products are involved) and measures the circuit’s linear processing capacity (*d* = 1 in Fig 8). Accordingly, degrees *d* ≥ 2 correspond to larger and increasingly complex sets of non-linear basis functions (products of Legendre polynomials, see illustrative example in Fig 8a) and thus require increasingly more sophisticated computational capabilities.

We can thus distinguish between computational complexity / non-linearity (specified by the maximum degree of the basis functions used, *d*_max_) and memory (specified by the maximum delay taken into consideration, *k*_max_). By evaluating a very large set of functions of *u*, we can quantify the circuit’s information processing capacity over the space of basis functions (Fig 8b). The total capacity C_T_ (Fig 8b), defined as the sum of the individual capacities (C[X, z]) for all the different functions *z* tested, thus quantifies the circuit’s ability to compute multiple transformations of *u*, with variable degrees of complexity and provides a summarized description of the system’s information processing capacity (see *Processing capacity* in Materials and methods).

In line with the results on the previous section, heterogeneity in neuronal parameters has the most significant effect, greatly extending the space of computable functions, both linear and nonlinear. By allowing the circuit to retain contextual information for longer (extended memory range), these circuits have a high capacity even for relatively large delays, as demonstrated by the slowly decaying memory curves in Fig 8b. As a consequence, the total capacity of microcircuits with heterogeneous neurons is the largest among all the conditions (Fig 8c).

Despite its very modest effects on population activity, structural heterogeneity has very interesting consequences on the microcircuit’s processing capacity, particularly in the ability to compute complex nonlinear functions. Although the memory functions decay abruptly (almost as abruptly as in the homogeneous condition, Fig 8b), the circuits achieve a larger capacity for more complex functions (Fig 8c) and the total capacity at *d* = 4 (largest degree evaluated) is the largest among all conditions tested, as can be seen by comparing the top crimson bars in Fig 8c.

Also in line with the results for *d* = 1 (linear memory capacity), synaptic heterogeneity has a deleterious effect on processing capacity, reducing it to values smaller than the homogeneous case (C_T_ ≈ 13.9 versus C_T_ ≈ 14.4 for the homogeneous condition, Fig 8c). Consequently, the beneficial effects introduced by both structural and neuronal heterogeneity are counteracted by the negative effect of synaptic heterogeneity, as observed in the previous section. These cancelling effects result in the fully heterogeneous circuit having a total capacity that is only modestly superior to the homogeneous case (C_T_ ≈ 16.9).

Under idealized conditions, the total capacity is bound by the number of linearly independent state variables of the dynamical system, which, in the limit of *T* → ∞, equals N (the number of neurons), in systems that perfectly obey the fading memory property and whose neurons’ activity is linearly independent (for proofs, see [140]). In that respect, the values we have obtained for the total capacity are very modest and close to only 1% of the theoretical limit. On the one hand, this is due to methodological limitations (we could only investigate a short range of d_max_), and on the other, suggests that there may be important aspects that were neglected in this study that significantly boost the total capacity and, at least partially explain the difference (see *Limitations and future work*).

Nevertheless, the results demonstrate a consistent pattern to that observed in the memory capacity, i.e. the functional consequences of the different sources of heterogeneity are consistent for both the ability to compute linear and non-linear functions with fading memory, as circuits with the largest linear memory capacity are also the ones with the largest non-linear processing capacity (namely neuronal heterogeneity). However, these results indicate that neuronal heterogeneity has its main effect on memory (k_max_), greatly extending the capacity to compute functions of *u*_−*k*_ for larger values of *k* (Fig 8b) while structural heterogeneity (that has the second largest functionally beneficial effects) boosts the ability to compute more complex functions, with a main effect on d_max_.

## Discussion

Heterogeneity and diversity in cellular, biochemical and physiological properties seen within and across cortical regions and layers exerts a significant influence on population dynamics. Although often disregarded by the reduced models commonly used in computational neuroscience, these features of the neural tissue may well be partially responsible for the high computational proficiency and functional properties of these systems. In order to understand the functional relevance of the different ‘building blocks’ and their inherent complexity and diversity, it is important to start from relatively simple formalisms and gradually account for the biological complexity while maintaining coherence with the relevant empirical observations at multiple levels. The present study proposes a data-driven modelling approach as an exploratory strategy to systematically uncover the computational benefits of different microcircuit features in an attempt to elucidate and quantify the biophysical substrates of neural computation.

We have focused on the composition of layer 2/3 cortical microcircuits, since their highly recurrent connectivity [142, 143] and sparse, asynchronous activity [93, 111, 113, 117, 142, 144] are ideally suited to study the nature of sparse distributed processing in cortical microcircuit models. The relatively small extent of the neuritic processes (in comparison with deeper layers), makes it acceptable to assume that the potential role of dendritic compartmentalization [145, 146] and other effects caused by the detailed neuronal morphology [147, 148] are negligible, allowing us to use simple point-neuron models with limited loss—which would not be the case if we accounted for the deeper layers. Additionally, the input/output relations and unique position of layer 2/3 in a cortical column suggests a particularly prominent computational role as it must integrate and process multiple streams of information in meaningful ways.

In an attempt to disentangle the role played by heterogeneity in different components of the system, we tentatively partitioned it into neuronal, structural and synaptic components (see Introduction, *Data-driven microcircuit model* and Supplementary Materials). These different sources of heterogeneity differentially influence the characteristics of population responses: from introducing variability in how different neurons and neuronal classes respond to and integrate their synaptic inputs, to variations in the magnitude and distribution of those inputs, among other effects. These, often subtle, differences have complex effects at the population level and strongly condition the system’s operating point. The fully heterogeneous circuit provides the closest approximation to the biophysical reality, exhibiting important commonalities, and appears to inherit different features from different sources of heterogeneity. Naturally, given the simplifications required, our conclusions on the effects of the various heterogeneities are primarily qualitative. However, the extent to which the model responses differ from the empirical observations can also be informative about the potential impact of microcircuit features and processes that were not explicitly considered or were overlooked or oversimplified, as we discuss in the following section.

Collecting, validating and organizing experimental data relevant for these type of studies is still a monumental challenge. Manual annotation and parameter extraction are cumbersome, error-prone strategies and only feasible on well-constrained systems and well-defined problems. The creation and active curation of stable and reliable large-scale databases (of which good examples exist: Allen Brain Atlas [149], NeuroMorpho [150], NeuroElectro [100], NMC [91], ICGenealogy [151], to name a few), along with standard and widely accepted registration and sharing practices [152, 153] are increasingly a priority in a community-driven effort to better constrain neuroscience models and integrate knowledge from multiple disciplines. In addition, automated parameter extraction, estimation as well as model fitting and comparison is strictly and increasingly necessary for studies in this direction. These are complex challenges as they must meet the requirements of an ever-changing scientific field that, consequently, doesn’t lend itself easily to standardization.

Apart from the considerable efforts to explicitly include and account for experimental data to constrain the microcircuit models and make use of publicly available datasets, we additionally emphasize the importance of ensuring transparency, openness and reproducibility. To this end, the complete materials for this study are publicly available through the [154] (see S2 Appendix for details). Our efforts in that direction are a mere example and proof-of-concept, but demonstrate that the field is mature enough to embrace these practices, which should become a standard in computational neuroscience (see also [155]). Given the complexity of these studies, the ability to reproduce and verify are paramount not only to impose the scientific ‘golden standards’, but to extend and build upon existing work.

### Limitations and future work

Despite providing a significant step towards biological verisimilitude, our results demonstrate important limitations that ought to be addressed in future work. At the neuron level, and even though we consider three different neuronal populations, including two separate inhibitory classes, further sub-divisions have been reported in neocortical layer 2/3 populations, both for glutametergic [46] and, in particular, for GABAergic neurons [113, 144, 156–158]. It is possible that these reflect regional specializations particularly prominent in specific cortical areas (such as the prefrontal cortical regions; [34, 46, 159]) or that they represent separate instances of broader classes and can, for simplicity, be grouped together. Parameterized correctly, our choice of neuron model proved to be sufficient for the purposes of this study and allowed us to account for the most important physiological characteristics of the different neuronal classes and their relations (see *Neuronal properties*). Such simplifying assumptions, however, are bound to miss relevant structural and functional features, particularly when it comes to specialization of inhibitory neurons and synapses [160–164], the effects of dendritic nonlinearities and active dendritic processes [145–148], intrinsic adaptation processes [165], to name a few. It is also important, in future work along this direction, to consider the intricate relations between model parameters, i.e. explicitly include not only the empirical variability but also the covariance across multiple parameters (as e.g. [101]). In this context, it should be pointed that the neuronal heterogeneity condition entailed a modification of a larger number of parameters than the other forms of heterogeneity, and so further work is needed to disentangle their contributions and obtain a single-parameter level comparison of their effects. Overall, our results lead us to conclude that it is important to understand the role of multiple interacting populations (e.g. [166]), particularly including inhibitory sub-types and their different physiological properties and interactions, given their clearly distinct contributions.

When it comes to synaptic transmission, we have focused on the specificities of instantaneous response kinetics and its inherent diversity, disregarding any form of synaptic plasticity. However, in our model, synaptic heterogeneity was shown to severely constrain the microcircuit’s processing capacity and memory (Fig 8), counteracting the benefits introduced by neuronal and structural heterogeneity. Additionally, the fact that the total measured capacity is very modest even in the best-performing systems (only about 1% of the theoretical maximum), and considering the computational requirements posed on these systems in ecological conditions, this suggests it is reasonable to assume that there are important aspects of synaptic transmission that we have failed to consider, but contribute significantly to the circuit’s processing capacity. Adaptation and plasticity are likely to be important missing components, due to their critical roles in learning and memory processes [55]. Furthermore, variability in synaptic parameters, being the result of adaptive processes, is bound to reflect the circuit’s functional architecture, as demonstrated in e.g. [167]. Failure to consider the specificities of cortical connectivity is partially responsible for the absence of a substantial functional impact of synaptic heterogeneity in this study.

Throughout this study, we have investigated the behaviour of our microcircuit model in two dynamic regimes, which we associated with the biological *quiet* and *active* states. However, the stimulation applied to bring the circuits into the active state was not biologically realistic, as we purposefully removed any spatiotemporal structure in order to measure the computational properties of the system and not the acquisition of structural information present in the input signal. Thus, the degree to which we are able to account for and explicitly compare empirical observations with the model is restricted and only qualitative. Moreover, whilst measuring the capacity of the network, we significantly under-sampled the space, as the results clearly demonstrate (Fig 8a). A more complete set of basis functions would lead the capacity along both axes to decay to 0: as the complexity and memory requirements increase, the capacity to compute these functions decreases to negligible values in all systems. While accounting for delays of up to k_max_ = 100 allowed us to capture this effect (since the memory range in all conditions is inferior to that), we failed to account for a sufficiently large d_max_. The primary reason for this was computational cost, as our current implementation is extremely time-consuming (see S2 Appendix). As a consequence, the capacity space is sub-normalized, incomplete and underestimated, due to the relatively small number of basis functions tested. Additionally, the limited sample size (*T* = 10^5^) may bias the individual results.

While we have explored information processing capacity in a generic sense, future work along these lines would benefit from being more directed towards specific microcircuits engaged in specific computations. For example, the specific role of layer 2/3 microcircuits in primary and secondary visual cortices for long-range perceptual grouping have been systematically explored [14, 15, 168] and constitute a fruitful avenue for future research.

### Cortical states

The state of any given cortical microcircuit, both in terms of macroscopic spiking statistics and, particularly, membrane potential dynamics can differ dramatically between behavioural states [94, 114, 131, 132] given that they require different levels of active ‘engagement’. The three neuronal classes behave in very specific ways, with specialized response features providing differential contributions to the different circuit states. These neuron-class-specific contributions play an important role in the observed dynamics, providing a potential mechanism to support state modulations [123, 169].

Spontaneous cortical activity during states of *quiet wakefulness* (a quiescent state in which the animal is awake but the circuit is not directly engaged in active processing), is commonly characterized by short-lasting, large amplitude depolarizations [132, 170, 171] that reflect the presence of strongly synchronized excitatory inputs and resemble the dynamics observed under light anaesthesia [114, 132, 138, 144]. Naturally, driven by a homogeneous Poisson process, the system does not exhibit such behaviour (see Results section on *Emergent population dynamics*), which indicates that such effects are partially inherited by the spatiotemporal structure of the background input [172], which in turn may reflect the structure of the sensory input [173]. Additionally, or alternatively, this may be a consequence of propagating waves of excitation [171] which are likely related to spatial connectivity features that were not taken into consideration in this study (see *Limitations and future work*).

Nevertheless, our *quiet* state, where the circuit is driven by background noise, highlights relevant features of population activity and their relations among different neuronal classes, emerging from the effects of the different sources of heterogeneity. The most prominent feature is the extremely sparse firing of E neurons (Fig 4c), which appears to stem directly from the circuit’s composition (homogeneous condition) and is a robust and replicable effect emerging as a direct consequence of dense, strong and fast inhibition. While structural heterogeneity has no measurable effects, synaptic heterogeneity makes the E population more responsive and places some of these neurons closer to their firing thresholds (Fig 4d). Neuronal heterogeneity, on the other hand, leads to more strongly hyperpolarized E and I_1_ populations, compared to all other conditions. This has the positive effect of shifting the distribution of membrane potential in the I_1_ population to a range that overlaps with the empirical values in [94]. However, E neurons become excessively hyperpolarized and their membrane potentials are kept farther from threshold and farther from the corresponding experimental value (to which all other conditions provide a better match). Despite these differences, neuronal heterogeneity is responsible for placing all three neuronal populations operating within the range of values reported in the literature (dashed lines in Fig 4d).

Neocortical pyramidal neurons (particularly in layer 2/3) fire very sparsely and are never driven to saturation, despite a large and constant synaptic bombardment. For this to occur, excitatory and inhibitory input currents onto each neuron must be carefully balanced such that, on average, they cancel each other, allowing the net mean input to be small and the output rates moderate [95, 174]. Co-active and balanced excitation and inhibition thus stabilizes and shapes the circuit’s activity and must be actively maintained to allow the networks to operate in stable regimes [127, 175, 176]. Importantly, it also plays a critical role in active processing and computation, with the most clear experimental evidence coming form the development of input selectivity in visual and auditory cortices (see e.g. [138, 177, 178] and references therein). We demonstrate that such balance condition is an emergent property from circuits with heterogeneous neurons, without the need for changing any of the system’s parameters. This observation may also provide a complementary mechanism by which cortical circuits are able to achieve and retain this dynamic balance, despite the large, potentially disruptive, variations introduced by other sources of heterogeneity, without necessarily requiring specific compensatory mechanisms as has been recently proposed by [86].

### Heterogeneity and information processing

At any given point in time, the state of the circuit reflects a nonlinear combination of the current and past inputs, mediated by complex recurrent interactions. The state of each neuron is thus a nonlinear, fading memory function of the input (the characteristics of which are determined by the circuit’s specificities and input encoding) and the population state a set of *N* basis functions that can be linearly combined to approximate arbitrary nonlinear functions with fading memory. In that sense, these circuits are endowed with universal computing power on time invariant functions [16–18, 134]. This is where complexity and heterogeneity play a particularly prominent role, as they can greatly extend the space of computable functions by diversifying population responses and, consequently, the *richness* of the circuit’s high-dimensional state-space.

With specific functions in mind, circuits can be “designed” to perform certain computations by explicitly solving the credit-assignment problem, i.e. determining how each neuron ought to contribute to the computation [179] in order to achieve the desired outcome. This is typically achieved by constraining the microcircuit connectivity [180, 181] and/or by postulating and building-in specific functionality (e.g. efficient coding; [128, 182]). The great majority of these approaches, however, assumes idealized conditions and neglects the complexities of real biophysics (but see, e.g. [183]), which limits their scope and generalizability.

Since we were not interested in specific functions, but in universal computational properties, instead of “designing” functional microcircuits or assuming specific computations, we sought to mimic fundamental design principles of the real neocortical microcircuitry and systematically evaluate how they affect the circuit’s computational capabilities. While this exploratory approach has its limitations, we were able to partially disentangle the computational role of complexity and heterogeneity in the microcircuit’s building blocks and pinpoint potential sources of functional specialization. Globally, the functional analysis on the computational benefits of the different sources of heterogeneity revealed the same effect: neuronal diversity, on its own, significantly boosts linear and nonlinear processing capacity and memory (see Results sections on memory and processing capacity) and dramatically increases its dynamic range and sensitivity. Surprisingly, and even though its effects on population activity were barely noticeable, structural heterogeneity has the second largest computational effect, particularly boosting the ability to compute highly nonlinear functions (capacity at *d* = 4 was much larger than any other condition, see Fig 8).

The functional benefits introduced by neuronal and structural heterogeneity are not reflected in the fully heterogeneous circuit, given that synaptic heterogeneity prevents this from happening. It would be expectable and desirable that the computational benefits would combine in a way that could dramatically increase the total capacity of the most realistic condition. As discussed above, these synaptic effects likely reflect important limitations in our ability to capture their real influence in the biological system. Nevertheless, some of these results are in line with recent works on the effects of heterogeneity and complexity. In particular, the impact of structural heterogeneity in both macro- and microscopic connectivity have been the subject of recent investigations and are increasingly recognized as critical sources of functional specialization, endowing a network with broad and diverse temporal tuning [139] and providing important contributions to efficient memory storage and robust recall in attractor networks [184, 185].

Despite limitations in our study, discussed above, our results highlight the importance of developing new theories of cortical function and dynamics based on the complex interactions of multiple neuronal sub-populations, as different neuronal classes have a non-negligible differential contribution to the circuit’s dynamics. Additionally, the prominent functional role of structural and neuronal heterogeneity suggest that they are part of a critical minimum necessary to account for computation in cortical microcircuit models as their effects appear to underlie a variety of important phenomena.

## Materials and methods

### Neuronal dynamics

In all systems analysed, the neuronal dynamics is modelled using a common, simplified adaptive leaky integrate-and-fire scheme [98], where the total current flow across the membrane of neuron *i* is governed by:

$$\;C_{\text{m}}\frac{dV_{i}}{dt}=-\;g_{\text{leak}}(V_{i}\left(t\right)-\;E_{L})-I_{i,\text{adapt}}\left(t\right)-\sum_{k\in \;\text{syn}}\sum_{j\in \text{pre}}I_{\;\text{ij}}^{k}\left(t\right)$$ (1)

The spike times of neuron *i* are defined as the set *F*(*i*) = {*t*_*f*_|*V*_*i*_(*t*_*f*_)≥*V*_thresh_}. At these times, the membrane potential is reset to the constant value *V*(*t*) = *V*_reset_ for all times *t* ∈ (*t*_*f*_, *t*_*f*_ + *t*_refr_], after which integration is resumed as above. *I*(*t*) is the total synaptic current generated by inputs from all pre-synaptic neurons *j* ∈ *pre* mediated by synapse type *k* ∈ *syn*.

To provide greater control over neuronal excitability properties and a more realistic account of cortical neuronal dynamics, we model intrinsic adaptation processes as proposed by [99]:

$$\tau_{\text{w}}\frac{dI_{i,\text{adapt}}}{dt}=-I_{i,\text{adapt}}+a(V_{i}\left(t\right)-\;E_{L})+b\sum_{t_{f}\in F\left(i\right)}\delta (t-t_{f})$$ (2)

where the parameters *a* and *b* determine the relative contribution of sub-threshold and spike-triggered adaptation processes, respectively.

### Synaptic dynamics

The synaptic current ($I_{\text{ij}}^{\;\text{syn}}$) elicited by each spike from presynaptic neuron *j* is determined by the conductivity (*G*^rec^) of the corresponding, responsive receptors (each synapse type being composed of a pre-determined set of receptors, see below):

$$I_{\text{ij}}^{\;\text{syn}}\left(t,V_{i}\right)=w_{\text{ij}}^{\;\text{syn}}\sum_{k\in \;\text{rec}}G_{\text{ij}}^{k}\left(t,V_{i}\right)(V_{i}\left(t\right)-E_{k})$$ (3)

The amplitude of post-synaptic currents is rescaled by the dimensionless weight parameter ($w_{\text{ij}}^{\;\text{syn}}$), specific to each connection type and whose value was chosen, such that the PSP amplitudes matched the data reported in [92, 93] (see Table 3). The synaptic conductivity $G_{\text{ij}}^{k}$ in Eq 3 models the response of receptor *k* to spike arrival from pre-synaptic neuron *j* with a total conduction delay of $d_{\text{ij}}^{\;\text{syn}}$:

$$G_{\text{ij}}^{\;\text{rec}}\left(t,V_{i}\right)=\sum_{t_{f}\in F_{j}}g_{\text{ij}}^{\;\text{rec}}\left(t-t_{f}-d_{\text{ij}}^{\;\text{syn}},V_{i}\right)$$ (4)

The conductance transient elicited by a single pre-synaptic event on a single post-synaptic receptor is then modelled as [99, 107–109]:

$$g_{\text{ij}}^{\;\text{rec}}\left(t,V_{i}\right)={\bar{g}}_{\;\text{rec}}n_{\;\text{rec}}\left(V_{i}\right)([1-\text{exp}(-\frac{t}{\tau_{\text{rise}}^{\;\text{rec}}})][r_{\;\text{rec}}\text{exp}(-\frac{t}{\tau_{{\text{decay}}_{f}}^{\;\text{rec}}})+(1-r_{\;\text{rec}})\text{exp}(-\frac{t}{\tau_{{\text{decay}}_{s}}^{\;\text{rec}}})]\Theta \left(t\right))$$ (5)

where ${\bar{g}}_{\;\text{rec}}$ is the peak conductance of the corresponding receptor, *n*_rec_(*V*) is a voltage-gating function assuming a constant value of 1 for all receptor types, except NMDA, in which case [186]:

$$n_{\;\text{NMDA}}\left(V_{i}\right)=(1+\frac{\left[{\text{Mg}}^{2+}\right]}{3.57\text{mM}}\text{exp}\left(-0.062V_{i}\right){)}^{-1}$$ (6)

This gating function is thus used to model the voltage-dependent magnesium block at NMDA receptors. For simplicity, we assume a fixed [Mg^2+^] = 1 mM. The remaining parameters in Eq 5 correspond to the receptors’ characteristic time constants, namely the rise, fast and slow decay times, as well as the relative balance between fast and slow decay (*r*_rec_). In order to account for the differential receptor composition and expression across different neuronal classes, all these parameters are specific for each receptor, synapse and neuron type.

### Generating structural heterogeneity

Consider the sparse adjacency matrix *A*^syn^, specifying the anatomical connectivity between all neurons in source population *pre* and target population *post* (with *pre*, *post* ∈ {E, I_1_, I_2_}). The indices i, j of the nonzero entries in *A*^syn^ are independently drawn from normalized, truncated exponential distributions, with probability:

$$P_{\;\text{pre}}\left(j\right)=\frac{k^{\text{out}}}{\;N^{\;\text{pre}}}\text{exp}(-\frac{jk^{\text{out}}}{\;N^{\;\text{pre}}})$$ (7)

$$P_{\;\text{post}}\left(i\right)=\frac{k^{\text{in}}}{\;N^{\;\text{post}}}\text{exp}(-\frac{ik^{\text{in}}}{\;N^{\;\text{post}}})$$ (8)

for pre- and postsynaptic neuron indices, respectively. N^pre/post^ is the total number of pre-/postsynaptic neurons and *k*^out/in^ are the parameters used to define the skewness of the out-/in-degree distributions, respectively. Setting k^out/in^ = 0 corresponds to a random, uniform connectivity, whereas values >0 generate structured in-/out-degree distributions, with a larger variance in the number of connections per neuron.

#### Weight correlations

For each existing connection, the individual synaptic efficacies ($w_{\;\text{ij}}^{\;\text{syn}}$) can be equal to a fixed scalar value (homogeneous synaptic condition) or randomly drawn from a lognormal distribution (heterogeneous condition). To introduce weight correlations, we specify additional scaling variables *ζ* for each pre- or postsynaptic neuron (*ζ*_j_, *ζ*_i_), whose values are independently drawn from $\text{log}N(-{\left(c_{\text{in}/\text{out}}^{\;\text{syn}}\right)}^{2}/2,c_{\text{in}/\text{out}}^{\;\text{syn}})$. The parameters $c_{\text{in}/\text{out}}^{\;\text{syn}}$ determine the strength of induced correlations, which we fix and set to the values reported in [110] and [71]. The original weight values $w_{\;\text{ij}}^{\;\text{syn}}$ are then re-scaled by *ζ* of the corresponding pre- and postsynaptic partner, i.e.:

$$w_{\text{ij}}=w_{\text{ij}}\zeta_{i}\zeta_{j}$$ (9)

For completeness, and given that lognormal distributions are widely employed throughout this study, it is worth noting that the lognormal probability density function has the following form:

$$P\left(x\right)=\frac{1}{x\sigma \sqrt{2\pi }}\text{exp}(-\frac{\text{ln}{\left(x-\mu \right)}^{2}}{2\sigma^{2}})$$ (10)

parameterized by scale (*μ*) shape (*σ*) values.

### Profiling the microcircuits

To adequately quantify the relevant functional properties of the microcircuits and the impact of the different features analysed, we employ metrics that are system-agnostic, i.e. independent from the specificities of the circuit analysed and, preferably, parameter-free such that the choices of metric parameters do not influence the measured outcome and any results obtained are unbiased and objectively reflect the circuit’s properties. Of particular interest, for the purposes of this study, is the adequate quantification of the characteristics of population dynamics, under active synaptic bombardment as well as the circuit’s capacity for stimulus processing and computation, in order to establish links between the features of population dynamics, the circuit’s composition and complexity and its ability to perform complex computations.

#### Input specifications

We model cortical background / ongoing activity as an unspecific and stochastic external input driving the circuit, considered to be excitatory, i.e. mediated by glutamatergic synapses, and to consist of independent Poissonian spike trains, at a fixed rate *ν*_*in*_ spikes/s. Given the small network size and, in order to compensate for the relatively small numbers of synapses involved, we rescale the input rates by a constant factor, K_in_ = 1000, such that each neuron receives, on average, background input through K_in_ synapses, with each presynaptic source firing at a fixed rate of *ν*_*in*_ spikes/s. The postsynaptic neuron’s responsiveness to this background input is then determined by its specific receptor composition (the kinetics of AMPA and NMDA receptors), with synaptic weights and delays equal to any other excitatory synapse onto that neuron, i.e. $w_{\text{in}}=\mu_{w}^{\alpha \;E}$ and $d_{\text{in}}=\mu_{d}^{\alpha \;E}$, where *α* refers to the postsynaptic neuron class (see Table 3).

In addition, to emulate an active state and evaluate the microcircuit’s processing capacity, we introduce an additional input signal, directly encoded as a somatic input current (*I*_*in*_(*t*)), which changes every Δ*t* ms, and deliver it to a randomly chosen sub-set 25% of the E population. We choose this direct encoding strategy in order to ensure that the input signal has a direct influence on the membrane dynamics, making it easier to decode [187–189]. As further specified below, the values of this piece-wise constant input current were independent and identically drawn (i.i.d.) from a uniform distribution over the interval [0, 1] and scaled by a constant factor *ρ*_u_, independently chosen for each condition (see S2 Fig).

#### Population dynamics

To quantify and characterize the population responses to different input conditions and assess how the different sources of heterogeneity modulate those responses, we look at the statistics of spiking activity as well as the relevant sub-threshold dynamics across the different neuronal populations.

The circuit’s state or operating point is typically determined primarily by the firing rate and the degree of population-wide synchrony and regularity [78], as measured by the following statistics:

*Synchrony*.—average spike count correlation coefficient (CC), computed pair-wise, for a large number of *n*^pairs^ = 500 randomly sampled, disjoint, neuronal pairs (see, e.g. [115]).

*Regularity*.—degree of dispersion of the inter-spike interval (ISI) distribution, as measured by the coefficient of variation (CV_ISI_), averaged across the population. A value of 0 indicates a clock-like, regular firing pattern, whereas a completely irregular, Poisson process has a value of 1. Values larger than 1 are obtained for very bursty firing patterns.

*Burstiness*.—degree of burstiness in the firing patterns can be captured by the 5-th percentile of the ISI distribution (ISI_5%_), averaged across the population. This measure has been successfully used to classify neuronal firing patterns in identified populations [190, 191]. A low value indicates higher burstiness, for a given rate.

*Randomness*.—The entropy of the log-ISI distribution is an important metric that captures the randomness in a spike train [192]. It was recently demonstrated that this metric was one of the key features of cerebellar neurons’ spike trains, allowing their accurate identification [190]. This metric is defined on the probability density of the natural logarithm of the ISIs:

$$H_{\text{ISI}}=-\sum_{i=1}^{N}p\left(I_{i}\right){\text{log}}_{2}\left(p\left(I_{i}\right)\right)$$ (11)

This last metric is added for completeness, and can be seen as an additional measure of regularity. The higher the entropy value, the more irregular the firing pattern.

On the sub-threshold level, we assess and summarize the characteristics of membrane potential dynamics, synaptic currents and conductances. We analyse the distributions of mean membrane potentials (〈V_m_〉) and their variances (*σ*^2^(V_m_)), as well as the mean excitatory and inhibitory synaptic currents (〈I^syn^〉) onto each neuron. For the computational analyses, we consider the dynamics of the membrane potentials the main state variable [189].

*Intrinsic timescale* (*τ*_int_).—to quantify the characteristic timescale of population activity, we look at the autocorrelation function of each neuron’s membrane potentials:

$$R_{i}\left(\tau_{lag}\right)=\frac{\text{Cov}(V_{i}\left(t\right),V_{i}\left(t-\tau_{lag}\right))}{\sqrt{\text{Var}\left(V_{i}\left(t\right)\right)\text{Var}\left(V_{i}\left(t-\tau_{lag}\right)\right)}}$$ (12)

For each neuron, we fit the autocorrelation by an exponential function:

$$R_{i}\left(\tau_{lag}\right)=a\times [\text{exp}(-\tau_{lag}/\tau_{\text{int}})+b]$$ (13)

where *τ*_int_ specifies the decay time constant, characterizing the neuron’s intrinsic timescale.

#### Processing capacity

To analyse the processing capacity of the networks, following [141], we begin by defining an input sequence *u*[*n*], of finite total length *T*, comprising values independent- and identically drawn (i.i.d.) from a pre-determined probability distribution *p*(*u*). Since we are interested in measuring the circuit’s generic processing properties and not specific transformations on specific inputs, considering the input a random variable ensures that it has no pre-imposed structure so that any measured structure reflects only the system’s intrinsic properties and not the acquisition of structural relations present in the input. We set *p*(*u*) to be the uniform distribution over the interval [0, 1]. This input sequence is then directly encoded into the circuit as explained above.

The circuit’s initial states are randomized ($V_{0}∼U_{\left[E_{L},V_{th}\right]}$) and the circuit is driven by the input, for a total simulation time of *T*×Δ*t*. In order to obtain accurate results and diminish potential errors and biases, we use a large sample size of *T* = 10^5^. The circuit state in response to the input is sampled at every Δ*t* ms, resulting in a collection of state vectors *x*[*n*] corresponding to a sample of the circuit state at time point t* = *n* × Δ*t* ms. The resulting state matrix $X\in R_{\;N_{\;E}\times T}$ and the corresponding input $u\in R_{1\times T}$ will then be used to estimate the capacity.

The aim of the analysis is to quantify the system’s ability to carry out computations on *u*. For that purpose, we measure the capacity *C* to reconstruct time-dependent functions *z* on finite sequences of *k* inputs, *z*[*n*] = *z*(*u*^−*k*^[*n*]), from the state of the system, using a simple linear estimator:

$$C\left[X,z\right]=\frac{(X^{T}X{)}^{-1}Xz}{{\left||z|\right|}^{2}}$$ (14)

The numerator in the capacity measure corresponds to the linear estimator that minimizes the quadratic error between the target function to be reconstructed *z* and its linear estimate $\hat{z}$:

$$W^{\text{out}}={\text{argmin}}_{W^{\text{out}}}(\sum_{n=1}^{T}{\left(z\left[n\right]-\hat{z}\left[n\right]\right)}^{2})=(X^{T}X{)}^{-1}Xz$$ (15)

where

$$\hat{z}=W^{\text{out}}X$$ (16)

For any given function *z* and observed states *X*, C[X, z] is normalized, such that, in a system that allows perfect reconstruction of *z*, C[X, z] = 1, meaning that there exists a linear combination of *x*[*n*] that equals *z*[*n*], for all *n*. On the other hand, a capacity of 0 indicates that it is not possible to even partially reconstruct the target function. Evaluating C[X, z] for large sets of target functions *y*_{*l*}_ = {*z*_1_, …, *z*_*L*_}, allows us to gain insights into the information processing capacity of the system. If the evaluated functions are sufficiently distinct (preferably orthogonal), their corresponding capacities measure independent properties and provide independent information about how the system computes. As such, we systematically probe the capacity space by evaluating the complete set of orthonormal basis functions of *u*, using finite products of normalized Legendre polynomials:

$$y_{\left\{d_{k}\right\}}=\prod_{k}P_{d_{k}}\left(u\left[n-k\right]\right))$$ (17)

where $P_{d_{k}}\left(.\right)$ is the Legendre polynomial of degree *d*_*k*_ ≥ 0:

$$P_{d}\left(s\right)=\frac{1}{2^{d}}\sum_{i=0}^{d}(\begin{array}{c} d\\ i \end{array}{)}^{2}{\left(s-1\right)}^{d-i}{\left(s+1\right)}^{i}$$ (18)

and is a function of input *u* delayed by *k* steps. The total capacity then corresponds to the sum of the individual capacities for a given set of target functions *y*_{*d*_*k*_}_:

$$C_{\left\{d_{k}\right\}}=\sum_{\left\{d_{k}\right\}}C\left[X,\left\{d_{k}\right\}\right]$$ (19)

Naturally, we use finite data and a finite set of indices *d* to evaluate the capacities, leading to an unavoidable underestimation of the total capacity.

#### Linear memory and nonlinearity

Using the notations introduced above, and following [140, 141], we can consider the linear memory capacity as the total capacity associated with linear functions:

$$C_{M}=\sum_{\left\{d_{k}\right\}}\delta (\sum_{k}d_{k}-1)C\left[X,\left\{d_{k}\right\}\right]$$ (20)

for a maximum polynomial degree of *d* = 1, i.e. each of the functions tested corresponds to a delayed version of the input:

$$z\left[n\right]=P_{1}\left(u\left[n-k\right]\right)=\left(u\left[n-k\right]\right),\forall k\in \left[0,k_{max}\right]$$ (21)

Accordingly, the capacity associated with nonlinear functions corresponds to *d* ≥ 2. For more details on the implementation, consult [141] and the code we provide in the Supplementary Materials.

### Numerical simulations, implementation and data analysis

All the work presented in this manuscript was implemented using the Neural Microcircuit Simulation and Analysis Toolkit (NMSAT) [193], a python package designed to provide the first steps towards complex microcircuit benchmarking, as suggested and exemplified in this study. The core simulation engine running all the numerical simulations is NEST. Due to the specificities of this project, we used a modified version of NEST 2.10.0 [194], which includes all the models used in this manuscript (some of which are not available in the main release). A complete code package is provided in the supplementary materials that implements project-specific functionality to the framework, allowing the reproduction of all the numerical experiments presented in this manuscript. Computing resources were provided by the JARA-HPC Vergabegremium on the supercomputer JURECA [195] at Forschungszentrum Jülich. All numerical simulations were performed at a resolution of 0.1 ms, using the GSL implementation of the adaptive fourth-order Runge-Kutta method.

## Supporting information

S1 Table Tabular description of network model after [196]. (PDF)

S2 Table Discrepancies between electrophysiological parameters reported in the literature and model results. The results obtained after careful choice of the individual parameters for the different neuronal classes did not exactly match the experimental reports, but the relative relations between classes are retained. (*) Note that the ranges reported in this table are a rough approximation to the range of mean values reported in different studies (see below). Naturally, values like the maximum rate (*ν*_max_[Hz]) depend entirely on the range of input current considered in a given experiment, so in this case, only the relative ratio is pertinent.
(PDF)

S1 Appendix Primary data sources. List of the main references used to constrain model parameters.
(PDF)

S2 Appendix Reproducibility and replication. (PDF)

S1 Fig Population rate transfer functions. Characteristics of population spiking activity in response to background, Poissonian input (quiet state) in the various conditions analysed and for the 3 different population types (E, blue; I_1_, red; I_2_, orange), as a function of the input rate *ν*_in_ for a total simulation time of 10 seconds. The top row corresponds the population rate transfer functions, showing that E neurons fire extremely sparsely and synaptic heterogeneity is strictly required to obtain an active E population. The middle and bottom row depict the measured irregularity (CV_ISI_) and synchrony (CC) in all conditions analysed. Note that in many conditions the spiking activity in the E population is so sparse that it is not possible to compute these metrics, since the total number of spikes is insufficient.
(TIF)

S2 FigTuning the input parameters for the active state.To emulate an active processing condition, an extra input current of maximum amplitude *ρ*_u_ is given to a randomly chosen subset of 25% excitatory neurons. The circuits in the different conditions exhibit different degrees of sensitivity to their inputs. To achieve adequate and comparable responses, we attempt to find combination of input parameters that allows the mean firing rates to remain within realistic bounds (*ν*_E_ ∈ [0.5, 5], $\nu_{\;I_{1}}\in \left[10,25\right]$, $\nu_{\;I_{2}}\in \left[3,15\right]$).(TIF)

S3 Fig Population spiking activity in the active state. Complete statistics of population spiking activity in the active state for the different neuron classes: E (top, blue), I_1_ (middle, red) and I_2_ (bottom, orange) and for the different conditions (columns). The radial axes in each plot correspond to: regularity (CV_ISI_), synchrony (CC), burstiness (ISI_5%_), entropy of the ISI distribution (H_ISI_), and the mean firing rate (*ν*). All statistics were computed for an observation period of 10s, in a single realization for each condition, with all input parameters fixed and set to the values determined in S2 Fig.
(TIF)

S4 Fig Temporal receptivity of the microcircuits analysed. (**a**) capacity to reconstruct the original input signal at zero lag (i.e. maximum polynomial degree *d* = 1, maximum delay *k* = 0, C_d=1,k=0_) as a function of the signal resolution (Δ*t*). Since the capacity values converge asymptotically to 1, we determine the optimal resolution as the minimum Δ*t* at which C_d=1,k=0_ ≥ 0.99. (**b**) Decoding capacity at minimum resolution Δ*t* = 0.1 ms (equal to the simulation resolution). (**c**) Capacity at the maximum resolution tested (Δ*t* = 20 ms). (**d**) Optimal resolution for each condition. All results correspond to the mean and standard deviations for 10 simulations per condition.
(TIF)

S1 File Software package. (GZ)

## References

[1] Koch C. Complexity and the Nervous System. Science. 1999;284(5411):96–98. 10.1126/science.284.5411.96 10102826

[2] Duarte R, Seeholzer A, Zilles K, Morrison A. Synaptic patterning and the timescales of cortical dynamics. Current Opinion in Neurobiology. 2017;43:156–165. 10.1016/j.conb.2017.02.007 28407562

[3] Gjorgjieva J, Drion G, Marder E. Computational implications of biophysical diversity and multiple timescales in neurons and synapses for circuit performance. Current Opinion in Neurobiology. 2016;37(Table 1):44–52. 10.1016/j.conb.2015.12.008 26774694 PMC4860045

[4] Singer W. Complexity as Substrate for Neuronal Computations. Complexity and Analogy in Science: Theoretical, Methodological and Epistemological Aspects. 2015;22:209–218.

[5] Otopalik AG, Sutton AC, Banghart M, Marder E. When complex neuronal structures may not matter. eLife. 2017;6:e23508 10.7554/eLife.23508 28165322 PMC5323043

[6] Bélanger M, Allaman I, Magistretti PJ. Brain energy metabolism: Focus on Astrocyte-neuron metabolic cooperation; 2011 Available from: http://www.sciencedirect.com/science/article/pii/S1550413111004207. 10.1016/j.cmet.2011.08.016 22152301

[7] Mappes J, Lindstrom L. How Did the Cuckoo Get Its Polymorphic Plumage? Science. 2012;337(6094):532–533. 10.1126/science.1225997 22859476

[8] Edelman GM, Gally JA. Degeneracy and complexity in biological systems. Proceedings of the National Academy of Sciences. 2001;98(24):13763–13768. 10.1073/pnas.231499798 PMC61115 11698650

[9] Price CJ, Friston KJ. Degeneracy and cognitive anatomy. Trends in Cognitive Sciences. 2002;6(10):416–421. 10.1016/S1364-6613(02)01976-9 12413574

[10] Krakauer JW, Ghazanfar AA, Gomez-Marin A, MacIver MA, Poeppel D. Neuroscience Needs Behavior: Correcting a Reductionist Bias. Neuron. 2017;93(3):480–490. 10.1016/j.neuron.2016.12.041 28182904

[11] Marom S. On the Precarious Path of Reverse Neuro-Engineering. Frontiers in Computational Neuroscience. 2009;3(May):3–6. 19503751 10.3389/neuro.10.005.2009 PMC2691154

[12] Hopfield JJ. Physics, Computation, and Why Biology Looks so Different; 1994 Available from: http://www.sciencedirect.com/science/article/pii/S0022519384712112.

[13] Getting P. Emerging Principles Governing The Operation Of Neural Networks. Annual Review of Neuroscience. 1989;12(1):185–204. 10.1146/annurev.ne.12.030189.001153 2648949

[14] Grossberg S, Mingolla E. Neural dynamics of perceptual grouping: Textures, boundaries, and emergent segmentations. Perception & Psychophysics. 1985;38(2):141–171. 10.3758/BF03198851 4088806

[15] Léveillé J, Versace M, Grossberg S. Running as fast as it can: How spiking dynamics form object groupings in the laminar circuits of visual cortex. Journal of Computational Neuroscience. 2010;28(2):323–346. 10.1007/s10827-009-0211-1 20111896

[16] Thalmeier D, Uhlmann M, Kappen HJ, Memmesheimer RM. Learning Universal Computations with Spikes. PLoS Computational Biology. 2016;12(6):1–29. 10.1371/journal.pcbi.1004895 PMC4911146 27309381

[17] Maass W, Natschlager T, Markram H. Fading memory and kernel properties of generic cortical microcircuit models. Journal of Physiology Paris. 2004;98(4-6 SPEC. ISS.):315–330. 10.1016/j.jphysparis.2005.09.020 16310350

[18] Maass W, Natschlager T, Markram H. Real-time computing without stable states: A new framework for neural computation based on perturbations. Neural computation. 2002;14(11):2531–2560. 10.1162/089976602760407955 12433288

[19] Mountcastle VB. The columnar organization of the neocortex. Brain. 1997;120(4):701–722. 10.1093/brain/120.4.701 9153131

[20] Mountcastle V. An organizing principle for cerebral function: the unit model and the distributed system In: Edelman GM, Mountcastle V, editors. The Mindful Brain. MIT Press; 1978 p. 7–50. Available from: http://www.citeulike.org/group/8299/article/4545635.

[21] Park HJ, Friston K. Structural and functional brain networks: from connections to cognition. Science (New York, NY). 2013;342(6158):1238411 10.1126/science.1238411 24179229

[22] Meunier D, Lambiotte R, Bullmore ET. Modular and hierarchically modular organization of brain networks. Frontiers in Neuroscience. 2010;4(DEC):1–11. 21151783 10.3389/fnins.2010.00200 PMC3000003

[23] Friston K. A theory of cortical responses. Philosophical transactions of the Royal Society of London Series B, Biological sciences. 2005;360(1456):815–36. 10.1098/rstb.2005.1622 15937014 PMC1569488

[24] VanEssen DC. Cartography and connectomes. Neuron. 2013;80(3):775–790. 10.1016/j.neuron.2013.10.027 24183027 PMC3855872

[25] Shinomoto S, Shima K, Tanji J. Differences in spiking patterns among cortical neurons. Neural computation. 2003;15(12):2823–2842. 10.1162/089976603322518759 14629869

[26] Pletikos M, Sousa A, Sedmak G, Meyer K, Zhu Y, Cheng F, et al Temporal specification and bilaterality of human neocortical topographic gene expression. Neuron. 2014;81(2):321–332. 10.1016/j.neuron.2013.11.018 24373884 PMC3931000

[27] Kang HJ, Kawasawa YI, Cheng F, Zhu Y, Xu X, Li M, et al Spatio-temporal transcriptome of the human brain. Nature. 2011;478(7370):483–9. 10.1038/nature10523 22031440 PMC3566780

[28] Hawrylycz MJ, Lein ES, Guillozet-Bongaarts AL, Shen EH, Ng L, Miller JA, et al An anatomically comprehensive atlas of the adult human brain transcriptome. Nature. 2012;489(7416):391–9. 10.1038/nature11405 22996553 PMC4243026

[29] Zilles K, Palomero-Gallagher N, Schleicher A. Transmitter receptors and functional anatomy of the cerebral cortex. Journal of Anatomy. 2004;205(6):417–432. 10.1111/j.0021-8782.2004.00357.x 15610391 PMC1571403

[30] O’Rourke NA, Weiler NC, Micheva KD, Smith SJ. Deep molecular diversity of mammalian synapses: why it matters and how to measure it. Nature reviews Neuroscience. 2012;13(6):365–79. 10.1038/nrn3170 22573027 PMC3670986

[31] Mueller S, Wang D, Fox MD, Yeo BTT, Sepulcre J, Sabuncu MR, et al Individual Variability in Functional Connectivity Architecture of the Human Brain. Neuron. 2013;77(3):586–595. 10.1016/j.neuron.2012.12.028 23395382 PMC3746075

[32] Yeo BTT, Krienen FM, Sepulcre J, Sabuncu MR, Lashkari D, Hollinshead M, et al The organization of the human cerebral cortex estimated by intrinsic functional connectivity. Journal of neurophysiology. 2011;106:1125–1165. 10.1152/jn.00338.2011 21653723 PMC3174820

[33] Power JD, Cohen AL, Nelson SM, Wig GS, Barnes KA, Church JA, et al Functional Network Organization of the Human Brain. Neuron. 2011;72(4):665–678. 10.1016/j.neuron.2011.09.006 22099467 PMC3222858

[34] Harris KD, Shepherd GMG. The neocortical circuit: themes and variations. Nature Neuroscience. 2015;18(2):170–181. 10.1038/nn.3917 25622573 PMC4889215

[35] Shinomoto S, Kim H, Shimokawa T, Matsuno N, Funahashi S, Shima K, et al Relating neuronal firing patterns to functional differentiation of cerebral cortex. PLoS Computational Biology. 2009;5(7). 10.1371/journal.pcbi.1000433 19593378 PMC2701610

[36] Tripathy SJ, Burton SD, Geramita M, Gerkin RC, Urban NN. Brain-wide analysis of electrophysiological diversity yields novel categorization of mammalian neuron types. Journal of Neurophysiology. 2015;113(10):3474–3489. 10.1152/jn.00237.2015 25810482 PMC4455486

[37] Poulin JF, Tasic B, Hjerling-Leffler J, Trimarchi JM, Awatramani R. Disentangling neural cell diversity using single-cell transcriptomics. Nature Neuroscience. 2016;19(9):1131–1141. 10.1038/nn.4366 27571192

[38] Palomero-Gallagher N, Zilles K. Cortical layers: Cyto-, myelo-, receptor- and synaptic architecture in human cortical areas. NeuroImage. 2017 10.1016/j.neuroimage.2017.08.035 28811255

[39] Zilles K, Palomero-Gallagher N, Grefkes C, Scheperjans F, Boy C, Amunts K, et al Architectonics of the human cerebral cortex and transmitter receptor fingerprints: Reconciling functional neuroanatomy and neurochemistry. European Neuropsychopharmacology. 2002;12(6):587–599. 10.1016/S0924-977X(02)00108-6 12468022

[40] Zilles K, Amunts K. Receptor mapping: architecture of the human cerebral cortex. Current Opinion in Neurology. 2009;22(4):331–339. 10.1097/WCO.0b013e32832d95db 19512925

[41] Wagstyl K, Ronan L, Goodyer IM, Fletcher PC. Cortical thickness gradients in structural hierarchies. NeuroImage. 2015;111:241–250. 10.1016/j.neuroimage.2015.02.036 25725468 PMC4401442

[42] Finlay BL, Uchiyama R. Developmental mechanisms channeling cortical evolution. Trends in Neurosciences. 2015;38(2):69–76. 10.1016/j.tins.2014.11.004 25497421

[43] Collins CE, Airey DC, Young NA, Leitch DB, Kaas JH. Neuron densities vary across and within cortical areas in primates. Proceedings of the National Academy of Sciences of the United States of America. 2010;107(36):15927–32. 10.1073/pnas.1010356107 20798050 PMC2936588

[44] Brown SP, Hestrin S. Intracortical circuits of pyramidal neurons reflect their long-range axonal targets. Nature. 2009;457(7233):1133–1136. 10.1038/nature07658 19151698 PMC2727746

[45] Stepanyants A, Chklovskii DB. Neurogeometry and potential synaptic connectivity; 2005 Available from: http://linkinghub.elsevier.com/retrieve/pii/S0166223605001311. 10.1016/j.tins.2005.05.006 15935485

[46] Zaitsev AV, Povysheva NV, Gonzalez-Burgos G, Lewis DA. Electrophysiological classes of layer 2/3 pyramidal cells in monkey prefrontal cortex. Journal of Neurophysiology. 2012;108(2):595–609. 10.1152/jn.00859.2011 22496534 PMC3404790

[47] Mensi S, Naud R, Pozzorini C, Avermann M, Petersen CCH, Gerstner W. Parameter extraction and classification of three cortical neuron types reveals two distinct adaptation mechanisms. Journal of Neurophysiology. 2012;107(6):1756–1775. 10.1152/jn.00408.2011 22157113

[48] Van Aerde KI, Feldmeyer D. Morphological and physiological characterization of pyramidal neuron subtypes in rat medial prefrontal cortex. Cerebral Cortex. 2015;25(3):788–805. 10.1093/cercor/bht278 24108807

[49] Lisman JE, Raghavachari S, Tsien RW. The sequence of events that underlie quantal transmission at central glutamatergic synapses. Nature Reviews Neuroscience. 2007;8(8):597–609. 10.1038/nrn2191 17637801

[50] Südhof TC, Malenka RC. Understanding Synapses: Past, Present, and Future. Neuron. 2008;60(3):469–476. 10.1016/j.neuron.2008.10.011 18995821 PMC3243741

[51] Marx V. A deep look at synaptic dynamics. Nature. 2014;515(7526):293–297. 10.1038/515293a 25391965

[52] Sabatini BL, Regehr WG. Timing of Synaptic Transmission. Annual Review of Physiology. 1999;61(1):521–542. 10.1146/annurev.physiol.61.1.521 10099700

[53] Greengard P. The Neurobiology of Slow Synaptic Transmission. Science. 2001;294(5544):1024–1030. 10.1126/science.294.5544.1024 11691979

[54] Südhof TC. Neurotransmitter release: The last millisecond in the life of a synaptic vesicle. Neuron. 2013;80(3):675–690. 10.1016/j.neuron.2013.10.022 24183019 PMC3866025

[55] Abbott LF, Regehr WG. Synaptic computation. Nature. 2004;431(7010):796–803. 10.1038/nature03010 15483601

[56] Voglis G, Tavernarakis N. The role of synaptic ion channels in synaptic plasticity. EMBO reports. 2006;7(11):1104–1110. 10.1038/sj.embor.7400830 17077866 PMC1679792

[57] Hestrin S. Different glutamate receptor channels mediate fast excitatory synaptic currents in inhibitory and excitatory cortical neurons. Neuron. 1993;11(6):1083–1091. 10.1016/0896-6273(93)90221-C 7506044

[58] Moreau AW, Kullmann DM. NMDA receptor-dependent function and plasticity in inhibitory circuits. Neuropharmacology. 2013;74:23–31. 10.1016/j.neuropharm.2013.03.004 23537500

[59] Angulo MC, Rossier J, Audinat E. Postsynaptic glutamate receptors and integrative properties of fast-spiking interneurons in the rat neocortex. Journal of neurophysiology. 1999;82(3):1295–1302. 10.1152/jn.1999.82.3.1295 10482748

[60] Nissen W, Szabo A, Somogyi J, Somogyi P, Lamsa KP. Cell Type-Specific Long-Term Plasticity at Glutamatergic Synapses onto Hippocampal Interneurons Expressing either Parvalbumin or CB1 Cannabinoid Receptor. Journal of Neuroscience. 2010;30(4):1337–1347. 10.1523/JNEUROSCI.3481-09.2010 20107060 PMC2817897

[61] Destexhe A, Mainen ZF, Sejnowski TJ. Kinetic models of synaptic transmission In: Koch C, Segev I, editors. Methods in Neuronal Modeling. 2nd ed Cambridge, MA: MIT Press; 1998 p. 1–25. Available from: http://cns.iaf.cnrs-gif.fr/abstracts/KSchap96.html.

[62] Destexhe A, Mainen ZF, Sejnowski TJ. Synthesis of models for excitable membranes, synaptic transmission and neuromodulation using a common kinetic formalism. Journal of Computational Neuroscience. 1994;1(3):195–230. 10.1007/BF00961734 8792231

[63] Kubota Y, Karube F, Nomura M, Kawaguchi Y. The Diversity of Cortical Inhibitory Synapses. Frontiers in Neural Circuits. 2016;10:27 10.3389/fncir.2016.00027 27199670 PMC4842771

[64] Markram H, Lübke J, Frotscher M, Roth A, Sakmann B. Physiology and anatomy of synaptic connections between thick tufted pyramidal neurones in the developing rat neocortex. Journal of Physiology. 1997;500(2):409–440. 10.1113/jphysiol.1997.sp022031 9147328 PMC1159394

[65] Song S, Sjostrom PJ, Reigl M, Nelson S, Chklovskii DB. Highly nonrandom features of synaptic connectivity in local cortical circuits. PLoS Biology. 2005;3(3):0507–0519. 10.1371/journal.pbio.0030068 PMC1054880 15737062

[66] Thomson AM. Synaptic Connections and Small Circuits Involving Excitatory and Inhibitory Neurons in Layers 2-5 of Adult Rat and Cat Neocortex: Triple Intracellular Recordings and Biocytin Labelling In Vitro. Cerebral Cortex. 2002;12(9):936–953. 10.1093/cercor/12.9.936 12183393

[67] Perin R, Berger TK, Markram H. A synaptic organizing principle for cortical neuronal groups. Proceedings of the National Academy of Sciences. 2011;108(13):5419–5424. 10.1073/pnas.1016051108 PMC3069183 21383177

[68] Yoshimura Y, Dantzker JLM, Callaway EM. Excitatory cortical neurons form fine-scale functional networks. Nature. 2005;433(7028):868–873. 10.1038/nature03252 15729343

[69] Yoshimura Y, Callaway EM. Fine-scale specificity of cortical networks depends on inhibitory cell type and connectivity. Nature Neuroscience. 2005;8(11):1552–1559. 10.1038/nn1565 16222228

[70] Shimono M, Beggs JM. Functional clusters, hubs, and communities in the cortical microconnectome. Cerebral Cortex. 2015;25(10):3743–3757. 10.1093/cercor/bhu252 25336598 PMC4585513

[71] Tomm C, Avermann M, Petersen C, Gerstner W, Vogels TP. Connection-type-specific biases make uniform random network models consistent with cortical recordings. Journal of Neurophysiology. 2014;112(8):1801–1814. 10.1152/jn.00629.2013 24944218 PMC4200009

[72] Koulakov AA, Hromadka T, Zador AM. Correlated Connectivity and the Distribution of Firing Rates in the Neocortex. Journal of Neuroscience. 2009;29(12):3685–3694. 10.1523/JNEUROSCI.4500-08.2009 19321765 PMC2784918

[73] Roxin A. The Role of Degree Distribution in Shaping the Dynamics in Networks of Sparsely Connected Spiking Neurons. Frontiers in Computational Neuroscience. 2011;5:8 10.3389/fncom.2011.00008 21556129 PMC3058136

[74] Pernice V, Deger M, Cardanobile S, Rotter S. The relevance of network micro-structure for neural dynamics. Frontiers in computational neuroscience. 2013;7(June):72 10.3389/fncom.2013.00072 23761758 PMC3671286

[75] Litwin-Kumar A, Doiron B. Slow dynamics and high variability in balanced cortical networks with clustered connections. Nature Neuroscience. 2012;15(11):1498–1505. 10.1038/nn.3220 23001062 PMC4106684

[76] Harris KD, Mrsic-Flogel TD. Cortical connectivity and sensory coding. Nature. 2013;503(7474):51–58. 10.1038/nature12654 24201278

[77] Hoffmann FZ, Triesch J. Nonrandom network connectivity comes in pairs. Network Neuroscience. 2017;1(1):31–41. 10.1162/NETN_a_00004 29601066 PMC5869014

[78] Tsotsos JK, Culhane SM, Wai WYK, Lai Y, Davis N, Nuflo F. Dynamics of Sparsely Conntected Networks of Excitatory and Inhibitory Spiking Neurons. Journal of Computational Neuroscience. 2000;8:183–208. 10.1023/A:1008925309027 10809012

[79] van Vreeswijk C, Sompolinsky H. Chaos in neuronal networks with balanced excitatory and inhibitory activity. Science (New York, NY). 1996;274(5293):1724–6. 10.1126/science.274.5293.1724 8939866

[80] van Vreeswijk C, Sompolinsky H. Chaotic Balanced State in a Model of Cortical Circuits. Neural Computation. 1998;10(6):1321–1371. 10.1162/089976698300017214 9698348

[81] Amit DJ, Brunel N. Model of global spontaneous activity and local structured activity during delay periods in the cerebral cortex. Cerebral Cortex. 1997;7(3):237–252. 10.1093/cercor/7.3.237 9143444

[82] Potjans TC, Diesmann M. The cell-type specific cortical microcircuit: Relating structure and activity in a full-scale spiking network model. Cerebral Cortex. 2014;24(3):785–806. 10.1093/cercor/bhs358 23203991 PMC3920768

[83] Schmidt M, Bakker R, Shen K, Bezgin G, Diesmann M, van Albada SJ. Full-density multi-scale account of structure and dynamics of macaque visual cortex. 2015. 10.1371/journal.pcbi.1006359

[84] Cain N, Iyer R, Koch C, Mihalas S. The Computational Properties of a Simplified Cortical Column Model. PLOS Computational Biology. 2016;12(9):e1005045 10.1371/journal.pcbi.1005045 27617444 PMC5019422

[85] Haeusler S, Maass W. A statistical analysis of information-processing properties of lamina-specific cortical microcircuit models. Cerebral Cortex. 2007;17(1):149–162. 10.1093/cercor/bhj132 16481565

[86] Landau ID, Egger R, Dercksen VJ, Oberlaender M, Sompolinsky H. The Impact of Structural Heterogeneity on Excitation-Inhibition Balance in Cortical Networks. Neuron. 2016;92(5):1106–1121. 10.1016/j.neuron.2016.10.027 27866797 PMC5158120

[87] Chelaru MI, Dragoi V. Efficient coding in heterogeneous neuronal populations. Proceedings of the National Academy of Sciences of the United States of America. 2008;105(42):16344–16349. 10.1073/pnas.0807744105 18854413 PMC2571028

[88] Markram H, Muller E, Ramaswamy S, Reimann MW, Abdellah M, Sanchez CA, et al Reconstruction and Simulation of Neocortical Microcircuitry. Cell. 2015;163(2):456–92. 10.1016/j.cell.2015.09.029 26451489

[89] Markram H. The blue brain project. Nature reviews Neuroscience. 2006;7(2):153–160. 10.1038/nrn1848 16429124

[90] Helmstaedter M, de Kock CPJ, Feldmeyer D, Bruno RM, Sakmann B. Reconstruction of an average cortical column in silico. Brain Research Reviews. 2007;55(2 SPEC. ISS.):193–203. 10.1016/j.brainresrev.2007.07.011 17822776

[91] Ramaswamy S, Courcol JD, Abdellah M, Adaszewski SR, Antille N, Arsever S, et al The neocortical microcircuit collaboration portal: a resource for rat somatosensory cortex. Frontiers in Neural Circuits. 2015;9:44 10.3389/fncir.2015.00044 26500503 PMC4597797

[92] Lefort S, Tomm C, Floyd Sarria JC, Petersen CCH. The Excitatory Neuronal Network of the C2 Barrel Column in Mouse Primary Somatosensory Cortex. Neuron. 2009;61(2):301–316. 10.1016/j.neuron.2008.12.020 19186171

[93] Avermann M, Tomm C, Mateo C, Gerstner W, Petersen CCH. Microcircuits of excitatory and inhibitory neurons in layer 2/3 of mouse barrel cortex. Journal of Neurophysiology. 2012;107(11):3116–3134. 10.1152/jn.00917.2011 22402650

[94] Gentet LJ, Avermann M, Matyas F, Staiger JF, Petersen CCH. Membrane Potential Dynamics of GABAergic Neurons in the Barrel Cortex of Behaving Mice. Neuron. 2010;65(3):422–435. 10.1016/j.neuron.2010.01.006 20159454

[95] Shadlen MN, Newsome WT. The variable discharge of cortical neurons: implications for connectivity, computation, and information coding. The Journal of neuroscience: the official journal of the Society for Neuroscience. 1998;18(10):3870–96. 10.1523/JNEUROSCI.18-10-03870.1998 9570816 PMC6793166

[96] Ecker AS, Berens P, Keliris GA, Bethge M, Logothetis NK, Tolias AS. Decorrelated Neuronal Firing in Cortical Microcircuits. Science. 2010;327(5965):584–587. 10.1126/science.1179867 20110506

[97] Renart A, de la Rocha J, Bartho P, Hollender L, Parga N, Reyes A, et al The Asynchronous State in Cortical Circuits. Science. 2010;327(5965):587–590. 10.1126/science.1179850 20110507 PMC2861483

[98] Koch C. Biophysics of Computation Information Processing in Single Neuron vol. 11 Oxford University Press, USA; 2004 Available from: http://www.amazon.de/Biophysics-Computation-Information-Computational-Neuroscience/dp/0195181999.

[99] Gerstner W, Kistler WM, Naud R, Paninski L. Neuronal Dynamics—from single neurons to networks and models of cognition. Cambridge University Press; 2014 Available from: https://books.google.de/books?id=D4j2AwAAQBAJ.

[100] Tripathy SJ, Savitskaya J, Burton SD, Urban NN, Gerkin RC. NeuroElectro: a window to the world’s neuron electrophysiology data. Frontiers in Neuroinformatics. 2014;8(April):40 10.3389/fninf.2014.00040 24808858 PMC4010726

[101] Harrison PM, Badel L, Wall MJ, Richardson MJE. Experimentally Verified Parameter Sets for Modelling Heterogeneous Neocortical Pyramidal-Cell Populations. PLoS Computational Biology. 2015;11(8):e1004165 10.1371/journal.pcbi.1004165 26291316 PMC4546387

[102] Lu Jt, Li Cy, Zhao JP, Poo Mm, Zhang Xh. Spike-Timing-Dependent Plasticity of Neocortical Excitatory Synapses on Inhibitory Interneurons Depends on Target Cell Type. Journal of Neuroscience. 2007;27(36):9711–9720. 10.1523/JNEUROSCI.2513-07.2007 17804631 PMC6672961

[103] Szabadics J. Excitatory Effect of GABAergic Axo-Axonic Cells in Cortical Microcircuits. Science. 2006;311(5758):233–235. 10.1126/science.1121325 16410524

[104] Hill E, Kalloniatis M, Tan SS. Glutamate, GABA and precursor amino acids in adult mouse neocortex: cellular diversity revealed by quantitative immunocytochemistry. Cerebral cortex (New York, NY: 1991). 2000;10(11):1132–42. 10.1093/cercor/10.11.1132 11053233

[105] Palomero-Gallagher N, Amunts K, Zilles K. Transmitter Receptor Distribution in the Human Brain In: Toga AW, editor. Brain Mapping. San Diego: Elsevier Academic Press; 2015 p. 261–275.

[106] Zilles K, Schleicher A, Palomero-Gallagher N, Amunts K. Quantitative Analysis of Cyto- and Receptor Architecture of the Human Brain. vol. 58; 2002 Available from: http://www.epjap.org/10.1051/epjap/2012110475%5Cnhttp://linkinghub.elsevier.com/retrieve/pii/B978012693019150023X.

[107] McCormick DA, Wang Z, Huguenard J. Neurotransmitter control of neocortical neuronal activity and excitability. Cerebral Cortex. 1993;3(5):387–398. 10.1093/cercor/3.5.387 7903176

[108] Gerstner W, Kistler WM. Spiking Neuron Models. Cambridge University Press; 2002 Available from: http://ebooks.cambridge.org/ref/id/CBO9780511815706.

[109] Hoffmann JHO, Meyer HS, Schmitt AC, Straehle J, Weitbrecht T, Sakmann B, et al Synaptic conductance estimates of the connection between local inhibitor interneurons and pyramidal neurons in layer 2/3 of a cortical column. Cerebral Cortex. 2015;25(11):4415–4429. 10.1093/cercor/bhv039 25761638 PMC4816789

[110] Tomm C. Analysing Neuronal Network Architectures: From Weight Distributions to Structure and Back ÉCOLE POLYTECHNIQUE FÉDÉRALE DE LAUSANNE; 2012 Available from: https://infoscience.epfl.ch/record/174669/files/EPFL_TH5302.pdf.

[111] O’Connor DH, Peron SP, Huber D, Svoboda K. Neural activity in barrel cortex underlying vibrissa-based object localization in mice. Neuron. 2010;67(6):1048–1061. 10.1016/j.neuron.2010.08.026 20869600

[112] Benedetti BL, Takashima Y, Wen JA, Urban-Ciecko J, Barth AL. Differential wiring of layer 2/3 neurons drives sparse and reliable firing during neocortical development. Cerebral Cortex. 2013;23(11):2690–2699. 10.1093/cercor/bhs257 22918982 PMC3792743

[113] Petersen CCH, Crochet S. Synaptic Computation and Sensory Processing in Neocortical Layer 2/3. Neuron. 2013;78(1):28–48. 10.1016/j.neuron.2013.03.020 23583106

[114] Crochet S, Poulet JFA, Kremer Y, Petersen CCH. Synaptic mechanisms underlying sparse coding of active touch. Neuron. 2011;69(6):1160–1175. 10.1016/j.neuron.2011.02.022 21435560

[115] Kumar A, Schrader S, Aertsen A, Rotter S. The High-Conductance State of Cortical Networks. Neural Computation. 2008;20(1):1–43. 10.1162/neco.2008.20.1.1 18044999

[116] Destexhe A, Rudolph M, Paré D. The high-conductance state of neocortical neurons in vivo. Nature Reviews Neuroscience. 2003;4(12):1019–1019. 10.1038/nrn1289 12951566

[117] Waters J, Helmchen F. Background Synaptic Activity Is Sparse in Neocortex. Journal of Neuroscience. 2006;26(32):8267–8277. 10.1523/JNEUROSCI.2152-06.2006 16899721 PMC6673816

[118] Léger J, Stern E, Aertsen A, Heck D. Synaptic integration in rat frontal cortex shaped by network activity. Journal of Neurophysiology. 2005;93(93):281–293. 10.1152/jn.00067.2003 15306631

[119] Destexhe A, Paré D, Gk Q, Pare D. Impact of network activity on the integrative properties of neocortical pyramidal neurons in vivo. Journal of neurophysiology. 1999;81(4):1531–47. 10.1152/jn.1999.81.4.1531 10200189

[120] Humphries MD. The Goldilocks zone in neural circuits. eLife. 2016;5 10.7554/eLife.22735 27911259 PMC5135390

[121] Tsodyks M, Sejnowski T. Rapid state switching in balanced cortical network models. Network: Computation in Neural Systems. 1995;6(2):111–124. 10.1088/0954-898X_6_2_001

[122] Zucca S, D’Urso G, Pasquale V, Vecchia D, Pica G, Bovetti S, et al An inhibitory gate for state transition in cortex. eLife. 2017;6:e26177 10.7554/eLife.26177 28509666 PMC5444901

[123] Poulet JFA. Keeping an Eye on Cortical States. Neuron. 2014;84(2):246–248. 10.1016/j.neuron.2014.10.005 25374350

[124] Kremkow J, Aertsen A, Kumar A. Gating of Signal Propagation in Spiking Neural Networks by Balanced and Correlated Excitation and Inhibition. Journal of Neuroscience. 2010;30(47):15760–15768. 10.1523/JNEUROSCI.3874-10.2010 21106815 PMC6633769

[125] Vogels TP. Signal Propagation and Logic Gating in Networks of Integrate-and-Fire Neurons. Journal of Neuroscience. 2005;25(46):10786–10795. 10.1523/JNEUROSCI.3508-05.2005 16291952 PMC6725859

[126] Vogels TP, Abbott LF. Gating multiple signals through detailed balance of excitation and inhibition in spiking networks. Nature Neuroscience. 2009;12(4):483–491. 10.1038/nn.2276 19305402 PMC2693069

[127] Duarte R, Morrison A. Dynamic stability of sequential stimulus representations in adapting neuronal networks. Frontiers in Computational Neuroscience. 2014;8(October):124 10.3389/fncom.2014.00124 25374534 PMC4205815

[128] Denève S, Machens CK. Efficient codes and balanced networks. Nature Neuroscience. 2016;19(3):375–382. 10.1038/nn.4243 26906504

[129] Rubin R, Abbott LF, Sompolinsky H. Balanced Excitation and Inhibition are Required for High-Capacity, Noise-Robust Neuronal Selectivity. 2017. 10.1073/pnas.1705841114 PMC5676886 29042519

[130] Vogels TP, Sprekeler H, Zenke F, Clopath C, Gerstner W. Inhibitory Plasticity Balances Excitation and Inhibition in Sensory Pathways and Memory Networks. Science. 2011;334(6062):1569–1573. 10.1126/science.1211095 22075724

[131] Crochet S, Petersen CCH. Correlating whisker behavior with membrane potential in barrel cortex of awake mice. Nature Neuroscience. 2006;9(5):608–610. 10.1038/nn1690 16617340

[132] Poulet JFA, Petersen CCH. Internal brain state regulates membrane potential synchrony in barrel cortex of behaving mice. Nature. 2008;454(7206):881–885. 10.1038/nature07150 18633351

[133] Buzsáki G, Mizuseki K. The log-dynamic brain: how skewed distributions affect network operations. Nature Reviews Neuroscience. 2014;15(4):264–278. 10.1038/nrn3687 24569488 PMC4051294

[134] Enel P, Procyk E, Quilodran R, Dominey PF. Reservoir Computing Properties of Neural Dynamics in Prefrontal Cortex. PLoS Computational Biology. 2016;12(6):e1004967 10.1371/journal.pcbi.1004967 27286251 PMC4902312

[135] Nikolić D, Häusler S, Singer W, Maass W, Nikolic D, Hausler S, et al Distributed fading memory for stimulus properties in the primary visual cortex. PLoS Biology. 2009;7(12):e1000260 10.1371/journal.pbio.1000260 20027205 PMC2785877

[136] Maass W. Searching for Principles of Brain Computation. Current Opinion in Behavioral Sciences. 2016;11:81–92. 10.1016/j.cobeha.2016.06.003

[137] Bruno RM. Cortex Is Driven by Weak but Synchronously Active Thalamocortical Synapses. Science. 2006;312(5780):1622–1627. 10.1126/science.1124593 16778049

[138] Okun M, Lampl I. Balance of excitation and inhibition. Scholarpedia. 2009;4(8):7467 10.4249/scholarpedia.7467

[139] Chaudhuri R, Bernacchia A, Wang XJ. A diversity of localized timescales in network activity. eLife. 2014;3:e01239 10.7554/eLife.01239 24448407 PMC3895880

[140] Dambre J, Verstraeten D, Schrauwen B, Massar S. Information Processing Capacity of Dynamical Systems. Scientific Reports. 2012;2(1):514 10.1038/srep00514 22816038 PMC3400147

[141] Jaeger H. Short term memory in echo state networks. GMD Report 152. 2002; p. 60.

[142] Lewis DA, Gonzalez-Burgos G. Intrinsic excitatory connections in the prefrontal cortex and the pathophysiology of schizophrenia. Brain Research Bulletin. 2000;52(5):309–317. 10.1016/S0361-9230(99)00243-9 10922508

[143] Feldmeyer D, Lübke J, Sakmann B. Efficacy and connectivity of intracolumnar pairs of layer 2/3 pyramidal cells in the barrel cortex of juvenile rats. The Journal of Physiology. 2006;575(2):583–602. 10.1113/jphysiol.2006.105106 16793907 PMC1819447

[144] Neske GT, Patrick SL, Connors BW. Contributions of Diverse Excitatory and Inhibitory Neurons to Recurrent Network Activity in Cerebral Cortex. Journal of Neuroscience. 2015;35(3):1089–1105. 10.1523/JNEUROSCI.2279-14.2015 25609625 PMC4300319

[145] Branco T, Häusser M. The single dendritic branch as a fundamental functional unit in the nervous system. Current Opinion in Neurobiology. 2010;20(4):494–502. 10.1016/j.conb.2010.07.009 20800473

[146] Morita K. Possible Role of Dendritic Compartmentalization in the Spatial Working Memory Circuit. Journal of Neuroscience. 2008;28(30):7699–7724. 10.1523/JNEUROSCI.0059-08.2008 18650346 PMC6670839

[147] Spruston N. Pyramidal neurons: dendritic structure and synaptic integration. Nature Reviews Neuroscience. 2008;9(3):206–221. 10.1038/nrn2286 18270515

[148] Kubota Y, Kondo S, Nomura M, Hatada S, Yamaguchi N, Mohamed AA, et al Functional effects of distinct innervation styles of pyramidal cells by fast spiking cortical interneurons. eLife. 2015;4(July 2015):1–27. 10.7554/eLife.07919 PMC4518632 26142457

[149] Sunkin SM, Ng L, Lau C, Dolbeare T, Gilbert TL, Thompson CL, et al Allen Brain Atlas: an integrated spatio-temporal portal for exploring the central nervous system. Nucleic acids research. 2013;41(Database issue):D996–D1008. 10.1093/nar/gks1042 23193282 PMC3531093

[150] Ascoli GA, Donohue DE, Halavi M. NeuroMorpho.Org: a central resource for neuronal morphologies. The Journal of neuroscience: the official journal of the Society for Neuroscience. 2007;27(35):9247–51. 10.1523/JNEUROSCI.2055-07.2007 17728438 PMC6673130

[151] Podlaski WF, Seeholzer A, Groschner LN, Miesenboeck G, Ranjan R, Vogels TP. ICGenealogy: Mapping the function of neuronal ion channels in model and experiment. bioRxiv. 2016; p. 058685. 10.1101/058685 PMC5340531 28267430

[152] Zehl L, Jaillet F, Stoewer A, Grewe J, Sobolev A, Wachtler T, et al Handling Metadata in a Neurophysiology Laboratory. Frontiers in Neuroinformatics. 2016;10:26 10.3389/fninf.2016.00026 27486397 PMC4949266

[153] Peng RD. Reproducible Research in Computational Science. Science. 2011;334(6060):1226–1227. 10.1126/science.1213847 22144613 PMC3383002

[154] Open Science Collaboration. Estimating the reproducibility of psychological science. Science. 2015;349(6251):aac4716–aac4716. 10.1126/science.aac4716 26315443

[155] Pauli R, Weidel P, Kunkel S, Morrison A. Reproducing Polychronization: A Guide to Maximizing the Reproducibility of Spiking Network Models. 2018;12(August):1–21. 10.3389/fninf.2018.00046 PMC6085985 30123121

[156] Ascoli GA, Alonso-Nanclares L, Anderson SA, Barrionuevo G, Benavides-Piccione R, Burkhalter A, et al Petilla terminology: nomenclature of features of GABAergic interneurons of the cerebral cortex. Nature Reviews Neuroscience. 2008;9(7):557–568. 10.1038/nrn2402 18568015 PMC2868386

[157] Helmstaedter M, Sakmann B, Feldmeyer D. L2/3 Interneuron groups defined by multiparameter analysis of axonal projection, dendritic geometry, and electrical excitability. Cerebral Cortex. 2009;19(4):951–962. 10.1093/cercor/bhn130 18802122

[158] Jiang X, Shen S, Sinz F, Reimer J, Cadwell CR, Berens P, et al Response to Comment on “Principles of connectivity among morphologically defined cell types in adult neocortex”. Science. 2016;353(6304):1108–1108. 10.1126/science.aaf6102 27609883

[159] Shepard GM, Grillner S. Handbook of Brain Microcircuits; 2010 Available from: http://oxfordmedicine.com/view/10.1093/med/9780195389883.001.0001/med-9780195389883.

[160] Isaacson JS, Scanziani M. How inhibition shapes cortical activity. Neuron. 2011;72(2):231–243. 10.1016/j.neuron.2011.09.027 22017986 PMC3236361

[161] Fino E, Packer AM, Yuste R. The Logic of Inhibitory Connectivity in the Neocortex. The Neuroscientist. 2013;19(3):228–237. 10.1177/1073858412456743 22922685 PMC4133777

[162] Wilson NR, Runyan CA, Wang FL, Sur M. Division and subtraction by distinct cortical inhibitory networks in vivo. Nature. 2012;488(7411):343–348. 10.1038/nature11347 22878717 PMC3653570

[163] Pi HJ, Hangya B, Kvitsiani D, Sanders JI, Huang ZJ, Kepecs A. Cortical interneurons that specialize in disinhibitory control. Nature. 2013;503(7477):521–524. 10.1038/nature12676 24097352 PMC4017628

[164] Gupta A. Organizing Principles for a Diversity of GABAergic Interneurons and Synapses in the Neocortex. Science. 2000;287(5451):273–278. 10.1126/science.287.5451.273 10634775

[165] Pozzorini C, Naud R, Mensi S, Gerstner W. Temporal whitening by power-law adaptation in neocortical neurons. Nature Neuroscience. 2013;16(7):942–948. 10.1038/nn.3431 23749146

[166] Lagzi F, Rotter S. Dynamics of competition between subnetworks of spiking neuronal networks in the balanced state. PLoS ONE. 2015;10(9). 10.1371/journal.pone.0138947 26407178 PMC4583999

[167] Cossell L, Iacaruso MF, Muir DR, Houlton R, Sader EN, Ko H, et al Functional organization of excitatory synaptic strength in primary visual cortex. Nature. 2015;518(7539):399–403. 10.1038/nature14182 25652823 PMC4843963

[168] von der Heydt R, Peterhans E, Baumgartner G. Illusory contours and cortical neuron responses. Science (New York, NY). 1984;224(4654):1260–2. 10.1126/science.6539501 6539501

[169] Pachitariu M, Stringer C, Okun M, Bartho P, Harris K, Latham P, et al Inhibitory control of shared variability in cortical networks. bioRxiv. 2016;(041103):041103 10.7554/eLife.19695 PMC5142814 27926356

[170] DeWeese MR, Zador AM. Non-Gaussian Membrane Potential Dynamics Imply Sparse, Synchronous Activity in Auditory Cortex. Journal of Neuroscience. 2006;26(47):12206–12218. 10.1523/JNEUROSCI.2813-06.2006 17122045 PMC6675435

[171] Petersen CCH, Hahn TTG, Mehta M, Grinvald A, Sakmann B. Interaction of sensory responses with spontaneous depolarization in layer 2/3 barrel cortex. Proceedings of the National Academy of Sciences. 2003;100(23):13638–13643. 10.1073/pnas.2235811100 PMC263866 14595013

[172] Poulet JFA, Fernandez LMJ, Crochet S, Petersen CCH. Thalamic control of cortical states. Nature Neuroscience. 2012;15(3):370–372. 10.1038/nn.3035 22267163

[173] Luczak A, Bartho P, Marguet SL, Buzsaki G, Harris KD. Sequential structure of neocortical spontaneous activity in vivo. Proceedings of the National Academy of Sciences. 2007;104(1):347–352. 10.1073/pnas.0605643104 PMC1765463 17185420

[174] Shadlen MN, Newsome WT. Noise, neural codes and cortical organization. Current Opinion in Neurobiology. 1994;4(4):569–579. 10.1016/0959-4388(94)90059-0 7812147

[175] Vogels TP, Rajan K, Abbott LF. Neural Network Dynamics. Annual Review of Neuroscience. 2005;28(1):357–376. 10.1146/annurev.neuro.28.061604.135637 16022600

[176] Ernst U, Pawelzik K. Sensible Balance. Science. 2011;334(6062):1507–1508. 10.1126/science.1216483 22174239

[177] Mariño J, Schummers J, Lyon DC, Schwabe L, Beck O, Wiesing P, et al Invariant computations in local cortical networks with balanced excitation and inhibition. Nature Neuroscience. 2005;8(2):194–201. 10.1038/nn1391 15665876

[178] Dorrn AL, Yuan K, Barker AJ, Schreiner CE, Froemke RC. Developmental sensory experience balances cortical excitation and inhibition. Nature. 2010;465(7300):932–936. 10.1038/nature09119 20559387 PMC2888507

[179] Abbott LF, DePasquale B, Memmesheimer RM. Building functional networks of spiking model neurons. Nature Neuroscience. 2016;19(3):350–355. 10.1038/nn.4241 26906501 PMC4928643

[180] Memmesheimer RM, Timme M. Designing complex networks. Physica D: Nonlinear Phenomena. 2006;224(1-2):182–201. 10.1016/j.physd.2006.09.037

[181] Memmesheimer RM, Timme M. Designing the dynamics of spiking neural networks. Physical Review Letters. 2006;97(18):1881011–4. 10.1103/PhysRevLett.97.188101 17155580

[182] Boerlin M, Machens CK, Denève S. Predictive Coding of Dynamical Variables in Balanced Spiking Networks. PLoS Computational Biology. 2013;9(11). 10.1371/journal.pcbi.1003258 24244113 PMC3828152

[183] Schwemmer MA, Fairhall AL, Denéve S, Shea-Brown ET. Constructing precisely computing networks with biophysical spiking neurons. The Journal of Neuroscience. 2014;32(28):10112–10134. 10.1523/JNEUROSCI.4951-14.2015 PMC6605339 26180189

[184] Guzman SJ, Schlögl A, Frotscher M, Jonas P. Synaptic mechanisms of pattern completion in the hippocampal CA3 network. Science. 2016;353(6304):1117–1123. 10.1126/science.aaf1836 27609885

[185] Brunel N. Is cortical connectivity optimized for storing information? Nature Neuroscience. 2016;19(5):749–755. 10.1038/nn.4286 27065365

[186] Jahr CE, Stevens CF. Voltage dependence of NMDA-activated macroscopic conductances predicted by single-channel kinetics. The Journal of neuroscience. 1990;10(9):3178–3182. 10.1523/JNEUROSCI.10-09-03178.1990 1697902 PMC6570236

[187] Eliasmith C, Anderson CH. Neural Engineering: Computation Representation and Dyamics in Neurobiological Systems. vol. 19 MIT Press; 1991.

[188] Weidel P, Djurfeldt M, Duarte R, Morrison A. Closed loop interactions between spiking neural network and robotic simulators based on MUSIC and ROS. Frontiers in Neuroinformatics. 2016;10(31):1–19. 10.3389/fninf.2016.00031 27536234 PMC4971076

[189] van den Broek D, Uhlmann M, Fitz H, Duarte R, Hagoort P, Petersson KM. The best spike filter kernel is a neuron; 2017.

[190] van Dijck G, van Hulle MM, Heiney SA, Blazquez PM, Meng H, Angelaki DE, et al Probabilistic Identification of Cerebellar Cortical Neurones across Species. PLoS ONE. 2013;8(3). 10.1371/journal.pone.0057669 23469215 PMC3587648

[191] Ruigrok TJH, Hensbroek RA, Simpson JI. Spontaneous Activity Signatures of Morphologically Identified Interneurons in the Vestibulocerebellum. Journal of Neuroscience. 2011;31(2):712–724. 10.1523/JNEUROSCI.1959-10.2011 21228180 PMC6623423

[192] Dorval AD. Probability distributions of the logarithm of inter-spike intervals yield accurate entropy estimates from small datasets. Journal of Neuroscience Methods. 2008;173(1):129–139. 10.1016/j.jneumeth.2008.05.013 18620755 PMC2610469

[193] Duarte R, Zajzon B, Morrison A. Neural Microcircuit Simulation And Analysis Toolkit. Zenodo. 2017 10.5281/zenodo.594850

[194] Bos H, Morrison, Abigail Peyser, Alexander Hahne J, Helias M, Kunkel S, Ippen T, Eppler JM, et al. Nest 2.10.0. 2015; p. 10.5281/zenodo.44222

[195] Krause D, Thörnig P. JURECA: General-purpose supercomputer at Jülich Supercomputing Centre. Journal of large-scale research facilities JLSRF. 2016;2(0):A62 10.17815/jlsrf-2-121

[196] Nordlie E, Gewaltig MO, Plesser HE. Towards reproducible descriptions of neuronal network models. PLoS Computational Biology. 2009;5(8):e1000456 10.1371/journal.pcbi.1000456 19662159 PMC2713426

