# Supplementary material for: Leveraging heterogeneity for neural computation with fading memory in layer 2/3 cortical microcircuits
Source: PLoS Comput Biol. 2019 Apr 25;15(4):e1006781. doi: 10.1371/journal.pcbi.1006781 (PMC6504118; doi:10.1371/journal.pcbi.1006781)
Supplement: S1 Table — (PDF) [file pcbi.1006781.s001.pdf]

A: Model Summary	
**Populations**	1 Excitatory, 2 Inhibitory
**Topology**	None
**Connectivity**	Sparse, random or structured, with density p^{syn}
**Neuron Model**	Adaptive leaky integrate-and-fire, fixed voltage threshold, fixed absolute refractory time, sub-threshold and spike-triggered adaptation
**Synapse Model**	Multi-receptor kinetics
**Plasticity**	None
**Input**	Stochastic background spikes and somatic current injection onto 25% E neurons
**Measurements**	Spiking activity, membrane potentials, synaptic currents/conductances

B: Populations		
**Name**	**Elements**	**Size**
E	`iaf_cond_mtime`	2000
I_1	`iaf_cond_mtime`	175
I_2	`iaf_cond_mtime`	325

C: Neuron Models	
**Name**	Multi-adaptive integrate-and-fire neuron (`iaf_cond_mtime`)
**Subthreshold Dynamics**	if $\left(t > t^f + \tau_{\text{ref}}\right)$<br>$\mathrm{C_m}\frac{dV_i}{dt} = -\,\mathrm{g_{leak}}\left(V_i(t) - E_L\right) - I_{i,\text{adapt}}(t) - \sum_{k\in\,\text{syn}}\sum_{j\in\text{pre}} I^k_{ij}(t)$<br>else<br>$V(t) = V_{\text{reset}}$
**Intrinsic Adaptation**	$\tau_{\mathrm{w}}\frac{dI_{i,\text{adapt}}}{dt} = -I_{i,\text{adapt}} + a\left(V_i(t) - \mathrm{E_L}\right) + b\sum_{t_f\in F(i)}\delta\left(t - t_f\right)$
**Synaptic Transmission**	$I^{\text{syn}}_{\text{ij}}(t, V_i) = w^{\text{syn}}_{\text{ij}}\sum_{k\in\,\text{rec}} G^k_{\text{ij}}(t, V_i)\left(V_i(t) - E_k\right)$
**Spiking**	If $V(t-) < V_{th}$ OR $V(t+) \geq V_{th}$<br>1. set $t^f = t$ 2. emit spike with time stamp t^f

D: Synapse Models	
**Receptor Conductance**	$g^{\text{rec}}_{\text{ij}}(t, V_i) = \bar{g}_{\,\text{rec}} n_{\,\text{rec}}(V_i)\left(\left[1 - \exp\left(-\frac{t}{\tau^{\text{rec}}_{\text{rise}}}\right)\right]\right.$ $\left.\left[r_{\,\text{rec}}\exp\left(-\frac{t}{\tau^{\text{rec}}_{\text{decay}_\text{f}}}\right) + (1 - r_{\,\text{rec}})\exp\left(-\frac{t}{\tau^{\text{rec}}_{\text{decay}_\text{s}}}\right)\right]\right)\Theta(t)$

E: Input		
**Type**	**Target**	**Description**
`poisson_generator`	[E, I_1, I_2]	Total rate $\nu_{\text{in}}\mathrm{K_{in}}$
`step_current_generator`	Subset of E	Step current amplitude $u[n]\rho_{\mathrm{u}}$, changing every Δt ms

F: Measurements
Spiking activity, membrane potentials, synaptic currents and conductances
