# Supplementary material for: Leveraging heterogeneity for neural computation with fading memory in layer 2/3 cortical microcircuits
Source: PLoS Comput Biol. 2019 Apr 25;15(4):e1006781. doi: 10.1371/journal.pcbi.1006781 (PMC6504118; doi:10.1371/journal.pcbi.1006781)
Supplement: S2 Table — The results obtained after careful choice of the individual parameters for the different neuronal classes did not exactly match the experimental reports, but the relative relations between classes are retained. (*) Note that the ranges reported in this table are a rough approximation to the range of mean values reported in different studies (see below). Naturally, values like the maximum rate (νmax[Hz]) depend entirely on the range of input current considered in a given experiment, so in this case, only the relative ratio is pertinent. (PDF) [file pcbi.1006781.s002.pdf]

Parameter	Data (*)	Model $\bar{x}$	Model s	Description
I_{rh}[pA]	$[190, 250]$	262.73	45.88	rheobase current
	$[190, 250]$	251.5	44.8	
	$[80, 150]$	172.16	19.8	
	$E \approx I_1 >> I_2$	$E \approx I_1 >> I_2$		
Slope[Hz/pA]	$[140, 250]$	226.79	36.1	slope of the fI curve
	$[240, 500]$	529.46	252.16	
	$[150, 400]$	305.82	76.21	
	$I_1 > I_2 > E$	$I_1 > I_2 > E$		
ν_{min}[Hz]	$[0.5, 10]$	4.84	2.7	minimum firing rate
	$[15, 30]$	15.57	3.3	
	$[5, 20]$	8.23	3.42	
	$I_1 > I_2 > E$	$I_1 > I_2 > E$		
ν_{max}[Hz]	$[15, 80]$	130.83	21.31	maximum firing rate
	$[180, 250]$	330.28	164.93	
	$[40, 200]$	208.69	38.8	
	$I_1 > I_2 > E$	$I_1 > I_2 > E$		
