# Supplementary material for: Leveraging heterogeneity for neural computation with fading memory in layer 2/3 cortical microcircuits
Source: PLoS Comput Biol. 2019 Apr 25;15(4):e1006781. doi: 10.1371/journal.pcbi.1006781 (PMC6504118; doi:10.1371/journal.pcbi.1006781)
Supplement: S1 Appendix — List of the main references used to constrain model parameters. (PDF) [file pcbi.1006781.s003.pdf]

Leveraging heterogeneity for neural computation with fading memory in layer 2/3 cortical microcircuits

S1 Appendix - Primary data sources

Renato Duarte[1,2,3,4,*], Abigail Morrison[1,2,3,5]

1 Institute of Neuroscience and Medicine (INM-6), Institute for Advanced Simulation (IAS-6) and JARA Institute Brain Structure-Function Relationships (JBI-1 / INM-10), Jülich Research Centre, Jülich, Germany
2 Bernstein Center Freiburg, Albert-Ludwig University of Freiburg, Freiburg im Breisgau, Germany
3 Faculty of Biology, Albert-Ludwig University of Freiburg, Freiburg im Breisgau, Germany
4 Institute of Adaptive and Neural Computation, School of Informatics, University of Edinburgh, Edinburgh, UK
5 Institute of Cognitive Neuroscience, Faculty of Psychology, Ruhr-University Bochum, Bochum, Germany

* r.duarte@fz-juelich.de

Primary data sources

Primary literature sources considered and used to constrain and guide the choices for all the different parameter sets. Note that most of the sources for the neuronal parameters correspond to individual data points included in the NeuroElectro [1, 2] database. Additionally, it is worth mentioning that, apart from direct experimental measurements, we also included the values of some parameters used in previous biophysical and phenomenological models, provided they accounted for the relevant phenomena.

- Neurons:
  [3–19, 19–82, 82–92]
- Receptors:
  [67, 93–119]
- Synapses:
  [3, 5, 8, 16–18, 100, 120–125]
- Connectivity:
  [3, 5, 8, 10, 120, 121, 123–135]

References

1. Tripathy SJ, Savitskaya J, Burton SD, Urban NN, Gerkin RC. NeuroElectro: a window to the world's neuron electrophysiology data. Frontiers in Neuroinformatics. 2014;8(April):40. doi:10.3389/fninf.2014.00040.

2. Tripathy SJ, Burton SD, Geramita M, Gerkin RC, Urban NN. Brain-wide analysis of electrophysiological diversity yields novel categorization of mammalian neuron types. Journal of Neurophysiology. 2015;113(10):3474–3489. doi:10.1152/jn.00237.2015.

3. Avermann M, Tomm C, Mateo C, Gerstner W, Petersen CCH. Microcircuits of excitatory and inhibitory neurons in layer 2/3 of mouse barrel cortex. Journal of Neurophysiology. 2012;107(11):3116–3134. doi:10.1152/jn.00917.2011.

4. Harrison PM, Badel L, Wall MJ, Richardson MJE. Experimentally Verified Parameter Sets for Modelling Heterogeneous Neocortical Pyramidal-Cell Populations. PLoS Computational Biology. 2015;11(8):e1004165. doi:10.1371/journal.pcbi.1004165.

5. Lu Jt, Li Cy, Zhao JP, Poo Mm, Zhang Xh. Spike-Timing-Dependent Plasticity of Neocortical Excitatory Synapses on Inhibitory Interneurons Depends on Target Cell Type. Journal of Neuroscience. 2007;27(36):9711–9720. doi:10.1523/JNEUROSCI.2513-07.2007.

6. Neske GT, Patrick SL, Connors BW. Contributions of Diverse Excitatory and Inhibitory Neurons to Recurrent Network Activity in Cerebral Cortex. Journal of Neuroscience. 2015;35(3):1089–1105. doi:10.1523/JNEUROSCI.2279-14.2015.

7. Pi HJ, Hangya B, Kvitsiani D, Sanders JI, Huang ZJ, Kepecs A. Cortical interneurons that specialize in disinhibitory control. Nature. 2013;503(7477):521–524. doi:10.1038/nature12676.

8. Helmstaedter M, Sakmann B, Feldmeyer D. L2/3 Interneuron groups defined by multiparameter analysis of axonal projection, dendritic geometry, and electrical excitability. Cerebral Cortex. 2009;19(4):951–962. doi:10.1093/cercor/bhn130.

9. Kröner S, Krimer LS, Lewis DA, Barrionuevo G. Dopamine increases inhibition in the monkey dorsolateral prefrontal cortex through cell type-specific modulation of interneurons. Cerebral Cortex. 2007;17(5):1020–1032. doi:10.1093/cercor/bhl012.

10. Levy RB, Reyes AD. Spatial Profile of Excitatory and Inhibitory Synaptic Connectivity in Mouse Primary Auditory Cortex. Journal of Neuroscience. 2012;32(16):5609–5619. doi:10.1523/JNEUROSCI.5158-11.2012.

11. Tyler WA, Medalla M, Guillamon-Vivancos T, Luebke JI, Haydar TF. Neural Precursor Lineages Specify Distinct Neocortical Pyramidal Neuron Types. Journal of Neuroscience. 2015;35(15):6142–6152. doi:10.1523/JNEUROSCI.0335-15.2015.

12. Lacroix A, Toussay X, Anenberg E, Lecrux C, Ferreiros N, Karagiannis A, et al. COX-2-Derived Prostaglandin E2 Produced by Pyramidal Neurons Contributes to Neurovascular Coupling in the Rodent Cerebral Cortex. Journal of Neuroscience. 2015;35(34):11791–11810. doi:10.1523/JNEUROSCI.0651-15.2015.

13. Cruikshank SJ, Ahmed OJ, Stevens TR, Patrick SL, Gonzalez AN, Elmaleh M, et al. Thalamic Control of Layer 1 Circuits in Prefrontal Cortex. Journal of Neuroscience. 2012;32(49):17813–17823. doi:10.1523/JNEUROSCI.3231-12.2012.

14. Zaitsev AV, Povysheva NV, Gonzalez-Burgos G, Lewis DA. Electrophysiological classes of layer 2/3 pyramidal cells in monkey prefrontal cortex. Journal of Neurophysiology. 2012;108(2):595–609. doi:10.1152/jn.00859.2011.

15. van der Velden L, van Hooft JA, Chameau P. Altered dendritic complexity affects firing properties of cortical layer 2/3 pyramidal neurons in mice lacking the 5-HT3A receptor. Journal of Neurophysiology. 2012;108(5):1521–1528. doi:10.1152/jn.00829.2011.

16. Povysheva NV, Gonzalez-Burgos G, Zaitsev AV, Kr??ner S, Barrionuevo G, Lewis DA, et al. Properties of excitatory synaptic responses in fast-spiking interneurons and pyramidal cells from monkey and rat prefrontal cortex. Cerebral Cortex. 2006;16(4):541–552. doi:10.1093/cercor/bhj002.

17. Valdés-Sánchez L, Escámez T, Echevarria D, Ballesta JJ, Tabarés-Seisdedos R, Reiner O, et al. Postnatal alterations of the inhibitory synaptic responses recorded from cortical pyramidal neurons in the Lis1/sLis1 mutant mouse. Molecular and Cellular Neuroscience. 2007;35(2):220–229. doi:10.1016/j.mcn.2007.02.017.

18. Gonzalez-Burgos G, Miyamae T, Pafundo DE, Yoshino H, Rotaru DC, Hoftman G, et al. Functional maturation of GABA synapses during postnatal development of the monkey dorsolateral prefrontal cortex. Cerebral Cortex. 2015;25(11):4076–4093. doi:10.1093/cercor/bhu122.

19. González-Burgos G, Krimer LS, Urban NN, Barrionuevo G, Lewis DA. Synaptic Efficacy during Repetitive Activation of Excitatory Inputs in Primate Dorsolateral Prefrontal Cortex. Cerebral Cortex. 2004;14(5):530–542. doi:10.1093/cercor/bhh015.

20. Sippy T, Yuste R. Decorrelating Action of Inhibition in Neocortical Networks. Journal of Neuroscience. 2013;33(23):9813–9830. doi:10.1523/JNEUROSCI.4579-12.2013.

21. Pillai AG, Henckens MJAG, Fernández G, Joëls M. Delayed effects of corticosterone on slow after-hyperpolarization potentials in mouse hippocampal versus prefrontal cortical pyramidal neurons. PLoS ONE. 2014;9(6):e99208. doi:10.1371/journal.pone.0099208.

22. Dong H, Shao Z, Nerbonne JM, Burkhalter A. Differential depression of inhibitory synaptic responses in feedforward and feedback circuits between different areas of mouse visual cortex. Journal of Comparative Neurology. 2004;475(3):361–373. doi:10.1002/cne.20164.

23. Yang W, Carrasquillo Y, Hooks BM, Nerbonne JM, Burkhalter A. Distinct Balance of Excitation and Inhibition in an Interareal Feedforward and Feedback Circuit of Mouse Visual Cortex. Journal of Neuroscience. 2013;33(44):17373–17384. doi:10.1523/JNEUROSCI.2515-13.2013.

24. Sessler FM, Hsu FC, Felder TN, Zhai J, Lin RC, Wieland SJ, et al. Effects of ethanol on rat somatosensory cortical neurons. Brain research. 1998;804(2):266–74.

25. Lee JCF, Callaway JC, Foehring RC. Effects of Temperature on Calcium Transients and Ca 2+ -Dependent Afterhyperpolarizaions in Neocortical Pyramidal Neurons. Journal of Neurophysiology. 2005;93(4):2012–2020. doi:10.1152/jn.01017.2004.

26. Nowak LG. Electrophysiological Classes of Cat Primary Visual Cortical Neurons In Vivo as Revealed by Quantitative Analyses. Journal of Neurophysiology. 2002;89(3):1541–1566. doi:10.1152/jn.00580.2002.

27. Yang L, Benardo LS. Epileptogenesis following neocortical trauma from two sources of disinhibition. Journal of neurophysiology. 1997;78(5):2804–2810.

28. Guan D, Lee JCF, Higgs MH, Spain WJ, Foehring RC. Functional Roles of Kv1 Channels in Neocortical Pyramidal Neurons. Journal of Neurophysiology. 2007;97(3):1931–1940. doi:10.1152/jn.00933.2006.

29. Barnes SJ, Cheetham CE, Liu Y, Bennett SH, Albieri G, Jorstad AA, et al. Delayed and Temporally Imprecise Neurotransmission in Reorganizing Cortical Microcircuits. Journal of Neuroscience. 2015;35(24):9024–9037. doi:10.1523/JNEUROSCI.4583-14.2015.

30. Brill J, Huguenard JR. Enhanced infragranular and supragranular synaptic input onto layer 5 pyramidal neurons in a rat model of cortical dysplasia. Cerebral Cortex. 2010;20(12):2926–2938. doi:10.1093/cercor/bhq040.

31. Cummings DM, Andre VM, Uzgil BO, Gee SM, Fisher YE, Cepeda C, et al. Alterations in Cortical Excitation and Inhibition in Genetic Mouse Models of Huntington's Disease. Journal of Neuroscience. 2009;29(33):10371–10386. doi:10.1523/JNEUROSCI.1592-09.2009.

32. Elstrott J, Clancy KB, Jafri H, Akimenko I, Feldman DE. Cellular mechanisms for response heterogeneity among L2/3 pyramidal cells in whisker somatosensory cortex. Journal of Neurophysiology. 2014;112(2):233–248. doi:10.1152/jn.00848.2013.

33. Aramakis VB, Hsieh CY, Leslie FM, Metherate R. A critical period for nicotine-induced disruption of synaptic development in rat auditory cortex. The Journal of neuroscience : the official journal of the Society for Neuroscience. 2000;20(16):6106–6116. doi:20/16/6106 [pii].

34. Shruti S, Clem RL, Barth AL. A seizure-induced gain-of-function in BK channels is associated with elevated firing activity in neocortical pyramidal neurons. Neurobiology of Disease. 2008;30(3):323–330. doi:10.1016/j.nbd.2008.02.002.

35. Kobayashi M, Sasabe T, Shiohama Y, Koshikawa N. Activation of ??1-adrenoceptors increases firing frequency through protein kinase C in pyramidal neurons of rat visual cortex. Neuroscience Letters. 2008;430(2):175–180. doi:10.1016/j.neulet.2007.10.047.

36. Luebke JI, Medalla M, Amatrudo JM, Weaver CM, Crimins JL, Hunt B, et al. Age-related changes to layer 3 pyramidal cells in the rhesus monkey visual cortex. Cerebral Cortex. 2015;25(6):1454–1468. doi:10.1093/cercor/bht336.

37. Ren M, Cao V, Ye Y, Manji HK, Wang KH. Arc Regulates Experience-Dependent Persistent Firing Patterns in Frontal Cortex. Journal of Neuroscience. 2014;34(19):6583–6595. doi:10.1523/JNEUROSCI.0167-14.2014.

38. Waters J, Helmchen F. Background Synaptic Activity Is Sparse in Neocortex. Journal of Neuroscience. 2006;26(32):8267–8277. doi:10.1523/JNEUROSCI.2152-06.2006.

39. Tateno T. Threshold Firing Frequency-Current Relationships of Neurons in Rat Somatosensory Cortex: Type 1 and Type 2 Dynamics. Journal of Neurophysiology. 2004;92(4):2283–2294. doi:10.1152/jn.00109.2004.

40. Lemtiri-Chlieh F, Levine ES. Lack of Depolarization-Induced Suppression of Inhibition (DSI) in Layer 2/3 Interneurons That Receive Cannabinoid-Sensitive Inhibitory Inputs. Journal of Neurophysiology. 2007;98(5):2517–2524. doi:10.1152/jn.00817.2007.

41. Luhmann HJ, Karpuk N, Qü M, Zilles K, Brill J, Huguenard JR, et al. Characterization of Neuronal Migration Disorders in Neocortical Structures . II . Intracellular In Vitro Recordings Characterization of Neuronal Migration Disorders in Neocortical Structures . II . Intracellular In Vitro Recordings. Journal of neurophysiology. 1998;80(1):92–102.

42. Krimer LS. Cluster Analysis-Based Physiological Classification and Morphological Properties of Inhibitory Neurons in Layers 2-3 of Monkey Dorsolateral Prefrontal Cortex. Journal of Neurophysiology. 2005;94(5):3009–3022. doi:10.1152/jn.00156.2005.

43. Wayman WN, Chen L, Napier TC, Hu XT. Cocaine self-administration enhances excitatory responses of pyramidal neurons in the rat medial prefrontal cortex to human immunodeficiency virus-1 Tat. European Journal of Neuroscience. 2015;41(9):1195–1206. doi:10.1111/ejn.12853.

44. Andjelic S, Gallopin T, Cauli B, Hill EL, Roux L, Badr S, et al. Glutamatergic Nonpyramidal Neurons From Neocortical Layer VI and Their Comparison With Pyramidal and Spiny Stellate Neurons. Journal of Neurophysiology. 2008;101(2):641–654. doi:10.1152/jn.91094.2008.

45. Castro PA, Pleasure SJ, Baraban SC. Hippocampal heterotopia with molecular and electrophysiological properties of neocortical neurons. Neuroscience. 2002;114(4):961–972. doi:10.1016/S0306-4522(02)00296-8.

46. Amatrudo JM, Weaver CM, Crimins JL, Hof PR, Rosene DL, Luebke JI. Influence of Highly Distinctive Structural Properties on the Excitability of Pyramidal Neurons in Monkey Visual and Prefrontal Cortices. Journal of Neuroscience. 2012;32(40):13644–13660. doi:10.1523/JNEUROSCI.2581-12.2012.

47. Takei H, Fujita S, Shirakawa T, Koshikawa N, Kobayashi M. Insulin facilitates repetitive spike firing in rat insular cortex via phosphoinositide 3-kinase but not mitogen activated protein kinase cascade. Neuroscience. 2010;170(4):1199–1208. doi:10.1016/j.neuroscience.2010.07.061.

48. Huggenberger S, Vater M, Deisz RA. Interlaminar differences of intrinsic properties of pyramidal neurons in the auditory cortex of mice. Cerebral Cortex. 2009;19(5):1008–1018. doi:10.1093/cercor/bhn143.

49. Tanaka YR, Tanaka YH, Konno M, Fujiyama F, Sonomura T, Okamoto-Furuta K, et al. Local Connections of Excitatory Neurons to Corticothalamic Neurons in the Rat Barrel Cortex. Journal of Neuroscience. 2011;31(50):18223–18236. doi:10.1523/JNEUROSCI.3139-11.2011.

50. Gulledge AT, Bucci DJ, Zhang SS, Matsui M, Yeh HH. M1 Receptors Mediate Cholinergic Modulation of Excitability in Neocortical Pyramidal Neurons. Journal of Neuroscience. 2009;29(31):9888–9902. doi:10.1523/JNEUROSCI.1366-09.2009.

51. Oswald AMM, Reyes AD. Maturation of Intrinsic and Synaptic Properties of Layer 2/3 Pyramidal Neurons in Mouse Auditory Cortex. Journal of Neurophysiology. 2008;99(6):2998–3008. doi:10.1152/jn.01160.2007.

52. Van Aerde KI, Feldmeyer D. Morphological and physiological characterization of pyramidal neuron subtypes in rat medial prefrontal cortex. Cerebral Cortex. 2015;25(3):788–805. doi:10.1093/cercor/bht278.

53. Sutor B, Schmolke C, Teubner B, Schirmer C, Willecke K. Myelination Defects and Neuronal Hyperexcitability in the Neocortex of Connexin 32-deficient Mice. Cerebral Cortex. 2000;10(7):684–697. doi:10.1093/cercor/10.7.684.

54. Hadjilambreva G. Neuromodulation by a Cytokine: Interferon- Differentially Augments Neocortical Neuronal Activity and Excitability. Journal of Neurophysiology. 2004;93(2):843–852. doi:10.1152/jn.01224.2003.

55. Beggs JM, Moyer JR, McGann JP, Brown TH. Prolonged synaptic integration in perirhinal cortical neurons. Journal of neurophysiology. 2000;83(6):3294–3298.

56. Abel HJ. Relationships Between Intracellular Calcium and Afterhyperpolarizations in Neocortical Pyramidal Neurons. Journal of Neurophysiology. 2003;91(1):324–335. doi:10.1152/jn.00583.2003.

57. Cheetham CEJ, Hammond MSL, Edwards CEJ, Finnerty GT. Sensory Experience Alters Cortical Connectivity and Synaptic Function Site Specifically. Journal of Neuroscience. 2007;27(13):3456–3465. doi:10.1523/JNEUROSCI.5143-06.2007.

58. Lambe EK, Goldman-Rakic PS, Aghajanian GK. Serotonin induces EPSCs preferentially in layer V pyramidal neurons of the frontal cortex in the rat. Cerebral cortex (New York, NY : 1991). 2000;10(10):974–980. doi:10.1093/cercor/10.10.974.

59. Hirai Y, Morishima M, Karube F, Kawaguchi Y. Specialized Cortical Subnetworks Differentially Connect Frontal Cortex to Parahippocampal Areas. Journal of Neuroscience. 2012;32(5):1898–1913. doi:10.1523/JNEUROSCI.2810-11.2012.

60. Chen IW, Helmchen F, Lutcke H. Specific Early and Late Oddball-Evoked Responses in Excitatory and Inhibitory Neurons of Mouse Auditory Cortex. Journal of Neuroscience. 2015;35(36):12560–12573. doi:10.1523/JNEUROSCI.2240-15.2015.

61. Pineda JC, Waters RS, Foehring RC. Specificity in the interaction of HVA Ca2+ channel types with Ca2+-dependent AHPs and firing behavior in neocortical pyramidal neurons. J Neurophysiol. 1998;79(5):2522–2534.

62. Salling MC, Harrison NL, Abernathy K, Chandler L, Woodward J, Amatrudo J, et al. Strychnine-sensitive glycine receptors on pyramidal neurons in layers II/III of the mouse prefrontal cortex are tonically activated. Journal of neurophysiology. 2014;112(5):1169–78. doi:10.1152/jn.00714.2013.

63. Cho Kh, Jang JH, Jang Hj, Kim Mj, Hee S, Fukuda T, et al. Subtype-Specific Dendritic Calcium Dynamics of Inhibitory Interneurons in the Rat Visual Cortex. Journal of Neurophysiology. 2010;104(June 2010):840–853. doi:10.1152/jn.00146.2010.

64. Lee CM, Chang WC, Chang KB, Shyu BC. Synaptic organization and input-specific short-term plasticity in anterior cingulate cortical neurons with intact thalamic inputs. European Journal of Neuroscience. 2007;25(9):2847–2861. doi:10.1111/j.1460-9568.2007.05485.x.

65. Mowery TM, Kotak VC, Sanes DH. Transient Hearing Loss Within a Critical Period Causes Persistent Changes to Cellular Properties in Adult Auditory Cortex. Cerebral Cortex. 2015;25(8):2083–2094. doi:10.1093/cercor/bhu013.

66. Telfeian AE, Connors BW. Widely integrative properties of layer 5 pyramidal cells support a role for processing of extralaminar synaptic inputs in rat neocortex. Neuroscience Letters. 2003;343(2):121–124. doi:10.1016/S0304-3940(03)00379-3.

67. Wang HX, Gao WJ. Development of calcium-permeable AMPA receptors and their correlation with NMDA receptors in fast-spiking interneurons of rat prefrontal cortex. The Journal of Physiology. 2010;588(15):2823–2838. doi:10.1113/jphysiol.2010.187591.

68. Blatow M, Rozov A, Katona I, Hormuzdi SG, Meyer AH, Whittington MA, et al. A novel network of multipolar bursting interneurons generates theta frequency oscillations in neocortex. Neuron. 2003;38(5):805–817. doi:10.1016/S0896-6273(03)00300-3.

69. Takesian AE, Kotak VC, Sanes DH. Age-dependent effect of hearing loss on cortical inhibitory synapse function. Journal of Neurophysiology. 2012;107(3):937–947. doi:10.1152/jn.00515.2011.

70. Zhou FW, Roper SN. Altered firing rates and patterns in interneurons in experimental cortical Dysplasia. Cerebral Cortex. 2011;21(7):1645–1658. doi:10.1093/cercor/bhq234.

71. Rose HJ. Auditory Thalamocortical Transmission Is Reliable and Temporally Precise. Journal of Neurophysiology. 2005;94(3):2019–2030. doi:10.1152/jn.00860.2004.

72. Aerde KIV, Qi G, Feldmeyer D. Cell type-specific effects of adenosine on cortical neurons. Cerebral Cortex. 2015;25(3):772–787. doi:10.1093/cercor/bht274.

73. Schiff ML, Reyes AD. Characterization of thalamocortical responses of regular-spiking and fast-spiking neurons of the mouse auditory cortex in vitro and in silico. Journal of Neurophysiology. 2012;107(5):1476–1488. doi:10.1152/jn.00208.2011.

74. Yang JM, Zhang J, Chen XJ, Geng HY, Ye M, Spitzer NC, et al. Development of GABA Circuitry of Fast-Spiking Basket Interneurons in the Medial Prefrontal Cortex of erbb4-Mutant Mice. Journal of Neuroscience. 2013;33(50):19724–19733. doi:10.1523/JNEUROSCI.1584-13.2013.

75. Oswald AMM, Reyes AD. Development of inhibitory timescales in auditory cortex. Cerebral Cortex. 2011;21(6):1351–1361. doi:10.1093/cercor/bhq214.

76. Zhong P, Yan Z. Distinct Physiological Effects of Dopamine D4 Receptors on Prefrontal Cortical Pyramidal Neurons and Fast-Spiking Interneurons. Cerebral Cortex. 2016;26(1):180–191. doi:10.1093/cercor/bhu190.

77. Gao WJ, Wang Y, Goldman-Rakic PS. Dopamine modulation of perisomatic and peridendritic inhibition in prefrontal cortex. J Neurosci. 2003;23(5):1622–1630. doi:23/5/1622 [pii].

78. Imbrosci B, Neitz A, Mittmann T. Focal cortical lesions induce bidirectional changes in the excitability of fast spiking and non fast spiking cortical interneurons. PLoS ONE. 2014;9(10):e111105. doi:10.1371/journal.pone.0111105.

79. Erisir A, Lau D, Rudy B, Leonard CS. Function of Specific K+ Channels in Sustained High-Frequency Firing of Fast-Spiking Neocortical Interneurons. J Neurophysiol. 1999;82(5):2476–2489.

80. Gonzalez-Burgos G. Functional Properties of Fast Spiking Interneurons and Their Synaptic Connections With Pyramidal Cells in Primate Dorsolateral Prefrontal Cortex. Journal of Neurophysiology. 2004;93(2):942–953. doi:10.1152/jn.00787.2004.

81. Karayannis T, Huerta-Ocampo I, Capogna M. GABAergic and pyramidal neurons of deep cortical layers directly receive and differently integrate callosal input. Cerebral Cortex. 2007;17(5):1213–1226. doi:10.1093/cercor/bhl035.

82. Tateno T, Robinson HPC. Integration of Broadband Conductance Input in Rat Somatosensory Cortical Inhibitory Interneurons: An Inhibition-Controlled Switch Between Intrinsic and Input-Driven Spiking in Fast-Spiking Cells. Journal of Neurophysiology. 2008;101(2):1056–1072. doi:10.1152/jn.91057.2008.

83. Gorelova N. Mechanisms of Dopamine Activation of Fast-Spiking Interneurons That Exert Inhibition in Rat Prefrontal Cortex. Journal of Neurophysiology. 2002;88(6):3150–3166. doi:10.1152/jn.00335.2002.

84. Zaitsev AV, Povysheva NV, Gonzalez-Burgos G, Rotaru D, Fish KN, Krimer LS, et al. Interneuron diversity in layers 2-3 of monkey prefrontal cortex. Cerebral Cortex. 2009;19(7):1597–1615. doi:10.1093/cercor/bhn198.

85. Dougherty SE, Bartley AF, Lucas EK, Hablitz JJ, Dobrunz LE, Cowell RM. Mice lacking the transcriptional coactivator PGC-1α exhibit alterations in inhibitory synaptic transmission in the motor cortex. Neuroscience. 2014;271:137–148. doi:10.1016/j.neuroscience.2014.04.023.

86. Abe Y, Namba H, Kato T, Iwakura Y, Nawa H. Neuregulin-1 signals from the periphery regulate AMPA receptor sensitivity and expression in GABAergic interneurons in developing neocortex. The Journal of neuroscience : the official journal of the Society for Neuroscience. 2011;31(15):5699–709. doi:10.1523/JNEUROSCI.3477-10.2011.

87. Kawaguchi Y, Kubota Y. Neurochemical features and synaptic connections of large physiologically-identified GABAergic cells in the rat frontal cortex. Neuroscience. 1998;85(3):677–701. doi:10.1016/S0306-4522(97)00685-4.

88. Povysheva NV, Zaitsev AV, Rotaru DC, Gonzalez-Burgos G, Lewis DA, Krimer LS. Parvalbumin-positive basket interneurons in monkey and rat prefrontal cortex. Journal of neurophysiology. 2008;100(4):2348–60. doi:10.1152/jn.90396.2008.

89. Miyoshi G, Butt SJB, Takebayashi H, Fishell G. Physiologically Distinct Temporal Cohorts of Cortical Interneurons Arise from Telencephalic Olig2-Expressing Precursors. Journal of Neuroscience. 2007;27(29):7786–7798. doi:10.1523/JNEUROSCI.1807-07.2007.

90. Akgul G, Wollmuth LP. Synapse-Associated Protein 97 Regulates the Membrane Properties of Fast-Spiking Parvalbumin Interneurons in the Visual Cortex. Journal of Neuroscience. 2013;33(31):12739–12750. doi:10.1523/JNEUROSCI.0040-13.2013.

91. Chadderton P, Agapiou JP, McAlpine D, Margrie TW. The Synaptic Representation of Sound Source Location in Auditory Cortex. Journal of Neuroscience. 2009;29(45):14127–14135. doi:10.1523/JNEUROSCI.2061-09.2009.

92. Szabadics J. Excitatory Effect of GABAergic Axo-Axonic Cells in Cortical Microcircuits. Science. 2006;311(5758):233–235. doi:10.1126/science.1121325.

93. Destexhe A, Mainen ZF, Sejnowski TJ. Synthesis of models for excitable membranes, synaptic transmission and neuromodulation using a common kinetic formalism. Journal of Computational Neuroscience. 1994;1(3):195–230. doi:10.1007/BF00961734.

94. Destexhe A, Mainen ZF, Sejnowski TJ. Kinetic models of synaptic transmission. In: Koch C, Segev I, editors. Methods in Neuronal Modeling. 2nd ed. Cambridge, MA: MIT Press; 1998. p. 1–25. Available from: http://cns.iaf.cnrs-gif.fr/abstracts/KSchap96.html.

95. Hestrin S. Different glutamate receptor channels mediate fast excitatory synaptic currents in inhibitory and excitatory cortical neurons. Neuron. 1993;11(6):1083–1091. doi:10.1016/0896-6273(93)90221-C.

96. McCormick DA, Wang Z, Huguenard J. Neurotransmitter control of neocortical neuronal activity and excitability. Cerebral Cortex. 1993;3(5):387–398. doi:10.1093/cercor/3.5.387.

97. Angulo MC, Rossier J, Audinat E. Postsynaptic glutamate receptors and integrative properties of fast-spiking interneurons in the rat neocortex. Journal of neurophysiology. 1999;82(3):1295–1302.

98. Myme CIO. The NMDA-to-AMPA Ratio at Synapses Onto Layer 2/3 Pyramidal Neurons Is Conserved Across Prefrontal and Visual Cortices. Journal of Neurophysiology. 2003;90(2):771–779. doi:10.1152/jn.00070.2003.

99. Watt AJ, Van Rossum MCW, MacLeod KM, Nelson SB, Turrigiano GG. Activity coregulates quantal AMPA and NMDA currents at neocortical synapses. Neuron. 2000;26(3):659–670. doi:10.1016/S0896-6273(00)81202-7.

100. Hoffmann JHO, Meyer HS, Schmitt AC, Straehle J, Weitbrecht T, Sakmann B, et al. Synaptic conductance estimates of the connection between local inhibitor interneurons and pyramidal neurons in layer 2/3 of a cortical column. Cerebral Cortex. 2015;25(11):4415–4429. doi:10.1093/cercor/bhv039.

101. Connors BW, Malenka RC, Silva LR. Two inhibitory postsynaptic potentials, and GABAA and GABAB receptor-mediated responses in neocortex of rat and cat. The Journal of Physiology. 1988;406(1):443–468. doi:10.1113/jphysiol.1988.sp017390.

102. Hill E, Kalloniatis M, Tan SS. Glutamate, GABA and precursor amino acids in adult mouse neocortex: cellular diversity revealed by quantitative immunocytochemistry. Cerebral cortex (New York, NY : 1991). 2000;10(11):1132–42.

103. Paoletti P, Bellone C, Zhou Q. NMDA receptor subunit diversity: impact on receptor properties, synaptic plasticity and disease. Nature Reviews Neuroscience. 2013;14(6):383–400. doi:10.1038/nrn3504.

104. Jahr CE, Stevens CF. Voltage dependence of NMDA-activated macroscopic conductances predicted by single-channel kinetics. The Journal of neuroscience. 1990;10(9):3178–3182. doi:1990/09/01 00:01.

105. Hestrin S, Sah P, Nicoll RA. Mechanisms generating the time course of dual component excitatory synaptic currents recorded in hippocampal slices. Neuron. 1990;5(3):247–253. doi:10.1016/0896-6273(90)90162-9.

106. Moreau AW, Kullmann DM. NMDA receptor-dependent function and plasticity in inhibitory circuits. Neuropharmacology. 2013;74:23–31. doi:10.1016/j.neuropharm.2013.03.004.

107. Nissen W, Szabo A, Somogyi J, Somogyi P, Lamsa KP. Cell Type-Specific Long-Term Plasticity at Glutamatergic Synapses onto Hippocampal Interneurons Expressing either Parvalbumin or CB1 Cannabinoid Receptor. Journal of Neuroscience. 2010;30(4):1337–1347. doi:10.1523/JNEUROSCI.3481-09.2010.

108. Izhikevich EM, Gally JA, Edelman GM. Spike-timing dynamics of neuronal groups. Cerebral Cortex. 2004;14(8):933–944. doi:10.1093/cercor/bhh053.

109. Izhikevich EM, Edelman GM. Large-scale model of mammalian thalamocortical systems. Proceedings of the National Academy of Sciences. 2008;105(9):3593–3598. doi:10.1073/pnas.0712231105.

110. Dayan P, Abbott LF. Theoretical Neuroscience: Computational and Mathematical Modeling of Neural Systems. vol. 15. Cambridge, MA: The MIT Press; 2001. Available from: https://books.google.de/books/about/Theoretical{_}Neuroscience.html?id=5GSKQgAACAAJ{&}pgis=1.

111. Gerstner W, Kistler WM. Spiking Neuron Models. Cambridge University Press; 2002. Available from: http://ebooks.cambridge.org/ref/id/CBO9780511815706.

112. Gerstner W, Kistler WM, Naud R, Paninski L. Neuronal Dynamics - from single neurons to networks and models of cognition. Cambridge University Press; 2014. Available from: https://books.google.de/books?id=D4j2AwAAQBAJ.

113. Brunel N, Wang XJ. Effects of neuromodulation in a cortical network model of object working memory dominated by recurrent inhibition. Journal of Computational Neuroscience. 2001;11(1):63–85. doi:10.1023/A:1011204814320.

114. Destexhe A, Sejnowski TJ. G protein activation kinetics and spillover of gamma-aminobutyric acid may account for differences between inhibitory responses in the hippocampus and thalamus. Proceedings of the National Academy of Sciences. 1995;92(21):9515–9519. doi:10.1073/pnas.92.21.9515.

115. Mason a, Nicoll A, Stratford K. Synaptic transmission between individual pyramidal neurons of the rat visual cortex in vitro. The Journal of Neuroscience. 1991;11(January):72–84.

116. Southan C, Sharman JL, Benson HE, Faccenda E, Pawson AJ, Alexander SPH, et al. The IUPHAR/BPS Guide to PHARMACOLOGY in 2016: Towards curated quantitative interactions between 1300 protein targets and 6000 ligands. Nucleic Acids Research. 2016;44(D1):D1054–D1068. doi:10.1093/nar/gkv1037.

117. Sah P, Hestrin S, Nicoll Ra. Properties of excitatory postsynaptic currents recorded in vitro from rat hippocampal interneurones. The Journal of physiology. 1990;430:605–616. doi:10.1113/jphysiol.1990.sp018310.

118. Uzun S, Kozumplik O, Jakovljević M, Sedić B. Side effects of treatment with benzodiazepines. Psychiatria Danubina. 2010;22(1):90–93. doi:10.1016/S0959-8049(02)81277-1.

119. Köhr G, De Koninck Y, Mody I. Properties of NMDA receptor channels in neurons acutely isolated from epileptic (kindled) rats. The Journal of neuroscience : the official journal of the Society for Neuroscience. 1993;13(8):3612–3627.

120. Feldmeyer D, Lübke J, Sakmann B. Efficacy and connectivity of intracolumnar pairs of layer 2/3 pyramidal cells in the barrel cortex of juvenile rats. The Journal of Physiology. 2006;575(2):583–602. doi:10.1113/jphysiol.2006.105106.

121. Thomson AM. Synaptic Connections and Small Circuits Involving Excitatory and Inhibitory Neurons in Layers 2-5 of Adult Rat and Cat Neocortex: Triple Intracellular Recordings and Biocytin Labelling In Vitro. Cerebral Cortex. 2002;12(9):936–953. doi:10.1093/cercor/12.9.936.

122. Monier C, Fournier J, Frégnac Y. In vitro and in vivo measures of evoked excitatory and inhibitory conductance dynamics in sensory cortices. Journal of Neuroscience Methods. 2008;169(2):323–365. doi:10.1016/j.jneumeth.2007.11.008.

123. Kubota Y, Kondo S, Nomura M, Hatada S, Yamaguchi N, Mohamed AA, et al. Functional effects of distinct innervation styles of pyramidal cells by fast spiking cortical interneurons. eLife. 2015;4(July 2015):1–27. doi:10.7554/eLife.07919.

124. Lefort S, Tomm C, Floyd Sarria JC, Petersen CCH. The Excitatory Neuronal Network of the C2 Barrel Column in Mouse Primary Somatosensory Cortex. Neuron. 2009;61(2):301–316. doi:10.1016/j.neuron.2008.12.020.

125. Helmstaedter M, Staiger JF, Sakmann B, Feldmeyer D. Efficient Recruitment of Layer 2/3 Interneurons by Layer 4 Input in Single Columns of Rat Somatosensory Cortex. Journal of Neuroscience. 2008;28(33):8273–8284. doi:10.1523/JNEUROSCI.5701-07.2008.

126. Tomm C. Analysing Neuronal Network Architectures: From Weight Distributions to Structure and Back. ÉCOLE POLYTECHNIQUE FÉDÉRALE DE LAUSANNE; 2012. Available from: https://infoscience.epfl.ch/record/174669/files/EPFL{_}TH5302.pdf.

127. Tomm C, Avermann M, Petersen C, Gerstner W, Vogels TP. Connection-type-specific biases make uniform random network models consistent with cortical recordings. Journal of Neurophysiology. 2014;112(8):1801–1814. doi:10.1152/jn.00629.2013.

128. Koulakov AA, Hromadka T, Zador AM. Correlated Connectivity and the Distribution of Firing Rates in the Neocortex. Journal of Neuroscience. 2009;29(12):3685–3694. doi:10.1523/JNEUROSCI.4500-08.2009.

129. Fino E, Yuste R. Dense inhibitory connectivity in neocortex. Neuron. 2011;69(6):1188–1203. doi:10.1016/j.neuron.2011.02.025.

130. Song S, Sj??str??m PJ, Reigl M, Nelson S, Chklovskii DB. Highly nonrandom features of synaptic connectivity in local cortical circuits. PLoS Biology. 2005;3(3):0507–0519. doi:10.1371/journal.pbio.0030068.

131. Perin R, Berger TK, Markram H. A synaptic organizing principle for cortical neuronal groups. Proceedings of the National Academy of Sciences. 2011;108(13):5419–5424. doi:10.1073/pnas.1016051108.

132. Yoshimura Y, Dantzker JLM, Callaway EM. Excitatory cortical neurons form fine-scale functional networks. Nature. 2005;433(7028):868–873. doi:10.1038/nature03252.

133. Yoshimura Y, Callaway EM. Fine-scale specificity of cortical networks depends on inhibitory cell type and connectivity. Nature Neuroscience. 2005;8(11):1552–1559. doi:10.1038/nn1565.

134. Potjans TC, Diesmann M. The cell-type specific cortical microcircuit: Relating structure and activity in a full-scale spiking network model. Cerebral Cortex. 2014;24(3):785–806. doi:10.1093/cercor/bhs358.

135. Hagen E, Dahmen D, Stavrinou ML, Lindén H, Tetzlaff T, Van Albada SJ, et al. Hybrid scheme for modeling local field potentials from point-neuron networks. Cerebral Cortex. 2016;26(12):4461–4496. doi:10.1093/cercor/bhw237.
