# Supplementary material for: Leveraging heterogeneity for neural computation with fading memory in layer 2/3 cortical microcircuits
Source: PLoS Comput Biol. 2019 Apr 25;15(4):e1006781. doi: 10.1371/journal.pcbi.1006781 (PMC6504118; doi:10.1371/journal.pcbi.1006781)
Supplement: S2 Appendix — (PDF) [file pcbi.1006781.s004.pdf]

Leveraging heterogeneity for neural computation with fading memory in layer 2/3 cortical microcircuits

S2 Appendix - Reproducibility and Replication

Renato Duarte[1,2,3,4,*], Abigail Morrison[1,2,3,5]

1 Institute of Neuroscience and Medicine (INM-6), Institute for Advanced Simulation (IAS-6) and JARA Institute Brain Structure-Function Relationships (JBI-1 / INM-10), Jülich Research Centre, Jülich, Germany
2 Bernstein Center Freiburg, Albert-Ludwig University of Freiburg, Freiburg im Breisgau, Germany
3 Faculty of Biology, Albert-Ludwig University of Freiburg, Freiburg im Breisgau, Germany
4 Institute of Adaptive and Neural Computation, School of Informatics, University of Edinburgh, Edinburgh, UK
5 Institute of Cognitive Neuroscience, Faculty of Psychology, Ruhr-University Bochum, Bochum, Germany

* r.duarte@fz-juelich.de

Reproducibility and Replication

Considerable efforts were taken to ensure transparency, openness and reproducibility. We provide all the relevant data, materials and code, necessary to replicate and scrutinize all the results presented in this manuscript.

OSF Repository

Through the Open Science Framework (osf.io), an open-access, curated and registered repository is available at [1]. The project contains all the relevant information necessary to replicate and scrutinize the present work, divided into the following components:

- Data - linked to the Sciebo campus cloud [2], where the data is hosted. Due to the large size of the data (totalling $\approx 550GB$), we do not provide a complete data package as a supplement to the manuscript. If there are any difficulties accessing the data through this OSF component, please contact the authors.
- Figures - all the figures in the manuscript
- Presentations - public presentations of the project
- Software - all dedicated and modified software that is necessary to run and replicate the experiments (see below):
  - NEST 2.10.0 modified
  - NMSAT v0.1
  - Project-specific Code (also provided in S1 Files)
- Manuscript - link to the main manuscript files
- Bibliography - linked to a database containing all the references used in this manuscript

Software and source code

The code package provided as a supplement (S1 File, also available in the OSF repository) implements project-specific functionality to NMSAT [3], which is a tailor-made python package that provides a generic set of tools to build, simulate and analyse neuronal microcircuit models with any degree of complexity, as exemplified in this study. It provides a high-level wrapper for PyNEST (used as the core simulation engine). The specificities of this project require the installation and use of a specific, modified version of NEST 2.10.0 [4] (available in the Software component of OSF or upon request), since it relies on 2 models that are not currently available in the main release (`iaf_cond_mtime`: neuron model with complex synaptic kinetics, `multiport_synapse`: connection model allowing the spike-triggered conductances onto the different receptors of a given synapse type). To use the provided software:

1. Setup - After ensuring that all dependencies are satisfied, NMSAT (https://github.com/rcfduarte/nmsat) needs to be downloaded and setup, as explained in the provided documentation (https://rcfduarte.github.io/nmsat/).
2. Project code - The code package for this project should then be extracted onto the `projects/` folder. The provided code has the following structure:

```
heterogeneity_project/
├── parameters/
│   └── preset/
├── computations/
└── scripts/
```

```
└── read_data/
```

where `read_data` contains all the analysis scripts necessary to read, analyse and plot the data (see Table B on page 5); `scripts` contains all the main experiments as a complete script, mostly for debugging purposes. The main simulations are run using combinations of parameters files with the corresponding computation function (see Table A on page 3 for a description of the experiments provided and the standard use case in the code documentation (https://rcfduarte.github.io/nmsat/) for instructions).

3. Running a simulation - Specific experiments can be run from scratch using the provided code. Modify the specific parameters as desired (paying particular attention to the system specificities) and execute the experiment:

```
$ python main.py -f {parameters_file} -c {computation} --extra {computation_parameters}
```

4. Replicating an experiment - Alternatively, experiments can be re-run using the original parameter file (`{data_label}_ParameterSpace.py` file, see Table B on page 5) when executing the experiment. Simply execute as above, replacing `{parameters_file}` with the full path to the stored parameters. Note, however, that the system parameters need to be edited in these cases, as they were executed on different machines.

Experiment	Parameters file	Computation
Single neuron fI curves and related parameters and distributions (Fig.1)	`single_neuron_fI`	`singleneuron_dcinput`
Single receptor kinetics (Fig. 2b)	`synaptic_response_receptors`	`synaptic_response`
PSP/PSC kinetics (Fig. 2c, d)	`synaptic_response_rest`	`synaptic_response`
Connectivity and degree distributions (Fig. 3) and illustrations of population activity (Fig. 4d (background) and Fig. 5c,d)	`noise_driven_dynamics`	`illustrate_activity(*)`
Population rate transfer functions and responses to background noise (Fig. 4 and Supp. Fig. 1)	`noise_driven_dynamics`	`noisedriven_dynamics`
State transitions and statistics of active states (Fig. 5a and b, Fig. 6 and Supp. Fig. 3)	`state_transition`	`characterize_state_transition`
Stimulus parameters, memory and capacity (Fig. 7b and c, Fig. 8, 9 and Supp. Fig. 4)	`stimulus_driven`	`measure_capacity`
Capacity analysis on pre-stored data	`stimulus_driven`	`measure_capacity_offline`

Table A. Summary of all the numerical experiments that can be run using the provided source code. Some are very memory and especially time-consuming (the capacity analysis, in particular). The item marked with (*) can be found in the scripts folder.

Datasets

Due to its total size and the fact that any given data point can be entirely reproduced with the provided code, the full data is only accessible through the OSF repository. We provide all the datasets necessary to replicate the main experiments, as well as the original parameters used for each simulation and the main results. The datasets are organized as follows:

```
data
├── neuron_parameters
├── receptor_parameters
├── synapse_parameters
├── population_RTF
├── state_transitions
├── active_state
├── input_tuning
└── capacity
    ├── data_label_ParameterSpace.py
    └── data_label/
        ├── Activity/
        ├── Figures/
        ├── Inputs/
        ├── Parameters/
        ├── Results/
        └── Output/
```

The specific sub-folder structure varies depending on the dataset, but typically follows the organization described above for `capacity/`, used as an example. For the `data_label` of specific experiments consult Table B on page 5. The code package we provide as supplement contains all the analysis scripts (`read_data` folder).

Description	Data label	Analysis script
Single neuron fI curves and distributions (Fig. 1)	single_neuron_dcinput	neuron_fI
Adaptation parameters for the different neuron classes $(a, b$, data not shown)	single_neuron_excitability	singleneuron_excitability
Receptor conductance kinetics (data not shown)	*neuron_rec*	receptor_conductances
Distribution of PSP amplitudes and latencies (Fig. 2d)	*syn*_heterogeneous	plot_het_synapse
Population rate transfer functions (Fig. 4a, b, c and Supp. Fig. 1)	populationRTF_normal_*het*	populationRTF_spiking
Sub-threshold responses to background noise (Fig. 4d and Fig. 5a, b)	populationRTF_normal_*het*	populationRTF_subthreshold
State transitions and characterization of quiet and active states, single trial (Fig. 4e and 7a)	state_transitions_fixed_rate_*het*	plot_state_transitions
Spiking statistics in the active state (Fig. 9 and Supp. Fig. 3)	active_state_*het*	active_state_spiking
Input amplitude tuning (Supp. Fig. 2)	stimulus_tuning_*het*	stimulus_amplitude
Stimulus resolution (Supp. Fig. 4)	stimulus_resolution_*het*	stimulus_resolution
Memory capacity (Fig. 7b and c)	capacity_*het*	memory_capacity
Total processing capacity (Fig. 9)	capacity_*het*	total_capacity
Receptor composition and memory capacity (Fig. 8)	receptor_ratios_*het*	capacity_parameter_scan

Table B. Summary of all datasets and corresponding scripts to analyse and plot the data. Note: For legacy reasons, the conditions are labelled as: HOM (homogeneous), HET1 (structural), HET2 (neuronal), HET3 (synaptic), HET or HETall (fully heterogeneous).

References

1. Duarte R, Seeholzer A, Morrison A. Leveraging heterogeneity for neural computations with fading memory. Open Science Framework. 2017;.

2. Vogl R, Rudolph D, Angenent H, Thoring A, Schild C, Stieglitz S, et al. sciebo - the Campuscloud: a Sync and Share Cloud Storage Service for the Academic and Research Community in North Rhine-Westphalia. PIK - Praxis der Informationsverarbeitung und Kommunikation. 2015;38(3-4):105–112. doi:doi:10.1515/pik-2015-0007.

3. Duarte R, Zajzon B, Morrison A. Neural Microcircuit Simulation And Analysis Toolkit. Zenodo. 2017;doi:10.5281/ZENODO.582645.

4. Bos H, Morrison, Abigail Peyser, Alexander Hahne J, Helias M, Kunkel S, Ippen T, Eppler JM, et al. Nest 2.10.0. 2015; p. DOI: 10.5281/ZENODO.44222. doi:10.5281/ZENODO.44222.
