# Supplementary figures and images for: Leveraging heterogeneity for neural computation with fading memory in layer 2/3 cortical microcircuits
Source: PLoS Comput Biol. 2019 Apr 25;15(4):e1006781. doi: 10.1371/journal.pcbi.1006781 (PMC6504118; doi:10.1371/journal.pcbi.1006781)

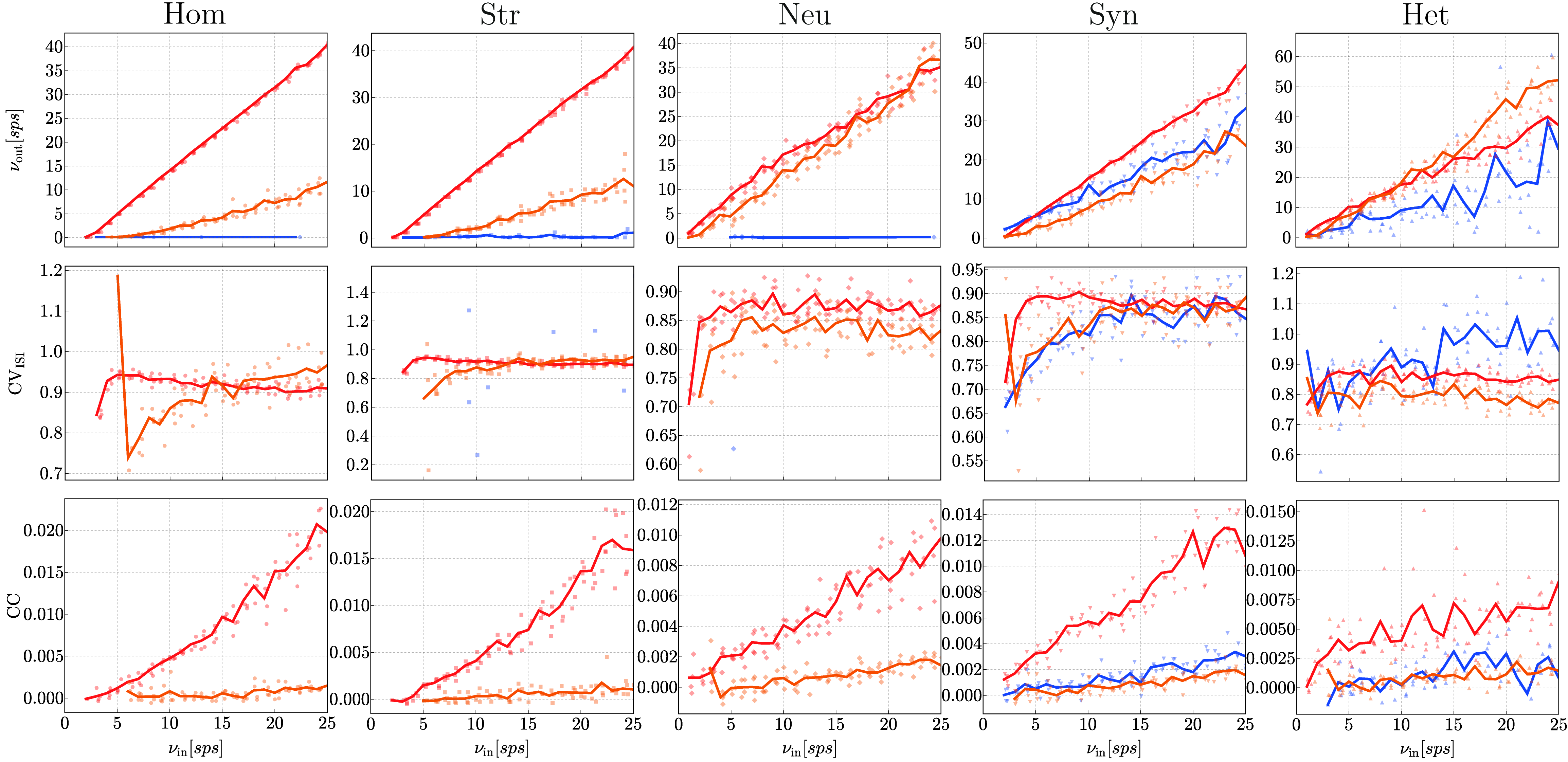

Supplement: S1 Fig — Characteristics of population spiking activity in response to background, Poissonian input (quiet state) in the various conditions analysed and for the 3 different population types (E, blue; I1, red; I2, orange), as a function of the input rate νin for a total simulation time of 10 seconds. The top row corresponds the population rate transfer functions, showing that E neurons fire extremely sparsely and synaptic heterogeneity is strictly required to obtain an active E population. The middle and bottom row depict the measured irregularity (CVISI) and synchrony (CC) in all conditions analysed. Note that in many conditions the spiking activity in the E population is so sparse that it is not possible to compute these metrics, since the total number of spikes is insufficient. (TIF) [file pcbi.1006781.s005.tif]

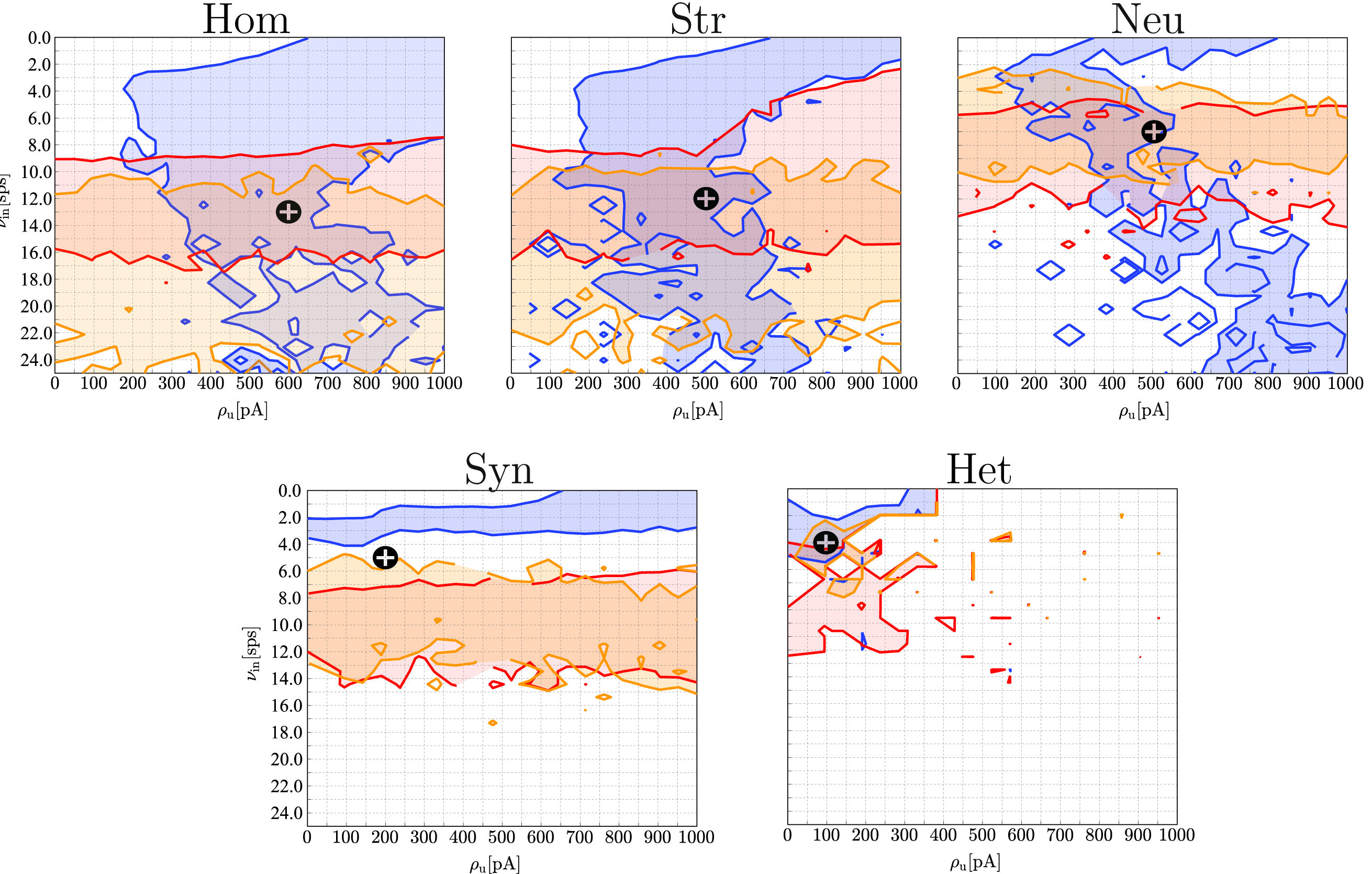

Supplement: S2 Fig — To emulate an active processing condition, an extra input current of maximum amplitude ρu is given to a randomly chosen subset of 25% excitatory neurons. The circuits in the different conditions exhibit different degrees of sensitivity to their inputs. To achieve adequate and comparable responses, we attempt to find combination of input parameters that allows the mean firing rates to remain within realistic bounds (νE ∈ [0.5, 5], νI1∈[10,25], νI2∈[3,15]). (TIF) [file pcbi.1006781.s006.tif]

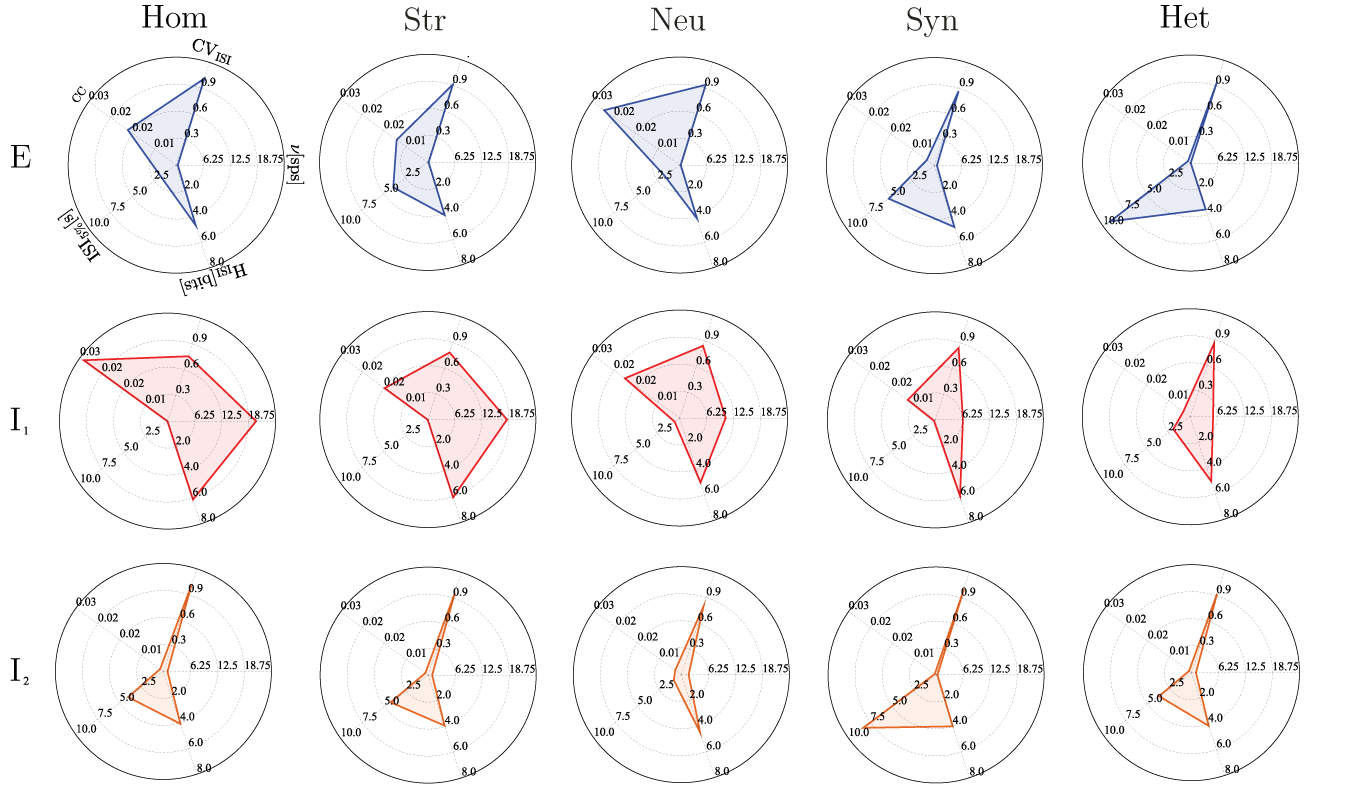

Supplement: S3 Fig — Complete statistics of population spiking activity in the active state for the different neuron classes: E (top, blue), I1 (middle, red) and I2 (bottom, orange) and for the different conditions (columns). The radial axes in each plot correspond to: regularity (CVISI), synchrony (CC), burstiness (ISI5%), entropy of the ISI distribution (HISI), and the mean firing rate (ν). All statistics were computed for an observation period of 10s, in a single realization for each condition, with all input parameters fixed and set to the values determined in S2 Fig. (TIF) [file pcbi.1006781.s007.tif]

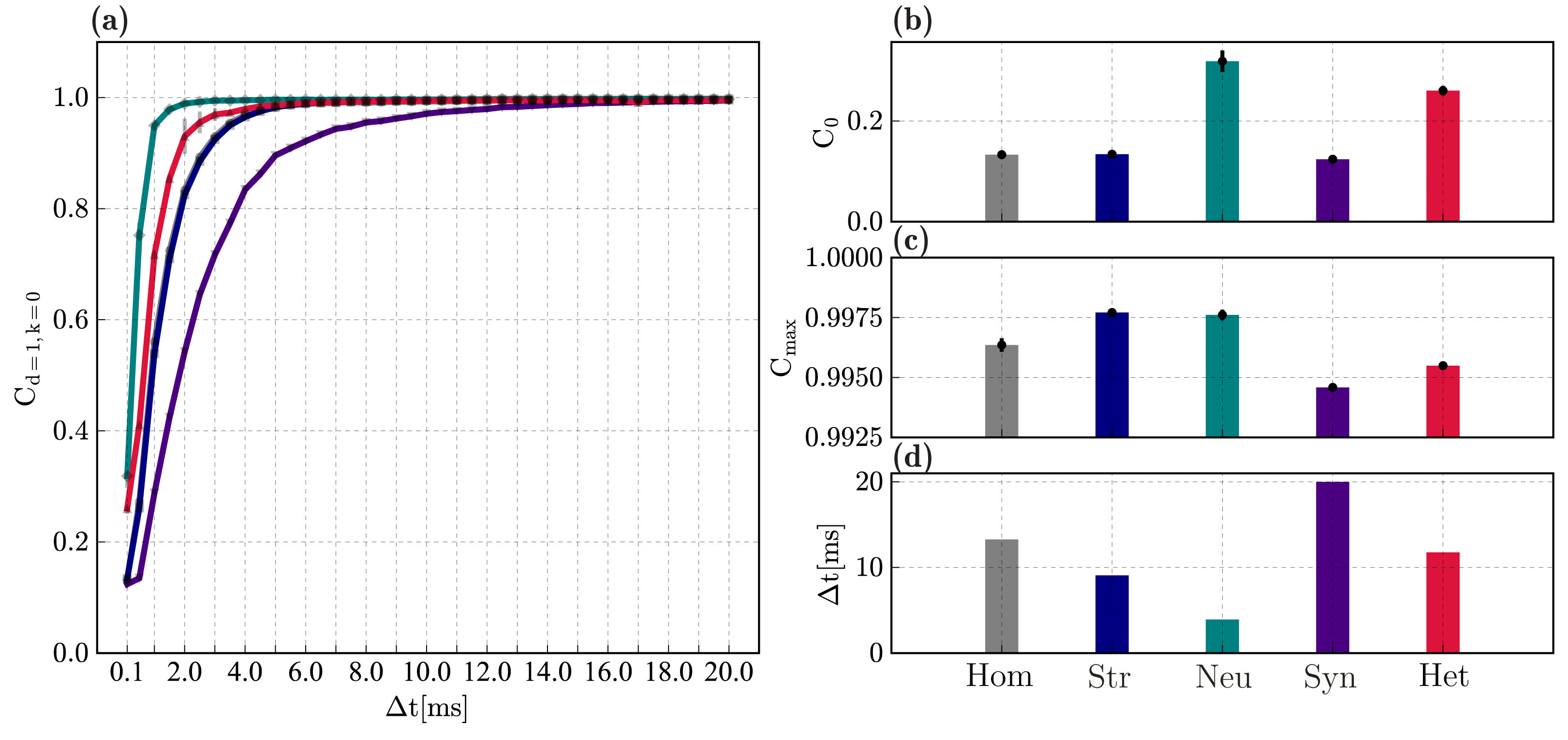

Supplement: S4 Fig — (a) capacity to reconstruct the original input signal at zero lag (i.e. maximum polynomial degree d = 1, maximum delay k = 0, Cd=1,k=0) as a function of the signal resolution (Δt). Since the capacity values converge asymptotically to 1, we determine the optimal resolution as the minimum Δt at which Cd=1,k=0 ≥ 0.99. (b) Decoding capacity at minimum resolution Δt = 0.1 ms (equal to the simulation resolution). (c) Capacity at the maximum resolution tested (Δt = 20 ms). (d) Optimal resolution for each condition. All results correspond to the mean and standard deviations for 10 simulations per condition. (TIF) [file pcbi.1006781.s008.tif]
